# New Eunicellin-Type Diterpenes from the Panamanian Octocoral *Briareum asbestinum*

**DOI:** 10.3390/md18020084

**Published:** 2020-01-27

**Authors:** Marcelino Gutiérrez, Ricardo Santamaría, José Félix Gómez-Reyes, Héctor M. Guzmán, Javier Ávila-Román, Virginia Motilva, Elena Talero

**Affiliations:** 1 Centro de Biodiversidad y Descubrimiento de Drogas, Instituto de Investigaciones Científicas y Servicios de Alta Tecnología (INDICASAT AIP), Panamá, Apartado 0843-01103, Republic of Panama; rsantamaria@indicasat.org.pa (R.S.); gomezjosefelix@gmail.com (J.F.G.-R.); 2 Smithsonian Tropical Research Institute, Balboa, Ancon, P. O. Box 0843-03092, Republic of Panama; guzmanh@si.edu; 3 Department of Biochemistry and Biotechnology, Faculty of Chemistry, Universitat Rovira i Virgili, 43007 Tarragona, Spain; franciscojavier.avila@urv.cat; 4 Department of Pharmacology, Faculty of Pharmacy, University of Seville, 41012 Seville, Spain; motilva@us.es

**Keywords:** asbestinins, briarellins, eunicellin diterpenes, *Briareum*, cytokines, cyclooxygenase-2

## Abstract

Gorgonian octocorals are considered a prolific source of secondary metabolites with a wide range of biological activities, including anti-inflammatory activity. In particular, the genus *Briareum* is known for producing a wealth of diterpenes with complex chemical structures. The chemical study of the methanolic extract of *Briareum asbestinum* collected in Bocas del Toro, on the Caribbean side of Panama, led to the isolation of three new eunicellin-type diterpenes: briarellin T (**1**), asbestinin 27 (**2**), asbestinin 28 (**3**) and the previously described asbestinin 17 (**4**). The structures of the new compounds were determined by extensive NMR analyses and HRMS. Anti-inflammatory activity assays showed a significant reduction of the pro-inflammatory cytokines TNF-α, IL-6, IL-1β and IL-8 as well as a downregulation of COX-2 expression in LPS-stimulated THP-1 macrophages. These findings support the potential use of these marine compounds as therapeutic agents in the treatment of inflammatory diseases.

## 1. Introduction

Acute inflammation is a biological response to pathogens, toxic agents, tissue damage or radiation. This process is a defense mechanism aimed to remove harmful stimuli and restore tissue homeostasis. Nevertheless, uncontrolled acute inflammation can trigger a chronic inflammatory process, which is related to several diseases, such as cancer, rheumatoid arthritis, asthma, inflammatory bowel disease, multiple sclerosis, or cardiovascular diseases [1]. Currently, many researchers are focused on the search for new anti-inflammatory molecules, since there is interest in finding more effective therapies with fewer side effects than those currently used.

Traditionally, terrestrial natural products have been extensively investigated due to their beneficial properties. However, the need of new therapeutic compounds has given rise to a vast number of studies in marine environments, a resource with huge potential for drug development [2,3]. Gorgonian octocorals are considered a rich source of secondary metabolites due to their capacity for producing a diversity of diterpenes with approximately 40 different carbon skeletons [4,5,6,7]. In particular, the genus *Briareum* produces diterpenes of the briarane class and the eunicellin-type, such as the briarellins and the asbestinins [4,5,6,7,8]. Briarellins and asbestinins along with the eunicellins (cladiellins) belong to a series of 2,11-cyclized cembranoids that share an oxatricyclo [6.6.1.02,7] pentadecane core. Briareins, briarellins and asbestinins have shown a wide range of biological activities including cytotoxic, antiviral, anti-inflammatory, antimicrobial, antifouling and antiprotozoal activities [5,8,9], revealing the pharmacological potential of these compound families. 

Along the Caribbean coast of Panama, two species of *Briareum* have been reported, *B. polyanthes* and *B. asbestinum*. Of these, *B. asbestinum* has been chemically studied, with our group reporting eunicellin-based diterpenes with inhibitory activity on the production of nitric oxide (NO) induced by lipopolysaccharide (LPS) in macrophages [10]. The aim of this research was to reexamine a diterpene-rich fraction of *B. asbestinum* collected in Bocas del Toro, Panama. Our findings led to the isolation and identification of four additional eunicellin-based diterpenes including the new briarellin T (**1**), two new asbestinins 27 and 28 (**2**–**3**), and the known asbestinin 17 (**4**). In addition, we described for the first time the anti-inflammatory properties of these compounds as inhibitors of the pro-inflammatory cytokines tumor necrosis factor alpha (TNF-α), interleukin 6 (IL)-6, IL-1β, and IL-8 as well as cyclooxygenase 2 (COX-2) expression in LPS-induced THP-1 macrophages. 

## 2. Results and Discussion

### 2.1. Isolation and Characterization of Diterpenes

**Figure 1:**
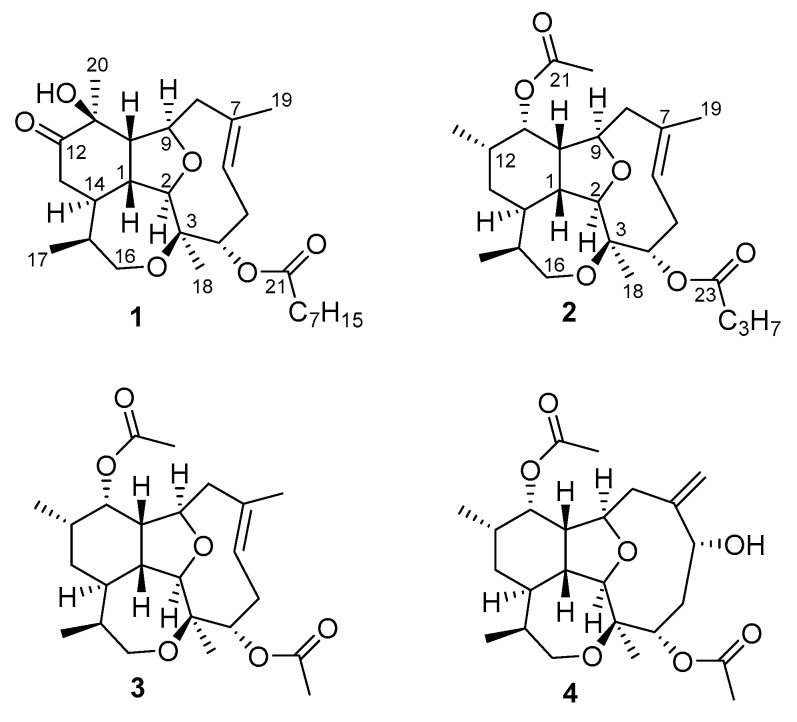
Structures of isolated compounds **1**–**4**.

The octocoral *Briareum asbestinum* was collected by hand using SCUBA at 10 m in Isla San Cristobal, Bocas del Toro, Panama. The coral was minced and extracted with n-hexane, ethyl acetate and methanol. The methanol extract was pre-fractionated using reverse phase solid phase extraction (SPE) and HPLC purification to yield compounds **1**–**4** (Figure 1). 

Briarellin T (**1**) was isolated as a glassy solid, and its molecular formula was determined as C_28_H_44_O_6_Na based on HR-ESITOFMS data (*m*/*z* 499.3014, [M + Na]^+^). Seven degrees of unsaturation were calculated from the molecular formula: three accounted for carbonyls of ester and ketone and one double bond, hence compound **1** possesses four rings. Detailed analysis of ^1^H and ^13^C NMR data indicated the structure of briarellin T (**1**) was similar to briarellin S, previously reported from our group [10]. ^13^C-NMR and DEPT spectra showed the presence of one ketone (δ_C_ 214.3), one ester carbonyl (δ_C_ 173.5), one double bond (δ_C_ 124.8, 129.9) and five methyl groups (δ_C_ 11.4, 19.6, 18.8, 22.6, 14.0) (Table 1). Additionally, resonances for six oxygenated carbon atoms corresponded to three methines (δ_C_ 95.3, 71.7, 79.5), one methylene (δ_C_ 66.9), and two quaternary (δ_C_ 80.3, 75.8). The remaining carbons corresponded to four sp^3^ methines (δ_C_ 37.8, 51.9, 38.0, 35.1), three sp^3^ methylenes (δ_C_ 29.0, 44.6, 38.9), and six methylenes characteristic of a fatty acid chain (δ_C_ 34.6, 25.1, 28.9, 28.8, 31.7, 22.6). 

**Figure 2:**
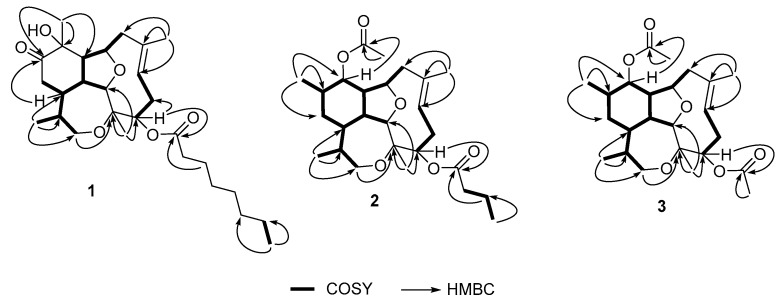
Correlation spectroscopy (COSY) and key heteronuclear multiple bond correlation (HMBC) of compounds **1**–**3**.

Analysis of the ^1^H-NMR spectrum evidenced signals of five methyl groups: two, attached to sp3 quaternary carbons bearing oxygen (δ_H_ 1.38, s; 1.43, s); one attached to a sp^3^ methine (δ_H_ 0.79, d, *J* = 7.1 Hz); one attached to a sp^2^ carbon (δ_H_ 1.88, s); and one corresponded with the terminus of a fatty acid chain (δ_H_ 0.88, t, *J* = 7.0 Hz) (Table 1). A branched spin system composed of one methyl group (δ_H_ 0.79, d), three diastereotopic methylenes (δ_H_ 3.30/3.78; 2.05/2.62; 2.34/2.41) and six methines (δ_H_ 1.88, 2.32, 2.53, 4.09, 2.31 and 4.69) was connected throughout COSY correlations (Figure 2). A separated spin system composed of one oxygenated methine (δ_H_ 5.33, m), one methylene (δ_H_ 2.14/2.76), and one olefinic proton (δ_H_ 5.37, m) was also assigned through COSY experiments. The remaining protons were consistent with the methylenes of a fatty acid chain (δ_H_ 2.34, m; 1.62, m; 1.29, m; 1.26, m). 

All functional groups and spin systems described above were connected through *J*^2,3^ HMBC correlations (Figure 2) as follows: methyl H-17 showed correlations to carbons C-14, C-15 and C-16; methyl H-18 showed correlations to carbons C-2, C-3 and C-4; methyl H-19 showed correlations with carbons C-6, C-7 and C-8; and methyl H-20 showed correlations with carbons C-10, C-11 and C-12. In this way the tetracyclic structure of the diterpenic nucleus was assembled. A *J*^3^ HMBC correlation observed between proton H-4 with carbonyl C-21 indicated the fatty acid moiety was attached to carbon C-4.

**Figure 3:**
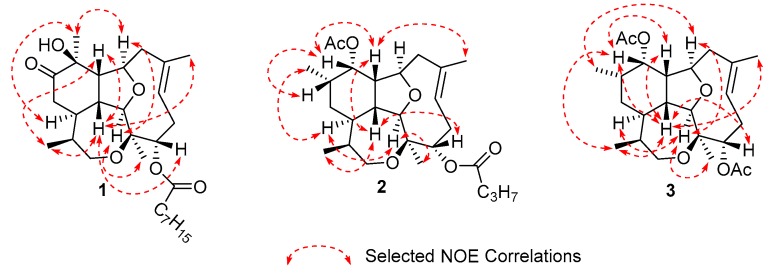
Selected 1D NOE correlations of compounds **1**–**3.**

The relative stereochemistry of compound **1** was assigned using 1D NOE experiments (Figure **3**). Irradiation of methyl H-20 produced a strong enhancement of protons H-2, H-9 and H-14, whereas irradiation on methyl H-18 produced an enhancement on proton H-2. All these protons (H-2, H-9, H-14 and H-20) were assigned arbitrarily with α-configuration. Irradiation of methyl H-17 produced a strong enhancement of protons H-1, H-10 and H-19, while irradiation of H-1 enhanced H-4, H-10, H-17 and H-19; hence H-1, H-4, H-10 and H-17 were assigned with β-configuration. The geometry of the C-6/C-7 double bond was determined as *E* based on the ^13^C NMR chemical shifts of carbons C-8 and C-19 [11,12]. Moreover, the enhancement of H-19 when H-17 and H-1 were irradiated confirmed the *E* geometry of the double bond.

Asbestinin 27 (**2**) was also isolated as a glassy solid. Its molecular formula was determined as C_26_H_40_O_6_K based on the interpretation of the HR-ESITOFMS data (*m*/*z* 487.2452, [M + K]^+^). Seven degrees of unsaturation were calculated from the molecular formula: three accounted for two carbonyls and one double bond, hence compound **2** was deduced to be tetracyclic.

Detailed analysis of the 1D and 2D NMR data (Table 2, Figure 2) revealed that compound **2** is a diterpene analog to asbestinin **1** [13]. Main differences consisted in the positions of the acetate and butyrate moieties attached to C-11 and C-4 of compound **2**, respectively (confirmed by HMBC data, Figure 2); instead of the acetate and butyrate moieties attached to C-4 and C-11 in asbestinin 1. Relative configuration of compound **2** was determined on the basis of NOE experiments as depicted in Figure 3.

Asbestinin 28 (**3**) was also isolated as a glassy solid. Its molecular formula was determined as C_24_H_36_O_6_K based on the interpretation of the HR-ESITOFMS data (*m*/*z* 459.2139, [M + K]^+^). Seven degrees of unsaturation were calculated from the molecular formula of asbestinin 28 (**3**): three unsaturations accounted for two carbonyls and one double bond, hence compound **3** was deduced to be tetracyclic as compounds **1** and **2**. 1D and 2D NMR data of compounds **2** and **3** were almost identical (Table 2, Figure 2 and Figure 3). The asbestinin core was the same in both compounds **2** and **3**. The main difference was the presence of an acetate group attached to C-4 in asbestinin 28 (**3**) instead of the butyrate moiety attached to the same carbon in asbestinin 27 (**2**). The lack of two methylenes (−28 Daltons) was evident in the molecular formula of compound **3** compared with compound **2**. Relative stereochemistry of compound **3** was determined on the basis on NOE experiments, as depicted in Figure 3.

### 2.2. Anti-Inflammatory Activity

Briarellins and asbestinins are biologically active marine natural products with great pharmacological potential due to their variety of properties, including antimicrobial, antiviral, antimalarial, and cytotoxic activities [9,14]. Moreover, a previous study from our group has reported that briarellin S reduced NO levels in LPS-stimulated primary murine macrophages [10]. However, there is no more evidence that reports the anti-inflammatory activity of these marine compounds. In the present study, the diterpenes obtained from the octocoral *Briareum asbestinum* were tested for their in vitro anti-inflammatory activity, by measuring the production of pro-inflammatory cytokines. 

**Figure 4:**
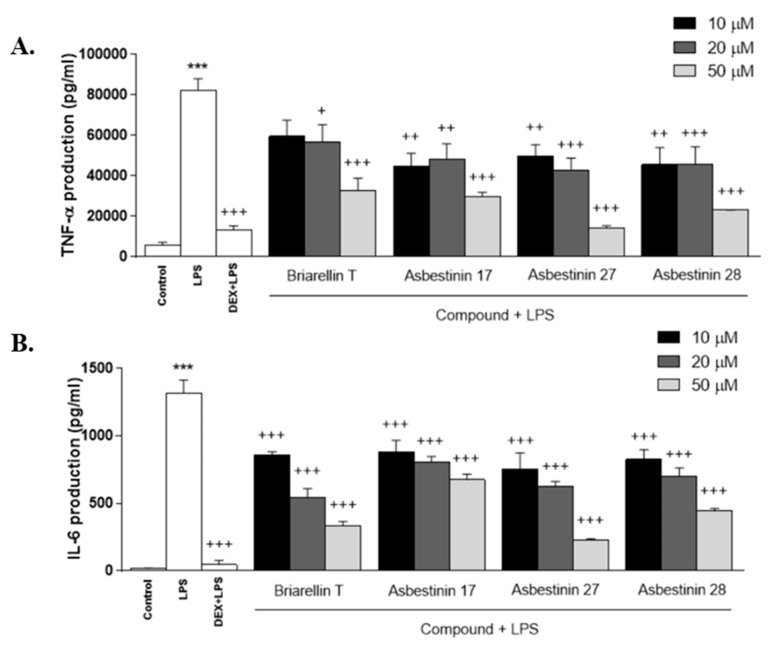

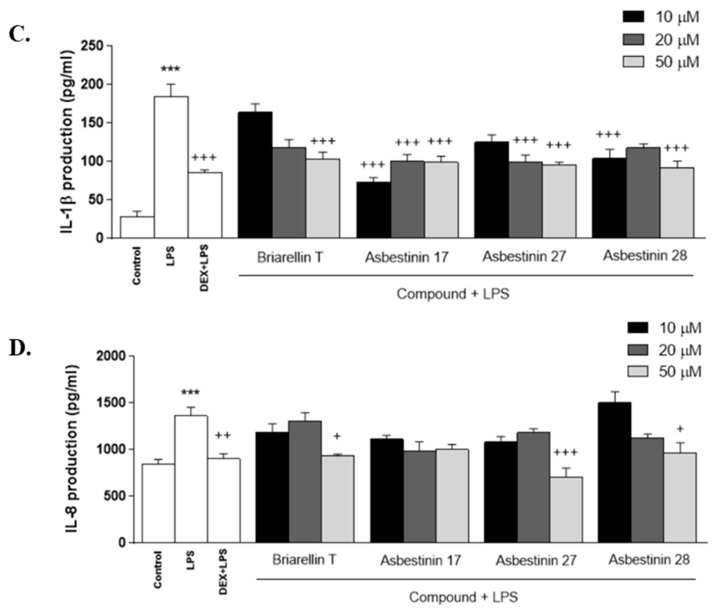
Effect of the diterpenes briarellin T, and asbestinin 17, 27 and 28 on lipopolysaccharide (LPS)-induced TNF-α (**A**), IL-6 (**B**), IL-1β (**C**) and IL-8 (**D**) production by THP-1 macrophages. Cells (10^5^ cells/mL) were pre-treated with diterpenes (10, 20 and 50 μM) or dexamethasone (Dex, 1 μM) for 1 h. Afterwards, the macrophages were stimulated with LPS (1 μg/mL) for 24 h. Data are the means ± SEM of four independent experiments. Mean value was significantly different compared with the unstimulated group (*** *p* <0.001; Student t test). Mean value was significantly different compared with LPS-stimulated cells (+ *p* <0.05, ++ *p* <0.01, +++ *p* <0.001; one-way ANOVA followed by Bonferroni’s Multiple Comparison test).

Assays were conducted on human THP-1 monocytes transformed into macrophages, which are a useful in vitro model to test anti-inflammatory molecules, since their stimulation by LPS induces pro-inflammatory mediator’s production [15]. In order to select non-cytotoxic concentrations of the compounds, THP-1 cell viability was carried out by using the SRB assay and a maximum concentration of 50 μM was assayed (Table S1). THP-1 macrophages were pretreated with the compounds at 10, 20 and 50 µM for 1 h, and then stimulated with LPS (1 μg/mL) for 24 h. As shown in Figure 4, LPS induced a significant increase in the production of TNF-α, IL-6 and IL-1β in THP-1 cells in relation to unstimulated control cells (*p* < 0.001). These molecules are potent pro-inflammatory cytokines produced by immune cells at the site of inflammation, and play a crucial role in local and systemic inflammatory responses [16]. Dexamethasone, used as an anti-inflammatory reference drug, strongly inhibited LPS-induced TNF-α, IL-6, and IL-1β production. Similarly, pretreatment of cells with all the diterpenes substantially reduced cytokine levels; these anti-inflammatory effects were more marked at the concentration of 50 μM (*p* < 0.001) (Figure 4A–C). The cytokine IL-8 is a potent chemoattractant agent for neutrophils as well as regulates angiogenesis and metastasis [14]. Treatment with the compounds briarellin T (**1**), asbestinin 27 (**2**) and asbestinin 28 (**3**) resulted in a significant suppression of cytokine levels at the highest concentration (*p* < 0.05, *p* < 0.001 and *p* < 0.05, respectively), comparable to dexamethasone (*p* < 0.01) (Figure 4D).

**Figure 5:**
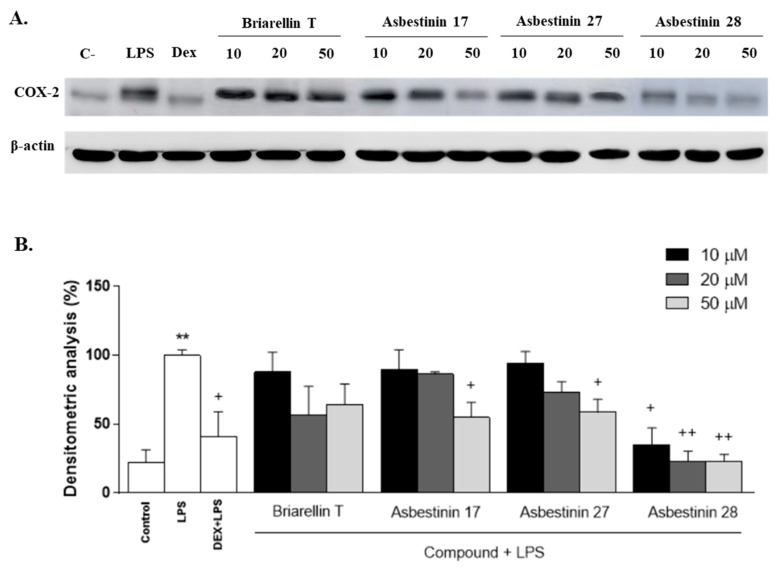
Effect of the diterpenes briarellin T, and asbestinin 17, 27, and 28 on lipopolysaccharide (LPS)-induced cyclooxygenase-2 (COX-2) expression by THP-1 macrophages. Cells (10^6^ cells/mL) were pre-treated with the diterpenes (10, 20 and 50 μM) or dexamethasone (Dex, 1 μM) for 1 h. Afterwards, the macrophages were stimulated with LPS (1 μg/mL) for 24 h. (**A**) Representative Western Blot analysis of COX-2 protein. (**B**) Densitometric data were studied after normalization to the housekeeping gene, β-actin. Data are the means ± SEM from four independent experiments. Mean value was significantly different compared with the unstimulated group (** *p* <0.01; Student t test). Mean value was significantly different compared with LPS-stimulated cells (+ *p* <0.05, ++ *p* <0.01; one-way ANOVA followed by Bonferroni’s Multiple Comparison test).

The inducible enzyme COX-2 has also been reported to have a key role in the inflammatory response by overproducing pro-inflammatory prostaglandins such as PGE_2_ [17]. In order to elucidate the possible mechanism by which these kind of diterpenes exerts their anti-inflammatory effects, we examined COX-2 expression by Western Blotting. THP-1 macrophages were pretreated with the compounds (10, 20 and 50 μM) for 1h, and then stimulated with LPS (1 μg/mL). As presented in Figure 5, exposure of THP-1 cells to LPS induced a pronounced increase in COX-2 protein levels compared with unstimulated THP-1 macrophages (*p* < 0.01). As expected, the reference compound dexamethasone markedly inhibited the expression of this enzyme (*p* < 0.05). LPS-induced COX-2 expression was significantly attenuated after pretreatment with the compounds asbestinin 17 (**4**) and asbestinin 27 (**2**) at 50 μM (*p* < 0.05). The compound asbestinin 28 (**3**) exhibited the highest inhibitory activity, reducing COX-2 levels with all concentrations used. Interestingly, this compound drastically decreased protein expression to basal levels at 20 and 50 μM (*p* < 0.01).

## 3. Materials and Methods 

### 3.1. General Experimental Procedures 

Optical rotations were measured on a JASCO P-2000 polarimeter (JASCO, Easton, MD, USA). IR data was collected using a Bruker Alpha Fourier transform infrared spectrophotometer (Bruker, Billerica, MA, USA). NMR spectra were measured at ^1^H resonance frequency on a Jeol Eclipse + 400 MHz spectrometer (JEOL, Peabody, MA, USA). Chemical shifts were calibrated internally to the residual signal of deuterated chloroform (CDCl_3_ δ_H_ 7.26, δ_C_ 77.0). For NMR measurements concentration used was in the range of 3–8 mg of compound (depending on the amount of compound available) dissolved in 600 μL of deuterated chloroform. High-resolution mass spectra were obtained on a Bruker micrOTOF-Q III (Bruker Daltonics, Billerica, MA, USA). HPLC separations were performed using an Agilent 1200 HPLC system equipped with a quaternary pump, a diode array detector (Agilent, Santa Clara, CA, USA) and a normal phase silica gel column (Phenomenex Sphereclone^®^, 4.6 mm × 100 mm, 5 µm). Reverse phase solid phase extraction (SPE) separation was carried out using SUPELCO Supelclean™ LC-18 (C-18, octadecyl) solid-phase extraction cartridges (Supelco^®^ Analytical, Bellefonte, PA, USA).

### 3.2. Biological Material 

The octocoral *Briareum asbestinum* (Order Alcyonacea, Family Briaridae) was collected by hand using SCUBA at 10 m in Isla San Cristobal, located in the Caribbean Sea off the coast of Bocas del Toro, Panama in October 2014. The coral specimen was identified as *Briareum asbestinum* (Pallas) based on its morphology and SEM-micrographs of the coral sclerites at the Smithsonian Tropical Research Institute (Ancon, Panama). A reference specimen is deposited at INDICASAT Center for Biodiversity and Drug Discovery under the number GLBO-061014-02. 

### 3.3. Extraction and Isolation 

The fresh organism (1242 g) was minced and exhaustively extracted with *n*-hexane, ethyl acetate and methanol. The organic extracts were evaporated separately in vacuo to give 4.1 g, 24.7 g and 28.6 g of the *n*-hexane, ethyl acetate and methanol extracts, respectively. 

The methanolic extract was fractionated using a reverse phase solid phase extraction (SPE) cartridge (1.0 g) eluted sequentially with 20, 40, 60, 80, and 100 % of methanol in water, obtaining five fractions (F1-F-5). ^13^C-NMR spectra of fraction F4 (80% MeOH in water, 1.7 g) revealed the presence of diterpenes with asbestinin and briarellin skeletons. Hence fraction F4 (53.0 mg) was purified by normal phase HPLC using a Phenomenex Sphereclone^®^ column (250 × 10 mm) eluted with a gradient of 40%–100% of EtOAc in *n*-hexane, in 70 min at 1 mL/min, to yield 17 sub-fractions denoted as FI-FXVII. Fraction FV contained asbestinin 27 (**2**, 3.2 mg), fraction FIX contained asbestinin 28 (**3**, 12.7 mg), fraction FXI contained briarellin T (**1**, 8.7 mg) and fraction FXV contained asbestinin 17 (**4**, 5.1 mg). 

**Briarellin T (1):** Glassy solid; ${\left[\alpha \right]}_{D}^{20}$ +70 (*c* 0.1, MeOH); IR (film) ν_max_ 3422, 2962, 2853, 1724, 1659, 1349, 1165, 1107, 999 cm^−1^; ^1^H and ^13^C NMR see Table 1; HRESI-TOF-MS *m*/*z* [M + Na]^+^ 499.3014 (calcd for C_28_H_44_O_6_Na, 499.3030).

**Asbestinin 27 (2):** Glassy solid; ${\left[\alpha \right]}_{D}^{20}$ −103.3 (*c* 0.03, MeOH); IR (film) ν_max_ 1664, 1403, 1106, 1013 cm^−1^; ^1^H and ^13^C NMR see Table 2; HRESI-TOF-MS *m*/*z* [M + K]^+^ 487.2452 (calcd for C_26_H_40_O_6_K, 487.2456).

**Asbestinin 28 (3):** Glassy solid; ${\left[\alpha \right]}_{D}^{20}$ +77.9 (*c* 0.2, MeOH); IR (film) ν_max_ 1726, 1378, 1107, 998 cm^−1^; ^1^H and ^13^C NMR see Table 2; HRESI-TOF-MS *m*/*z* [M + K]^+^ 459.2139 (calcd for C_24_H_36_O_6_K, 459.2143). 

### 3.4. Cell Culture

Human acute monocytic leukemia cell line THP-1 was kindly provided by Professor Francisco Muriana of the Fat Institute (Consejo Superior de Investigaciones Científicas, Seville, Spain). Cells were maintained in RPMI 1640 media (GIBCO^®^, Life Technologies, New York, NY, USA) containing 10% heat-inactivated fetal bovine serum, 100 U/mL penicillin and 100 μg/mL streptomycin (PAA, Pasching, Austria), in a humidified atmosphere containing 5% CO_2_ at 37 °C. 

### 3.5. Cell Proliferation Assay

Cellular viability upon exposure to terpenes was evaluated by using the sulforhodamine B (SRB) method (Sigma-Aldrich Química, S.A., Spain) [18]. Briefly, the cells were seeded into 96-well plates at a density 10^5^cells/mL (100 µL/well) and differentiated into macrophages with phorbol myristate acetate (PMA, Sigma-Aldrich Química, S.A., Madrid, Spain) at 0.2 μM for 72 h in a humidified atmosphere of 5% CO_2_ at 37 °C. The medium was then removed and cells were washed with cold phosphate saline buffer (PBS). Afterwards, macrophages were incubated for 24 h with different concentration of diterpenes (6.25, 12.5, 25, 50, and 100 µM). Compounds were prepared by dilution of stock solutions (10 mM) in dimethylsulfoxide (DMSO, Barcelona, Spain) and culture medium. Controls were incubated in medium with 0.1% *v*/*v* DMSO, not affecting cell viability. Next, the cells were fixed using 50 μL of 50% trichloroacetic acid (4 °C, 1 h) and washed several times with distilled water. After that, macrophages were stained with 0.4% SRB solution (50 μL) prepared in 1% acetic acid for 30 min at room temperature. After this time, the SRB solution was removed and the plates were washed with 1% acetic acid and air-dried. Finally, 10 mM Tris-base (100 μL) was added into each well to solubilize the cell-bound dye. The absorbance at 540 nm was measured in a microplate spectrophotometer (Sinergy HT, Biotek^®^, Bad Friedrichshall, Germany). 

### 3.6. Determination of Cytokines Production

THP-1 monocytes (10^5^ cells/mL, 100 µL/well) were transformed into macrophages in presence of PMA (0.2 μM) in 96-well plates in a humidified atmosphere of 5% CO_2_ at 37 °C for 72 h. Afterwards, the medium was removed and the cells were washed twice with cold PBS. Next, cells were pre-treated with diterpenes (10, 20, and 50 μM) or the reference drug dexamethasone (1 μM), for 1 h. Subsequently, macrophages were stimulated with LPS from *Escherichia coli* (1 μg/mL) for 24 h. Control groups (unstimulated and stimulated with LPS) were incubated with culture medium with DMSO (0.1% *v*/*v*). After incubation period, the supernatants were collected and kept at −80 °C until cytokines (TNF-α, IL-6, IL-1β and IL-8) measurements could be collected by commercial enzyme-linked immunosorbent assay (ELISA) kits (Diaclone GEN-PROBE, France), according to the manufacturer’s protocol. The absorbance was determined at 450 nm with a microplate reader (Labsystems Multiskan EX, Thermo Scientific, New York, NY, USA). To calculate the cytokines concentration, a standard curve was created using serial dilutions of cytokine standards provided with the kit.

### 3.7. Isolation of Cytoplasmic Proteins and Analysis of Proteins Expression by Western Blot Assay

THP-1 monocytes (10^6^ cells/mL, 2 mL/well) were transformed into macrophages in presence of 0.2 μM PMA in 6-well plates, for 72 h, in a humidified atmosphere of 5% CO_2_ at 37 °C. Then, the medium was removed and the cells were washed with cold PBS. Subsequently, macrophages were pre-treated with diterpenes at concentrations of 10, 20, and 50 μM for 1 h and then stimulated with LPS (1 μg/mL) for 24 h. Dexamethasone (1 μM) was used as the positive reference compound. Control groups (unstimulated and stimulated with LPS) were incubated in culture medium with DMSO 0.1% (*v*/*v*). Then, cell pellets were mixed with cold lysis buffer (50 mM Tris-HCl pH 7.5, 8 mM MgCl_2_, 5 mM ethylene glycol bis (2-aminoethyl ether)-*N*,*N*,*N′N′*-tetraacetic acid, 0.5 mM EDTA, 1 mM phenylmethylsulfonyl fluoride, 250 mM NaCl, 0.01 mg/mL leupeptin, 0.01 mg/mL pepstatin and 0.01 mg/mL aprotinin). Next, cells were scraped and incubated on ice for 30 min. Finally, cell lysates were sonicated, spined (12,000 g, 4 °C) for 10 min and stored at −80 °C. Protein concentration of the homogenates was evaluated using the Bradford assay [19]. Aliquots of the supernatants containing 50 μg of protein were separated on acrylamide gel (10%) by sodium dodecyl sulphate polyacrylamide gel electrophoresis. Then, the proteins were transferred onto a nitrocellulose membrane and incubated with the primary antibody against COX-2 (1:5000, Cayman Chemical, Michigan, USA) at 4 °C overnight. Next, the blots were washed three times for 15 min and incubated with the HRP-labeled secondary antibody ((1:5000, Pierce Chemical Company, Rockford, IL, USA) at room temperature for two hours. To prove equal loading, the membranes were incubated with an anti β-actin antibody (1:1000, Sigma Aldrich, St. Louis, MO, USA). Immunodetection was observed using an enhanced chemiluminescence light-detecting kit (Super-Signal West Pico Chemiluminescent Substrate; Thermo Scientific, New York, NY, USA). Densitometric data were evaluated after normalization to the housekeeping gene β-actin. The bands were analysed and quantified with a Scientific Imaging Systems (Biophotonics ImageJ Analysis Software; National Institute of Mental Health, Bethesda, MD, USA).

### 3.8. Statistical Analysis

All values in the figures and text are expressed as arithmetic means ± standard error of the mean (S.E.M.). Experiments were carried out in quadruple. Data were evaluated with GraphPad Prism^®^ software (Version No. 8, GraphPad Software, Inc., San Diego, CA, USA). Statistical significance between the two control groups (Control vs. LPS) was determined by Student’s t test. Statistical significance of any difference in each parameter between several groups was evaluated by one-way analysis of variance (ANOVA) followed by Bonferroni test. *p* values of <0.05 were considered statistically significant. 

## 4. Conclusions

Four eunicellin-based diterpenes: a briarellin (**1**) and three asbestinins (**2**–**4**) were isolated from the octocoral *Briareum asbestinum* and their structures determined by spectroscopic means. Compounds **1**–**3** are described for the first time. Moreover, we have demonstrated for the first time the anti-inflammatory activity of briarellin and asbestinin-type diterpenes through downregulation of the pro-inflammatory cytokines TNF-α, IL-6, IL-1β and IL-8 as well as reduction of COX-2 expression in LPS-induced THP-1 macrophages. These studies support the potential use of these compounds as therapeutic agents in the treatment of inflammatory pathologies. Further investigations are needed to elucidate the mechanism of action of this interesting class of diterpenes. 

## Figures and Tables

**Table 1:** NMR spectroscopic data of briarellin T (**1**) acquired in CDCl_3_ at 400 MHz.

Position	δ_C_, mult. ^a^	δ_H_, mult. (*J* in Hz) ^b^	HMBC	COSY
1	37.8, CH	2.53, dt (10.7, 8.5)	11	2, 10, 14
2	95.3, CH	4.09. d (8.5)	4, 9, 14, 18	1
3	80.3, C ^a^			
4	71.7, CH ^a,b^	5.33, m (overlap)	5, 21	5
5a	29.0, CH_2_	2.76, ddd (14.6, 9.2, 6.3)		4, 6
5b		2.14, m (overlap)		
6	124.8, CH	5.36, m (overlap)	8, 19	5
7	129.9, C			
8a	44.6, CH_2_	2.62, dd (13.5, 6.8)	19	9
8b		2.05, m (overlap)		
9	79.5, CH	4.64, dd (6.6, 2.4)	2, 7, 10	8, 10
10	51.9, CH	2.32, dt (8.7, 8.0, 2.7)		1, 9
11	75.8, C			
12	214.3, C			
13a	38.9, CH_2_	2.41, m	12, 14	14
13b		2.34, m		
14	38.0, CH	2.32, m (overlap)	1, 12,	1, 13, 15
15	35.1, CH	1.88, s		14, 16, 17
16a	66.9, CH_2_	3.78, dd (13.7, 3.6)	3, 17	15
16b		3.30, dd, (13,7, 6.2)		
17	11.4, CH_3_	0.79, d (7.1)	14, 15, 16	15
18	20.5, CH_3_	1.38, s	2, 3, 4	
19	18.8, CH_3_	1.89, s	6, 7, 8	
20	22.6, CH_3_	1.43, s	10, 11, 12	
21	173.5, C			
22	34.6, CH_2_	2.34, m		
23	25.1, CH_2_	1.62, m	21	
24	28.9 *, CH_2_	1.29, m		
25	28.9 *, CH_2_	1.29, m		
26	31.7, CH_2_	1.25, m		
27	22.6, CH_2_	1.27, m		28
28	14.0, CH_3_	0.88, t (7.0)	26, 27	27

^a^ δ_C_ values were obtained by the assistance of the HMBC correlations; ^b^ δ_C_ values were obtained by the assistance of the heteronuclear single-quantum correlation spectroscopy (HSQC); * This values can be exchanged.

**Table 2:** NMR spectroscopic data of asbestinins 27 (**2**) and 28 (**3**) acquired in CDCl_3_ at 400 MHz.

Position	Asbestinin 27 (2)		Asbestinin 28 (3)	
	δ_C_, mult. ^a^	δ_H_, mult. (*J* in Hz) ^b^	δ_C_, mult.	δ_H_, mult. (*J* in Hz)
1	38.3, CH	2.27, m	38.3, CH	2.27, dt (10.8, 8.3)
2	94.6, CH	4.04. d (8.5)	94.6, CH	4.01. m
3	79.1, C		79.1, C	
4	72.4, CH	5.33, m	72.7, CH	5.31, m
5a	29.1, CH_2_	2.71, dt (14.9, 8.1)	29.1, CH_2_	2.71, ddd (14.7, 9.3, 6.2)
5b		2.08, m		2.08, m
6	125.1, CH	5.32, m	124.9, CH	5.31, m
7	129.4, C		129.4, C	
8a	44.3, CH_2_	2.47, dd (13.4, 6.5)	44.3, CH_2_	2.47, dd (13.3, 6.5)
8b		2.02, m		2.02, m
9	80.9, CH	4.06, dd (8.9, 2.5)	80.9, CH	4.04, m
10	48.4, CH	2.09, m	48.3, CH	2.08, m
11	73.8, CH	5.30, m	73.8, CH	5.31, m
12	31.0, CH	1.97, m	31.0, CH	1.97, m
13a	31.3, CH_2_	1.47, dt (13.6, 9.8)	31.3, CH_2_	1.47, dt (13.6, 9.8)
13b		0.98, m		1.00, dt (13.6, 2.7)
14	37.7, CH	1.87, m	37.7, CH	1.90, m
15	36.7, CH	1.69, m	36.7, CH	1.73, m
16a	67.6, CH_2_	3.84, dd (13.3, 2.4)	67.7, CH_2_	3.85, dd (13.5, 2.4)
16b		3.36, dd, (13.3, 5.1)		3.37, dd, (13.5, 5.0)
17	11.4, CH_3_	0.76, d (7.1)	11.3, CH_3_	0.76, d (7.1)
18	19.7, CH_3_	1.35, s	19.7, CH_3_	1.34, s
19	18.8, CH_3_	1.87, s	18.8, CH_3_	1.86, s
20	18.1, CH_3_	0.91, d (7.2)	18.1, CH_3_	0.91, d (7.1)
21	171.4, C		171.4, C	
22	21.3, CH_3_	2.11, s	21.4, CH_3_	2.11, s
23	173.3, C		170.7, C	
24	36.6, CH_2_	2.30, t (7.3)	21.3, CH_3_	2.07, s
25	18.6, CH_2_	1.66, m		
26	13.6 CH_3_	0.96, t (7.4)		

^a^ δ_C_ values were obtained by the assistance of the HMBC correlations; ^b^ δ_C_ values were obtained by the assistance of the HSQC correlations.

## Supplementary Materials

The following are available online at https://www.mdpi.com/1660-3397/18/2/84/s1, Figures S1–S24: 1D, 2D NMR and HRMS data of isolated compounds.
