# Supplementary material for: New Eunicellin-Type Diterpenes from the Panamanian Octocoral Briareum asbestinum
Source: Mar Drugs. 2020 Jan 27;18(2):84. doi: 10.3390/md18020084 (PMC7074288; doi:10.3390/md18020084)
Supplement: Supplementary file 1 [file marinedrugs-18-00084-s001.pdf]

Supplementary data

New Eunicellin-type Diterpenes from the Panamanian Octocoral *Briareum asbestinum*

Marcelino Gutiérrez [1,*], Ricardo Santamaría [1], José Félix Gómez-Reyes [1], Héctor M. Guzmán [2], Javier Ávila-Román [3], Virginia Motilva [4], Elena Talero [4,*]

[1] Centro de Biodiversidad y Descubrimiento de Drogas, Instituto de Investigaciones Científicas y Servicios de Alta Tecnología (INDICASAT AIP), Panamá, Apartado 0843-01103, República de Panamá; rsantamaria@indicasat.org.pa (R.S.), gomezjosefelix@gmail.com (J.G.)

[2] Smithsonian Tropical Research Institute, Balboa, Ancon, P. O. Box 0843-03092, Republic of Panama; guzmanh@si.edu

[3] Department of Biochemistry and Biotechnology, Faculty of Chemistry, Universitat Rovira i Virgili, Tarragona 43007, Spain; franciscojavier.avila@urv.cat (J. A-R)

[4] Department of Pharmacology, Faculty of Pharmacy, University of Seville, Seville 41012, Spain; motilva@us.es (V.M.); etalero@us.es (E.T.)

Contents

Figure S1. Briarellin T, ^{1}H NMR spectrum

Figure S2. Briarellin T, ^{13}C NMR spectrum

Figure S3. Briarellin T, DEPT-135 spectrum

Figure S4. Briarellin T, COSY spectrum

Figure S5. Briarellin T, HSQC spectrum

Figure S6. Briarellin T, HMBC spectrum

Figure S7. Briarellin T, NOE spectra

Figure S8. Briarellin T, HR-ESITOFMS spectra

Figure S9. Asbestinin 27, ^{1}H NMR spectrum

Figure S10. Asbestinin 27, ^{13}C NMR spectrum

Figure S11. Asbestinin 27, DEPT-135 spectrum

Figure S12. Asbestinin 27, COSY spectrum

Figure S13. Asbestinin 27, HSQC spectrum

Figure S14. Asbestinin 27, HMBC spectrum

Figure S15. Asbestinin 27, NOE spectra

Figure S16. Asbestinin 27, HR-ESITOFMS spectra

Figure S17. Asbestinin 28, ^{1}H NMR spectrum

Figure S18. Asbestinin 28, ^{13}C NMR spectrum

Figure S19. Asbestinin 28, DEPT-135 spectrum

Figure S20. Asbestinin 28, COSY spectrum

Figure S21. Asbestinin 28, HSQC spectrum

Figure S22. Asbestinin 28, HMBC spectrum

Figure S23. Asbestinin 28, NOE spectra

Figure S24. Asbestinin 28, HR-ESITOFMS spectrum

Table S1. Viability of THP-1 human macrophages treated with different concentrations of diterpenes.

Figure S1. Briarellin T, ¹H NMR spectrum

```
Filename           = JF1_65_11_CDCl3  30_7_1[illegible]
Author             = DELTA
Experiment         = single_pulse.exp
Sample_Id          = I3_062_003
Solvent            = CHLOROFORM-D
Actual_Start_Time  = 28-JUL-2015 14:01:55
Revision_Time      = 31-JUL-2015 07:40:50

Comment            = Single Pulse Experiment
Data_Format        = 1D COMPLEX
Dim_Size           = 16384
X_Domain           = 1H
Dim_Title          = 1H
Dim_Units          = [ppm]
Dimensions         = X
Site               = Eclipse+ 400
Spectrometer       = DELTA_NMR

Field_Strength     = 9.389766[T] (400[MHz])
X_Acq_Duration     = 2.7312128[s]
X_Domain           = 1H
X_Freq             = 399.78219838[MHz]
X_Offset           = 5[ppm]
X_Points           = 16384
X_Prescans         = 0
X_Resolution       = 0.36613771[Hz]
X_Sweep            = 5.99880024[kHz]
Clipped            = FALSE
Scans              = 32
Total_Scans        = 32

Relaxation_Delay   = 4[s]
Recvr_Gain         = 16
Temp_Get           = 21.9[dC]
X_90_Width         = 9.81[us]
X_Acq_Time         = 2.7312128[s]
X_Angle            = 45[deg]
X_Pulse            = 4.905[us]
Initial_Wait       = 1[s]
Unblank_Time       = 2[us]
```

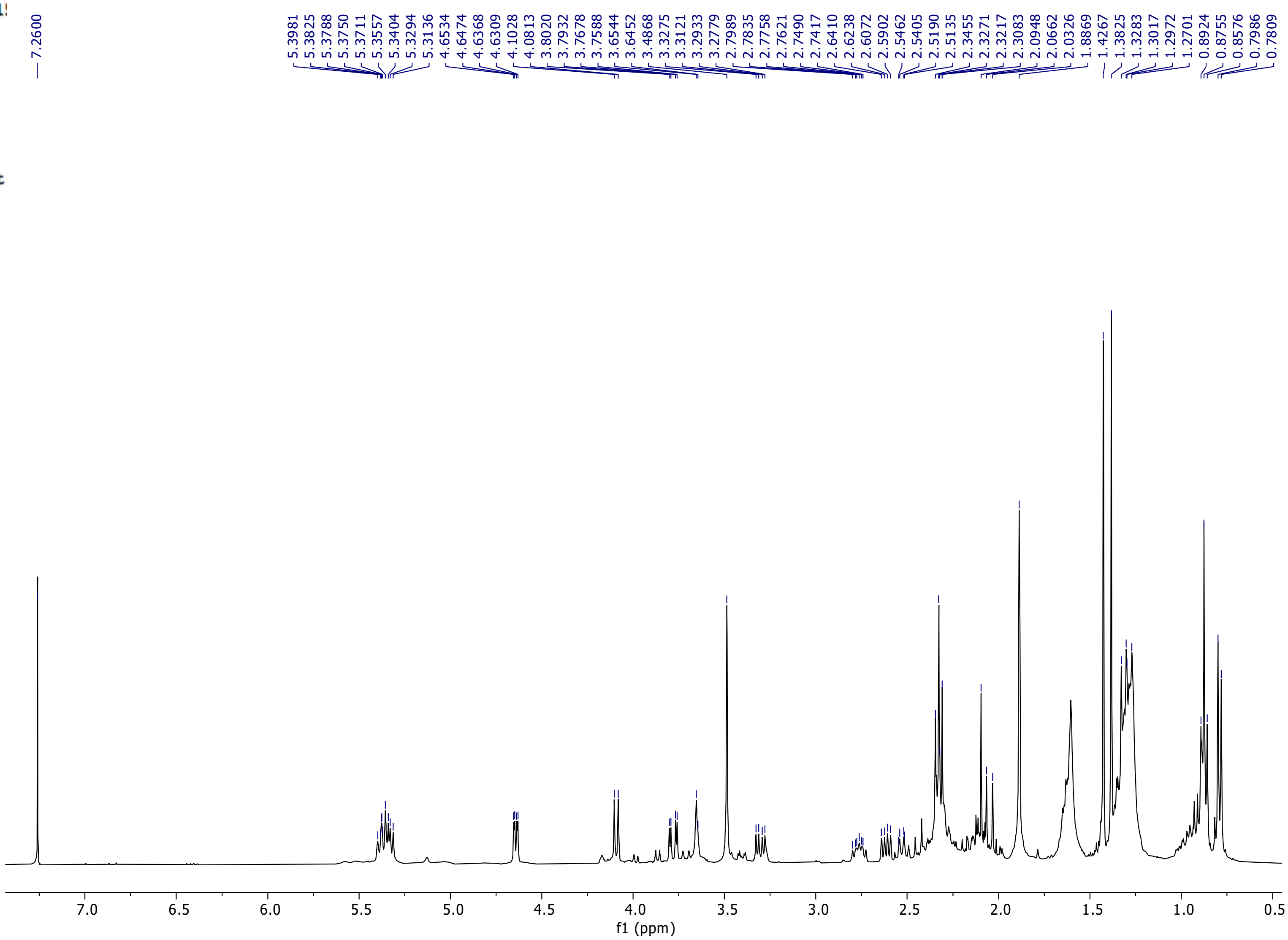

Figure S1. Briarellin T, ^{1}H NMR spectrum (expanded)

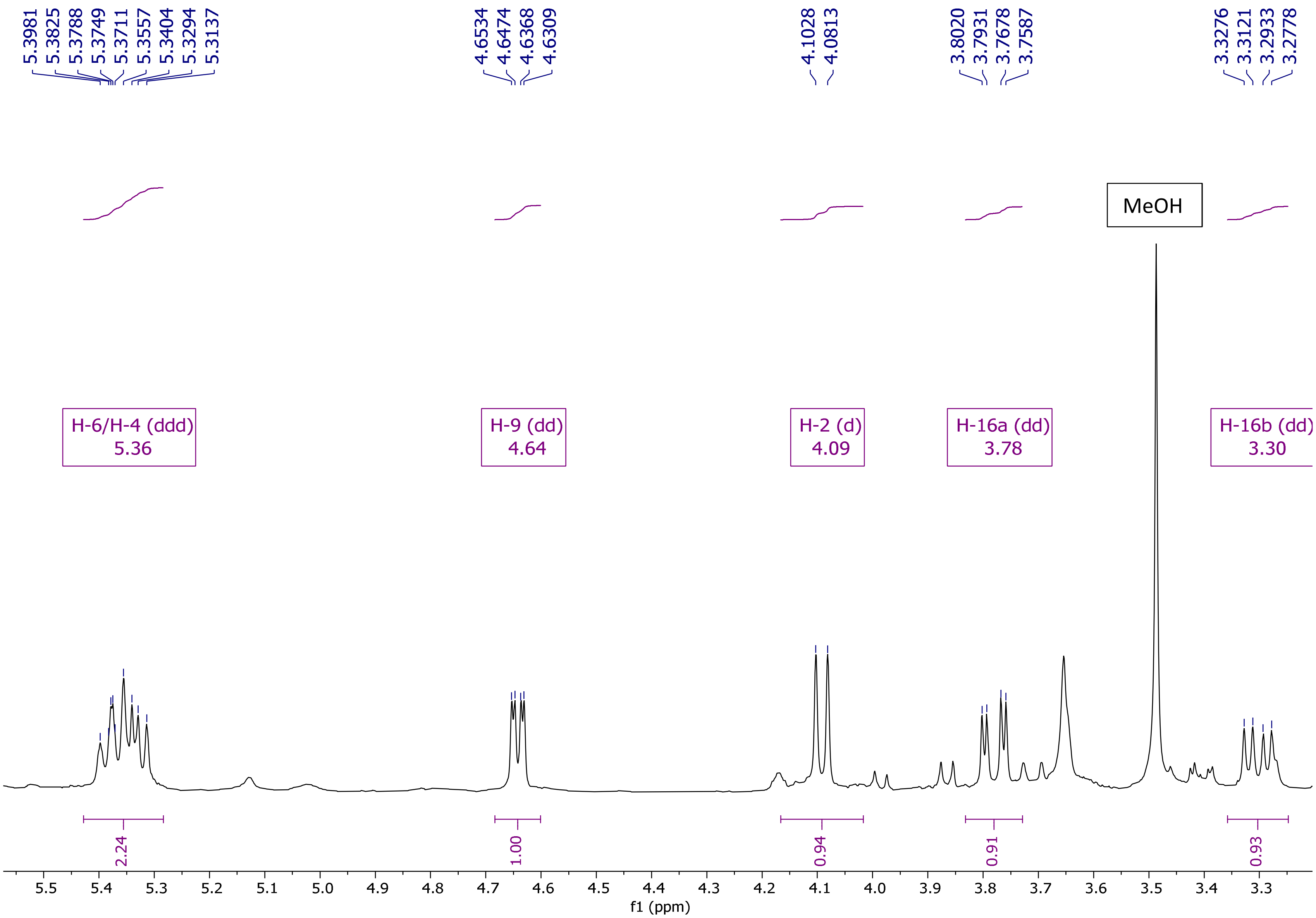

Figure S1. Briarellin T, ^{1}H NMR spectrum (expanded)

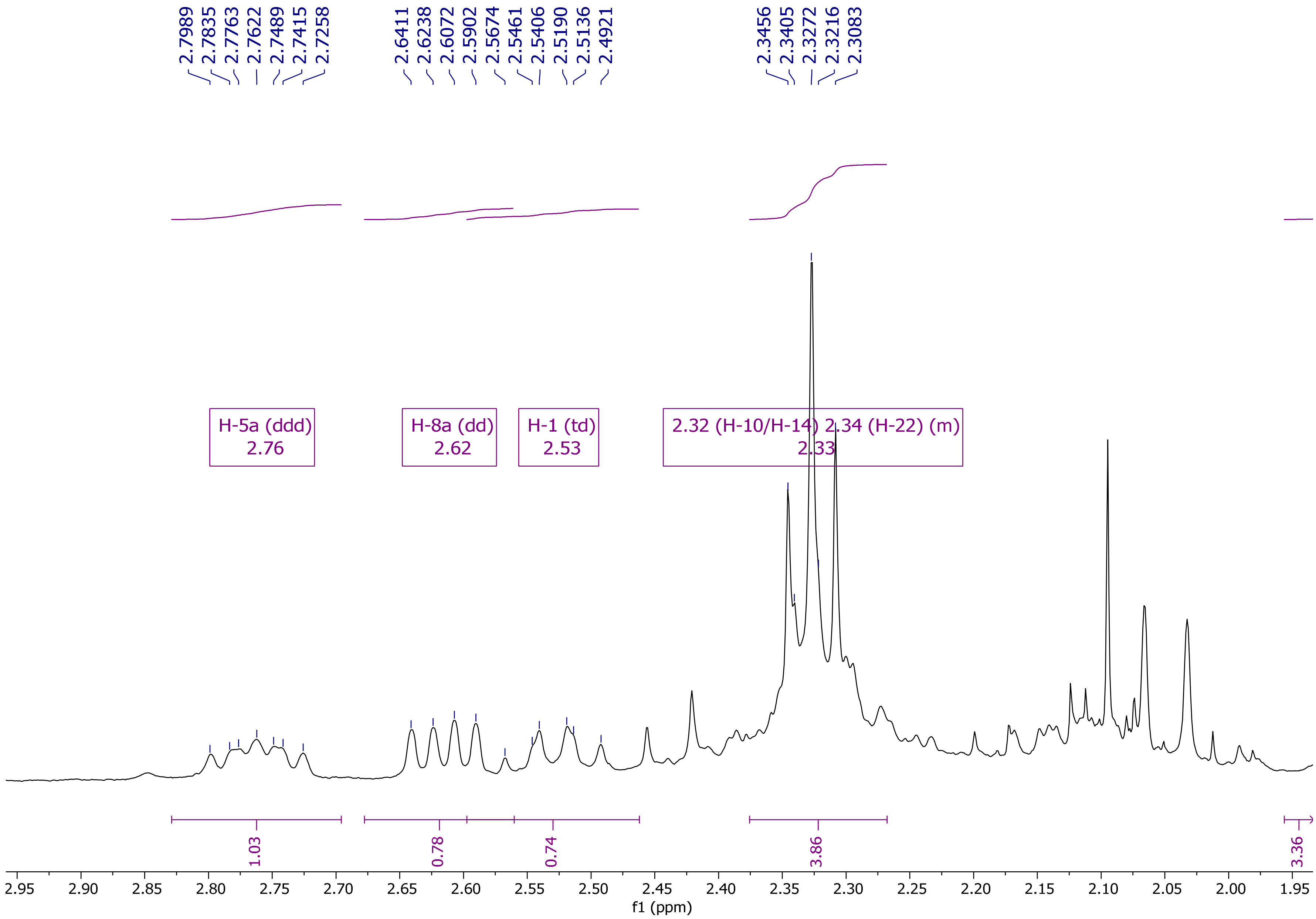

Figure S1. Briarellin T, ^{1}H NMR spectrum (expanded)

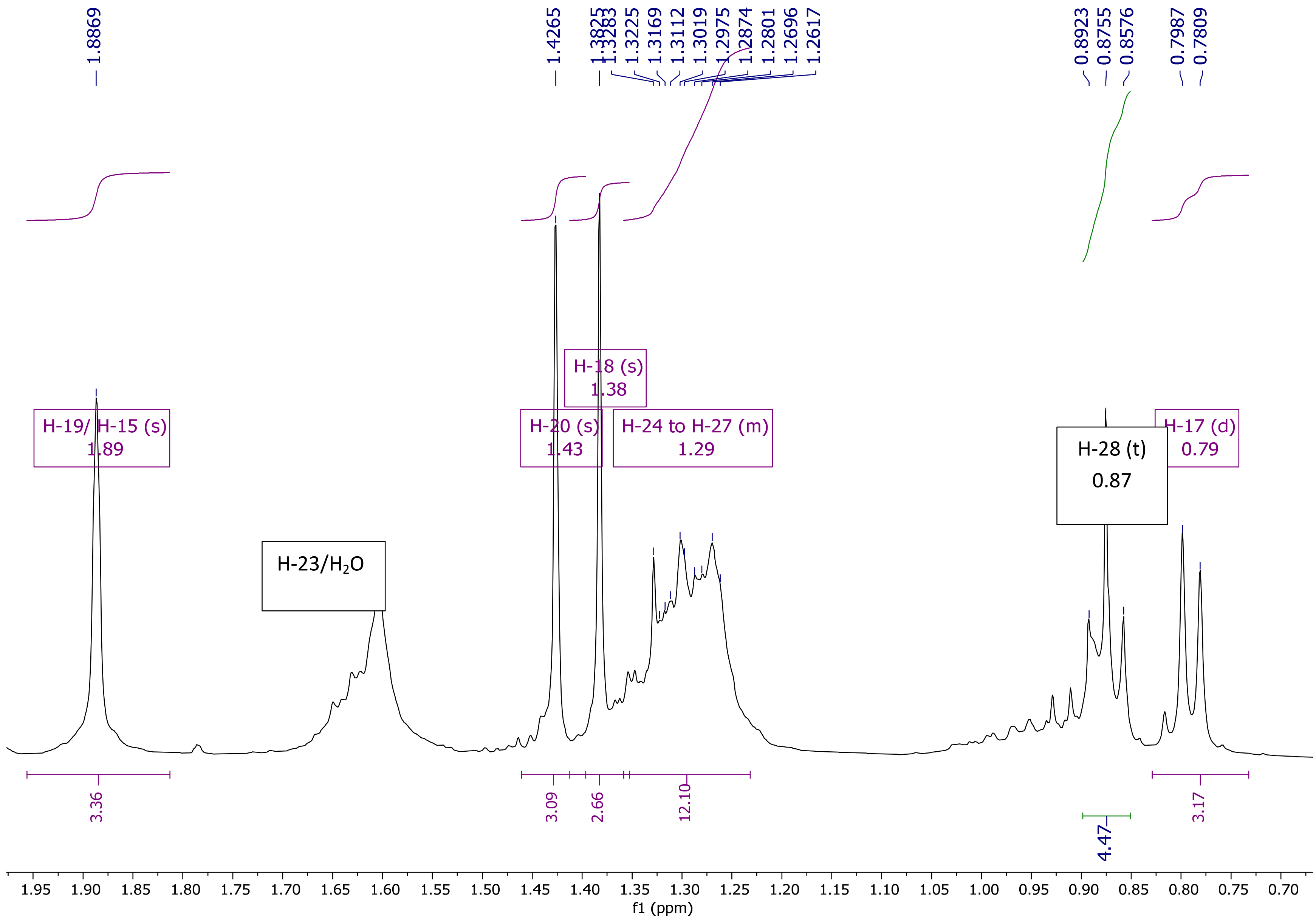

Figure S2. Briarellin T, ^{13}C NMR spectrum

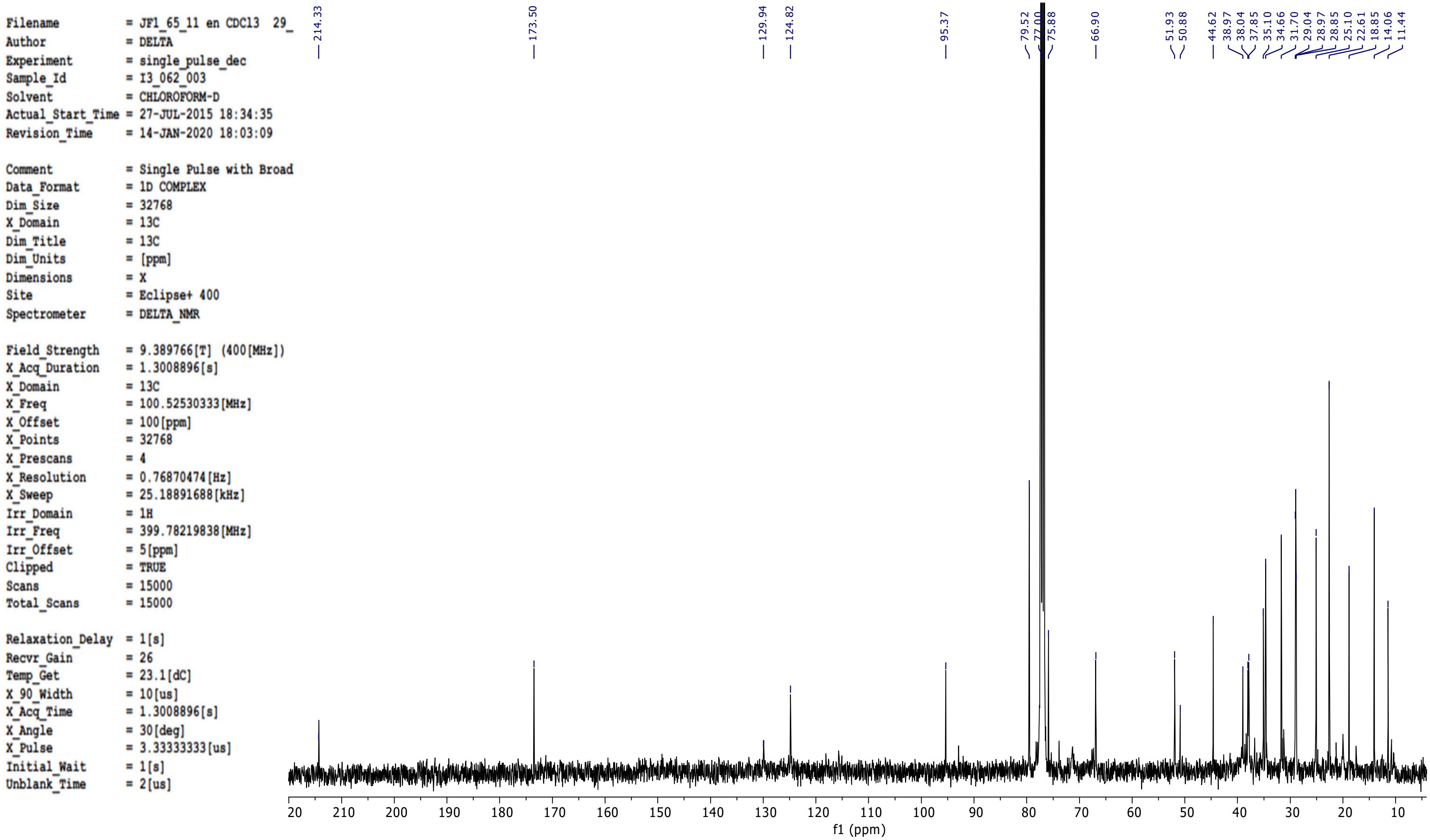

Figure S3. Briarellin T, DEPT-135 spectrum

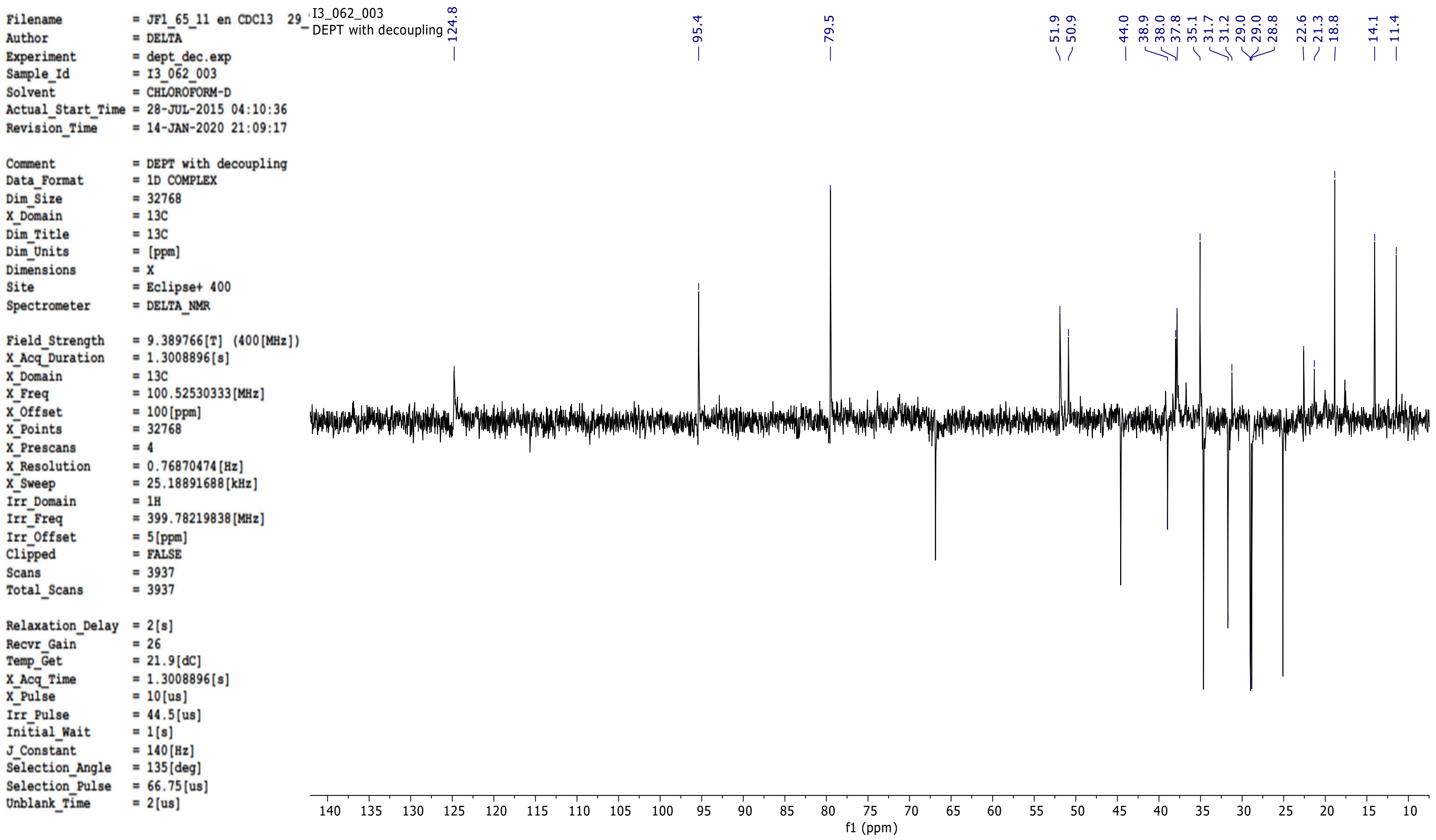

Figure S4. Briarellin T, COSY spectrum

```
Filename           = JF1_65_11_CDCl3  30_7_1
Author             = DELTA
Experiment         = dqf_cosy.exp
Sample_Id          = I3_062_003
Solvent            = CHLOROFORM-D
Actual_Start_Time  = 29-JUL-2015 05:54:39
Revision_Time      = 23-OCT-2018 12:47:02

Comment            = absolute value DQF COSY
Data_Format        = 2D REAL REAL
Dim_Size           = 512, 1024
X_Domain           = 1H
Y_Domain           = 1H
Dim_Title          = 1H 1H
Dim_Units          = [ppm] [ppm]
Dimensions         = X Y
Site               = Eclipse+ 400
Spectrometer       = DELTA_NMR

Field_Strength     = 9.389766[T] (400[MHz])
X_Acq_Duration     = 0.1133568[s]
X_Domain           = 1H
X_Freq             = 399.78219838[MHz]
X_Offset           = 4.68335[ppm]
X_Points           = 512
X_Prescans         = 4
X_Resolution       = 8.8217028[Hz]
X_Sweep            = 4.51671183[kHz]
Y_Domain           = 1H
Y_Freq             = 399.78219838[MHz]
Y_Offset           = 4.68335[ppm]
Y_Points           = 256
Y_Prescans         = 0
Y_Resolution       = 17.6434056[Hz]
Y_Sweep            = 4.51671183[kHz]
Clipped            = FALSE
Scans              = 16
Total_Scans        = 4096

Relaxation_Delay   = 1.5[s]
Recvr_Gain         = 15
Temp_Get           = 21.6[dC]
X_Acq_Time         = 0.1133568[s]
X_Pulse            = 9.81[us]
Y_Acq_Time         = 56.6784[ms]
Initial_Wait       = 1[s]
T1                 = 1[us]
Unblank_Time       = 2[us]
```

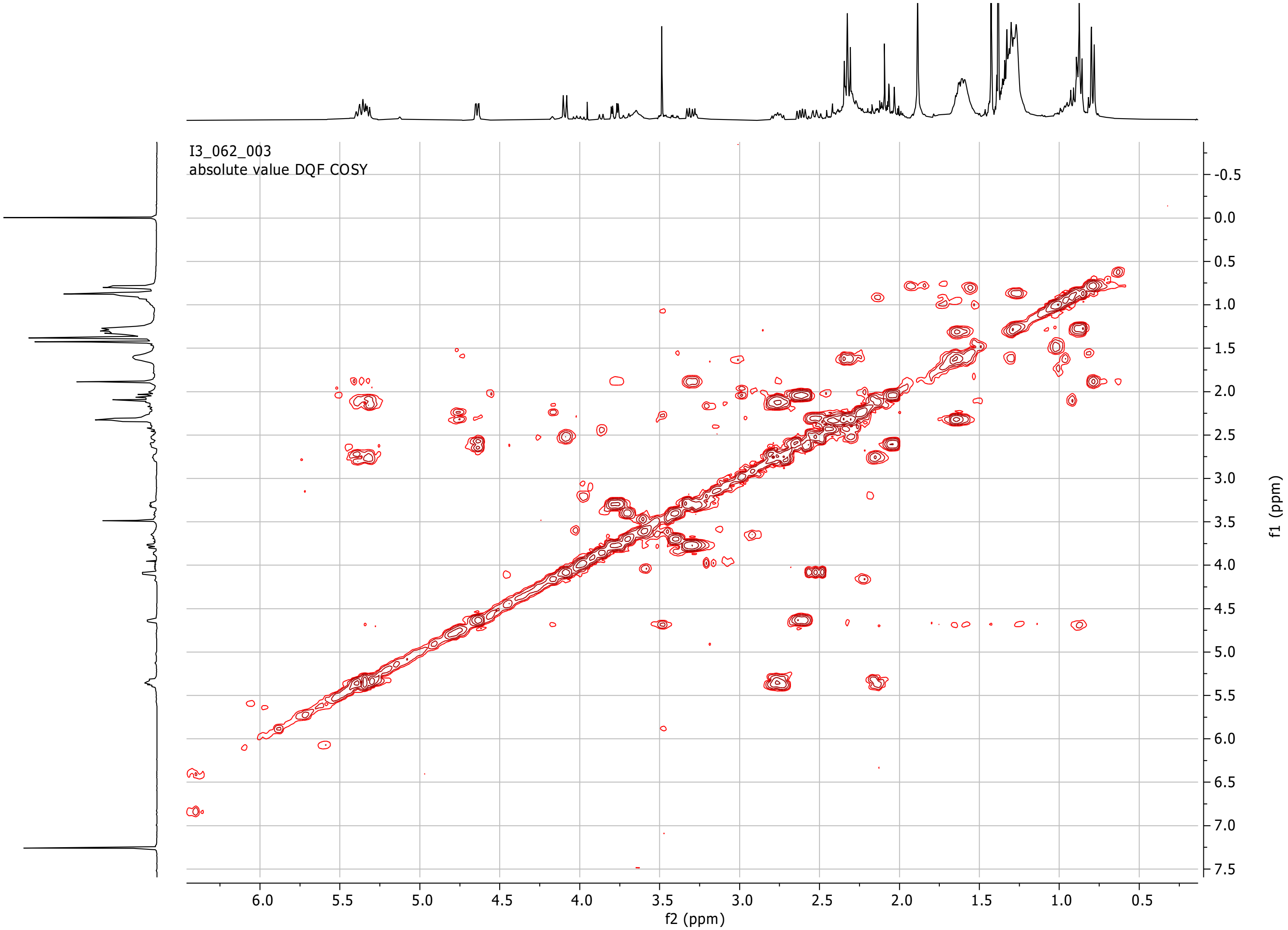

Figure S5. Briarellin T, HSQC spectrum

```
Filename             = JF1_65_11_CDCl3   29_7_1!
Author               = DELTA
Experiment           = multiplicity_hsq
Sample_Id            = I3_062_003
Solvent              = CHLOROFORM-D
Actual_Start_Time    = 28-JUL-2015 10:56:48
Revision_Time        = 14-JAN-2020 21:23:24

Comment              = Multiplicity HSQC
Data_Format          = 2D COMPLEX COMPLEX
Dim_Size             = 2048, 324
X_Domain             = 13C
Y_Domain             = 13C
Dim_Title            = 13C 13C
Dim_Units            = [ppm] [ppm]
Dimensions           = X Y
Site                 = Eclipse+ 400
Spectrometer         = DELTA_NMR

Field_Strength       = 9.389766[T] (400[MHz])
X_Acq_Duration       = 66.9696[ms]
X_Domain             = 13C
X_Freq               = 100.52530333[MHz]
X_Offset             = 68.30581[ppm]
X_Points             = 1024
X_Prescans           = 4
X_Resolution         = 14.93214832[Hz]
X_Sweep              = 15.29051988[kHz]
Y_Domain             = 13C
Y_Freq               = 100.52530333[MHz]
Y_Offset             = 100[ppm]
Y_Points             = 162
Y_Prescans           = 0
Y_Resolution         = 155.87978551[Hz]
Y_Sweep              = 25.25252525[kHz]
Clipped              = FALSE
Scans                = 16
Total_Scans          = 2579

Relaxation_Delay     = 2[s]
Recvr_Gain           = 27
Temp_Get             = 22.1[dC]
X_Acq_Time           = 66.9696[ms]
X_Pulse              = 10[us]
Y_Acq_Time           = 10.1376[ms]
Y_Pulse              = 10[us]
Enhance_Temp         = 12
Enhancement          = 1/6J
Grad_1               = 1[ms]
Grad_1_Amp           = 4[pnt]
Grad_1_Value         = 4[pnt*ms]
Grad_2               = 1[ms]
Grad_2_Amp           = -1[pnt]
Grad_2_Value         = -1[pnt*ms]
Grad_3               = 1[ms]
Grad_3_Amp           = 1[pnt]
Grad_Recover         = 0.1[ms]
Grad_Selection       = 13C = 4:1
Grad_Shape           = square
Grad_Type            = 0
Initial_Wait         = 1[s]
J_Constant           = 140[Hz]
T1                   = 1[us]
Tau                  = 0.5952381[ms]
```

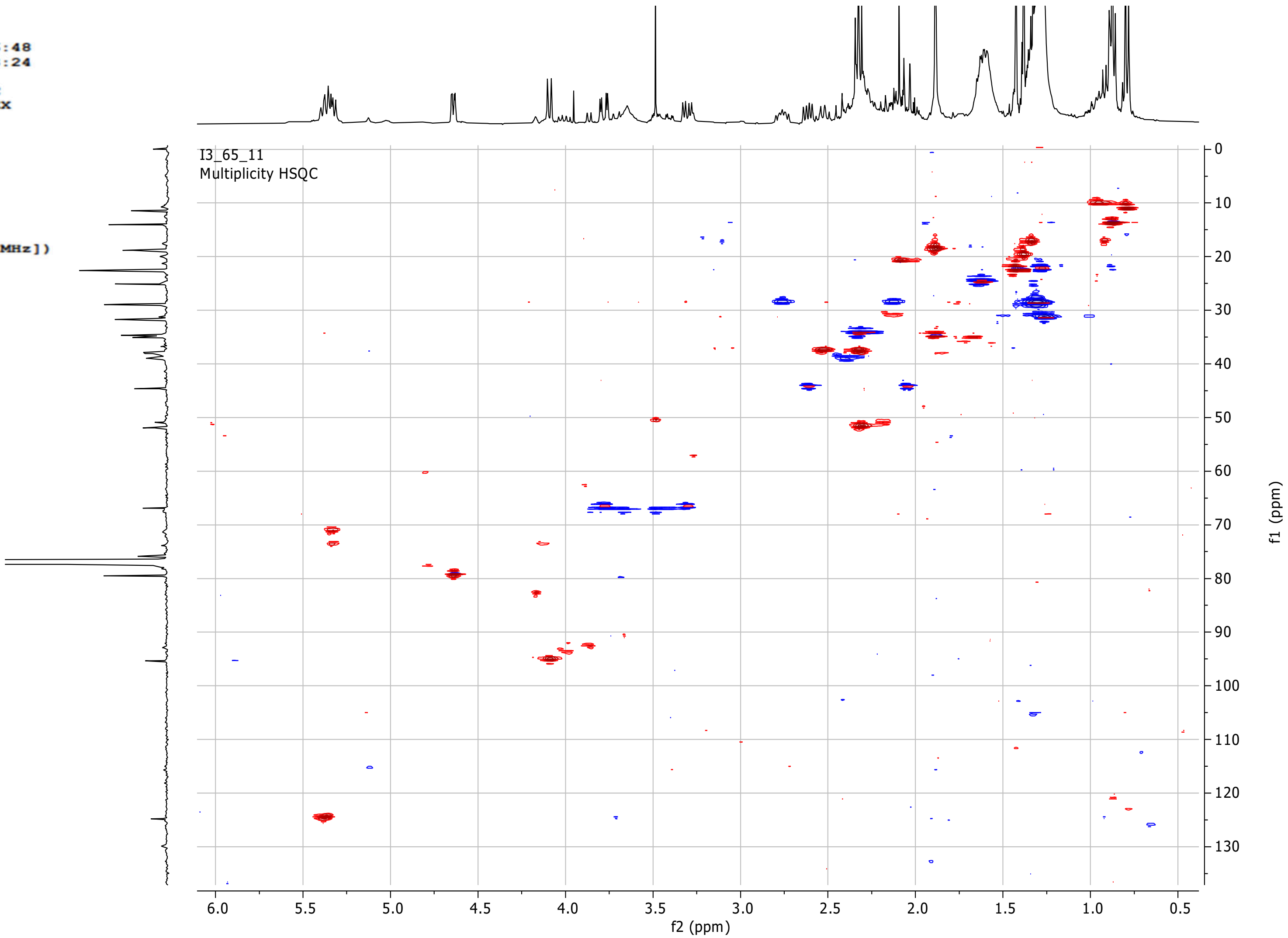

Figure S6. Briarellin T, HMBC spectrum

```
Filename            = JF1_65_11_CDCl3   30_7_1[illegible]
Author              = DELTA
Experiment          = hmbc_pfg_s.exp
Sample_Id           = I3_062_003
Solvent             = CHLOROFORM-D
Actual_Start_Time   = 29-JUL-2015 00:40:13
Revision_Time       = 31-JUL-2015 07:55:57

Comment             = gradient enhanced HMBC
Data_Format         = 2D REAL REAL
Dim_Size            = 1024, 512
X_Domain            = 1H
Y_Domain            = 13C
Dim_Title           = 1H 13C
Dim_Units           = [ppm] [ppm]
Dimensions          = X Y
Site                = Eclipse+ 400
Spectrometer        = DELTA_NMR

Field_Strength      = 9.389766[T] (400[MHz])
X_Acq_Duration      = 0.2267136[s]
X_Domain            = 1H
X_Freq              = 399.78219838[MHz]
X_Offset            = 4.68335[ppm]
X_Points            = 1024
X_Prescans          = 4
X_Resolution        = 4.4108514[Hz]
X_Sweep             = 4.51671183[kHz]
Y_Domain            = 13C
Y_Freq              = 100.52530333[MHz]
Y_Offset            = 100[ppm]
Y_Points            = 256
Y_Prescans          = 0
Y_Resolution        = 98.64267677[Hz]
Y_Sweep             = 25.25252525[kHz]
Clipped             = FALSE
Scans               = 32
Total_Scans         = 8192

Relaxation_Delay    = 2[s]
Recvr_Gain          = 30
Temp_Get            = 21.3[dC]
X_Acq_Time          = 0.2267136[s]
X_Pulse             = 9.81[us]
Y_Acq_Time          = 10.1376[ms]
Y_Pulse             = 12[us]
Grad_1              = 1[ms]
Grad_1_Amp          = 10[pnt]
Grad_1_Value        = 10[pnt*ms]
Grad_2              = 1[ms]
Grad_2_Amp          = 10[pnt]
Grad_2_Value        = 10[pnt*ms]
Grad_3              = 1[ms]
Grad_3_Amp          = 5[pnt]
Grad_3_Value        = 5[pnt*ms]
Grad_Recover        = 0.2[ms]
Grad_Selection      = 13C = 2:2:1
Grad_Shape          = square
Grad_Type           = 0
Initial_Wait        = 1[s]
J_Constant          = 140[Hz]
Long_Range_J        = 8[Hz]
T1                  = 1[us]
Unblank_Time        = 2[us]
```

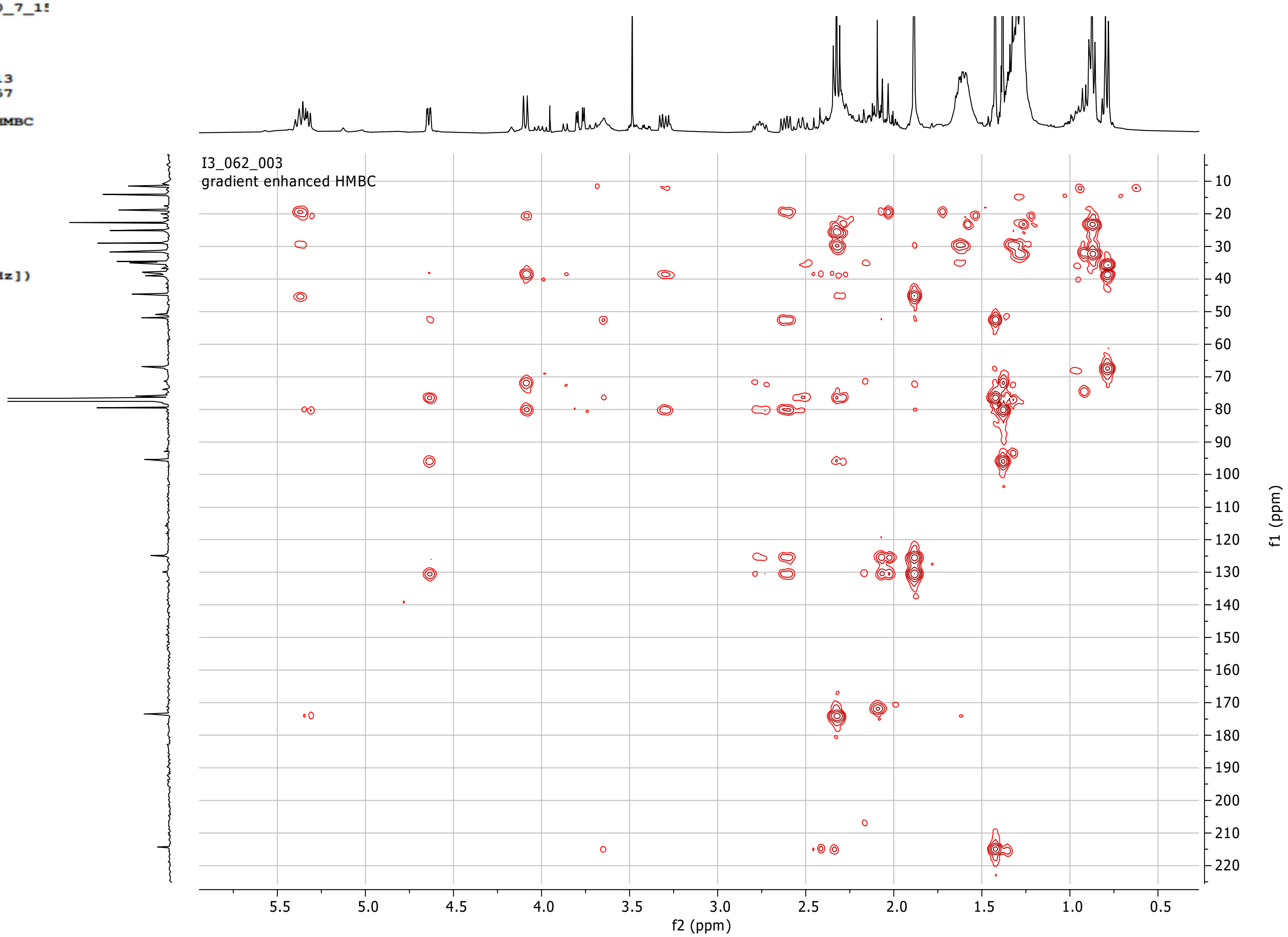

Figure S7. Briarellin T, NOE spectra

Irradiation of H-17

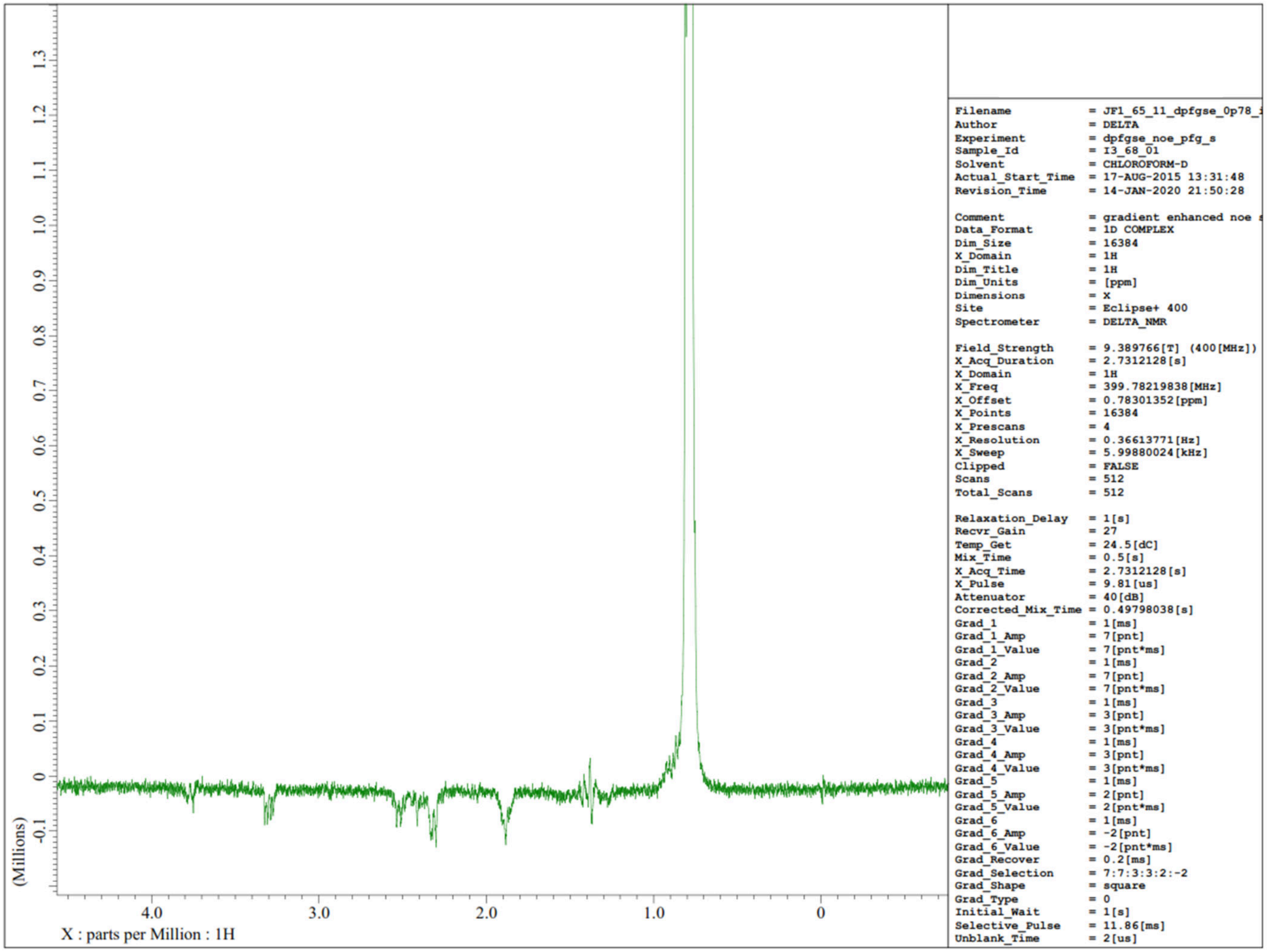

Figure S7. Briarellin T, NOE spectra

Irradiation of H-18

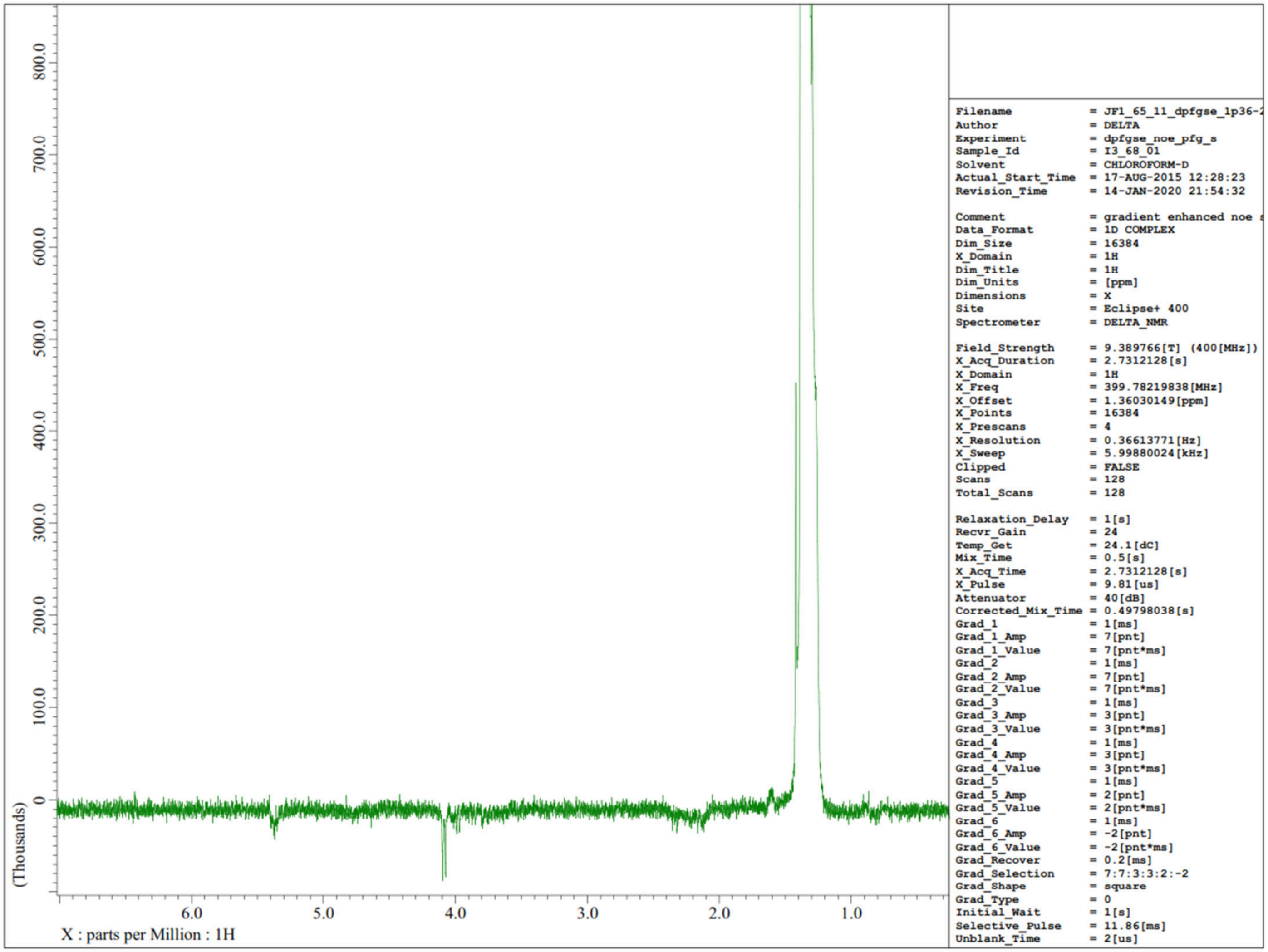

Figure S7. Briarellin T, NOE spectra

Irradiation of H-20

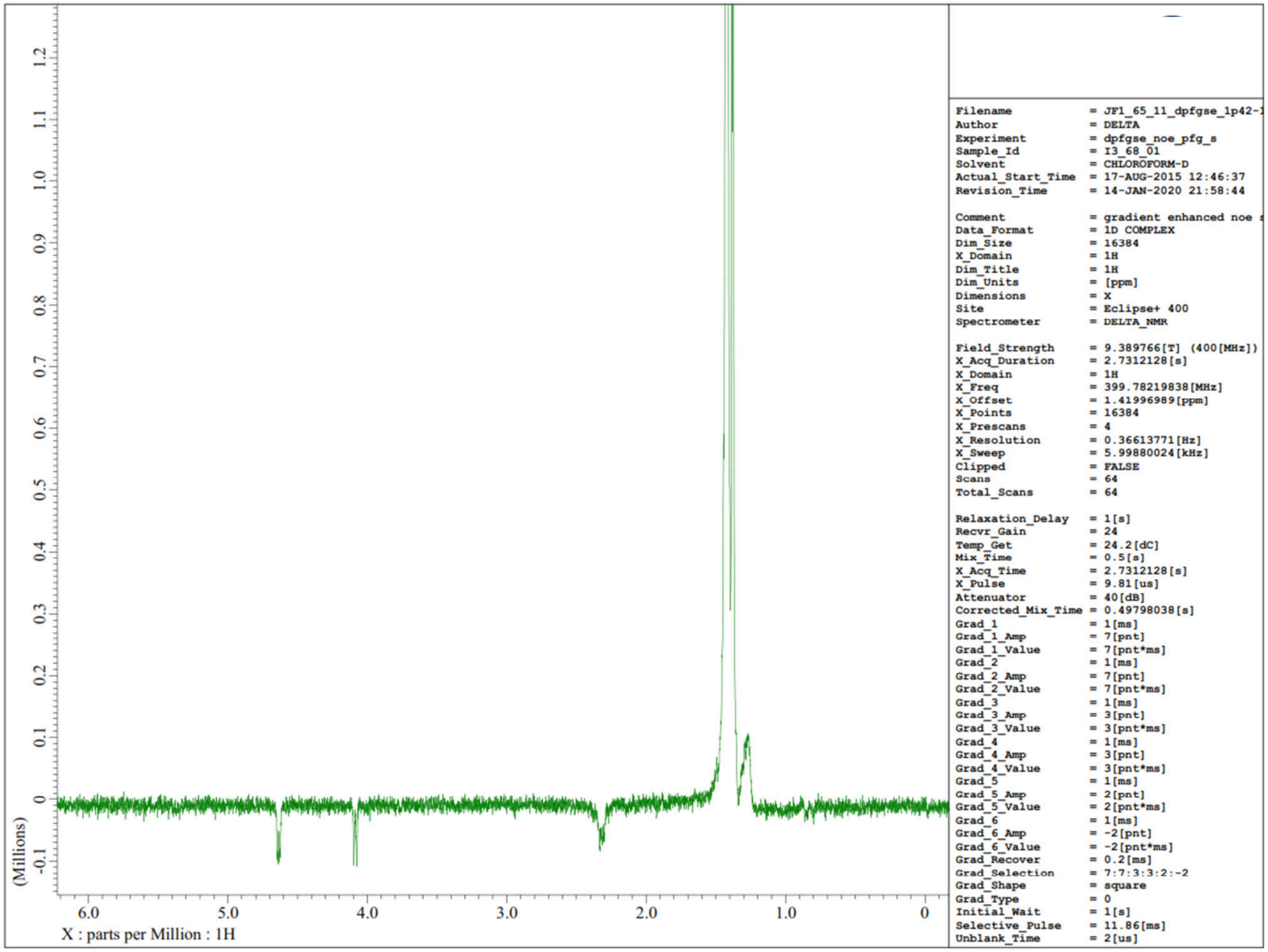

Figure S8. Briarellin T, HR-ESITOFMS spectrum

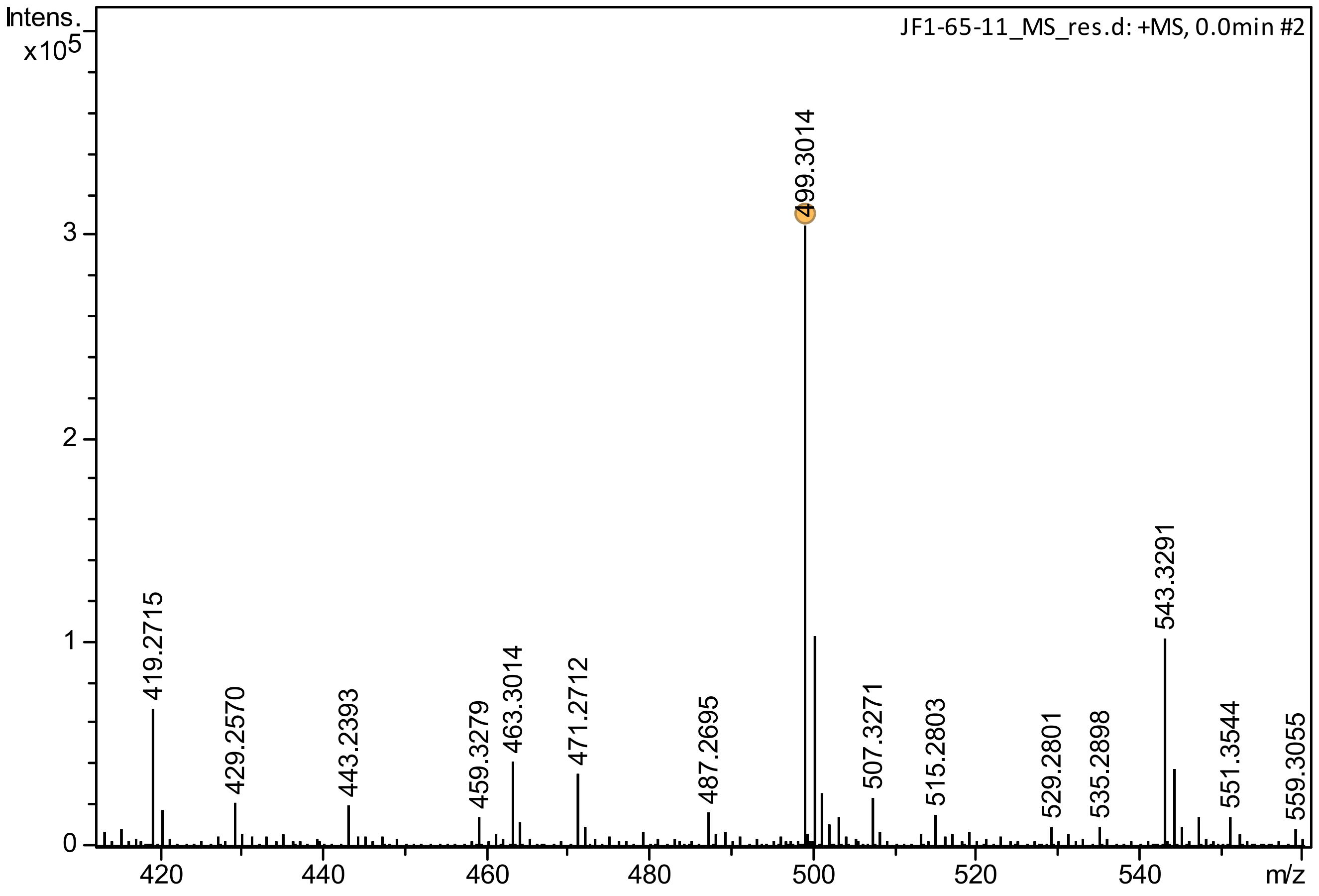

Figure S9. Asbestinin 27, ^{1}H NMR spectrum

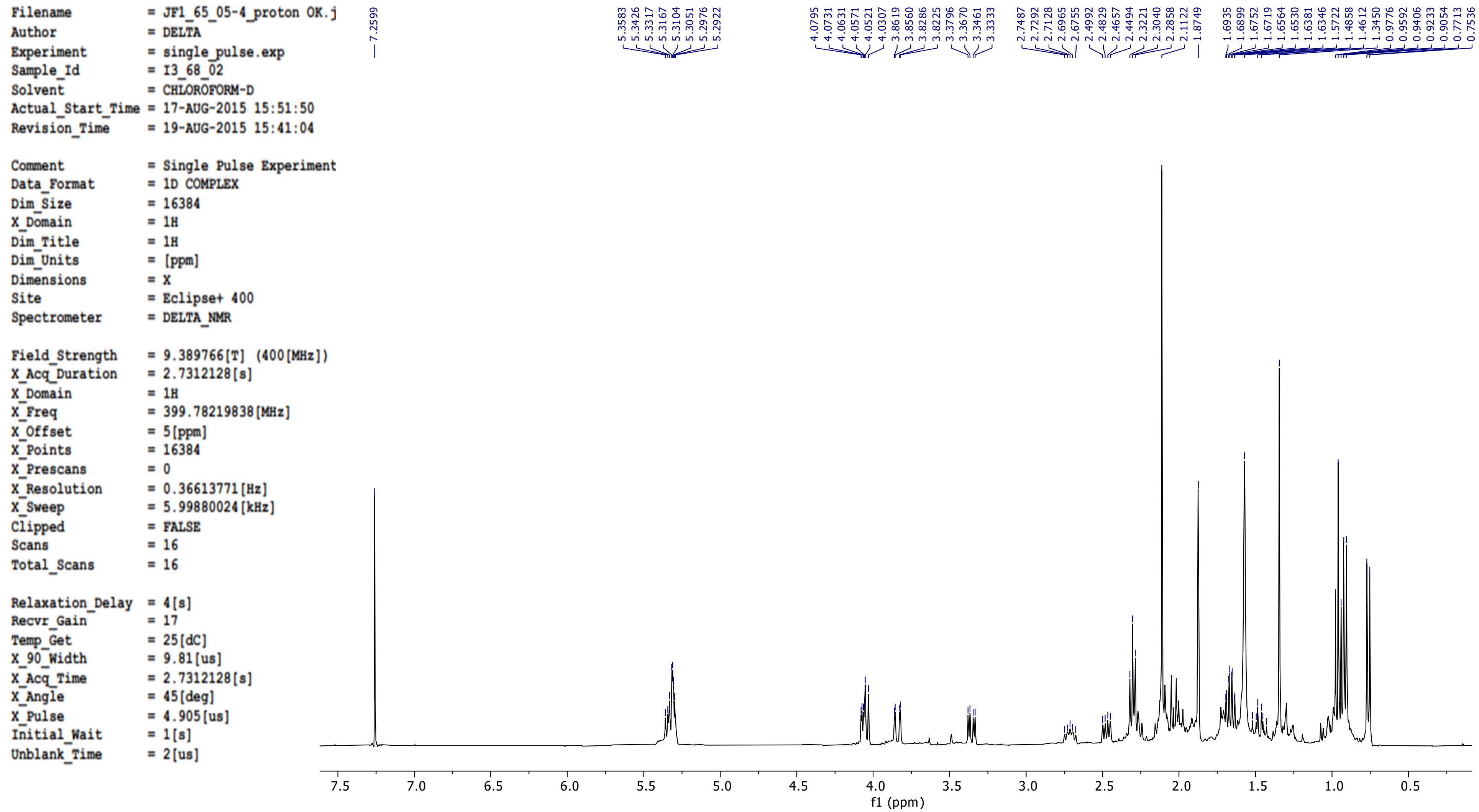

Figure S9. Asbestinin 27, ^{1}H NMR spectrum (expanded)

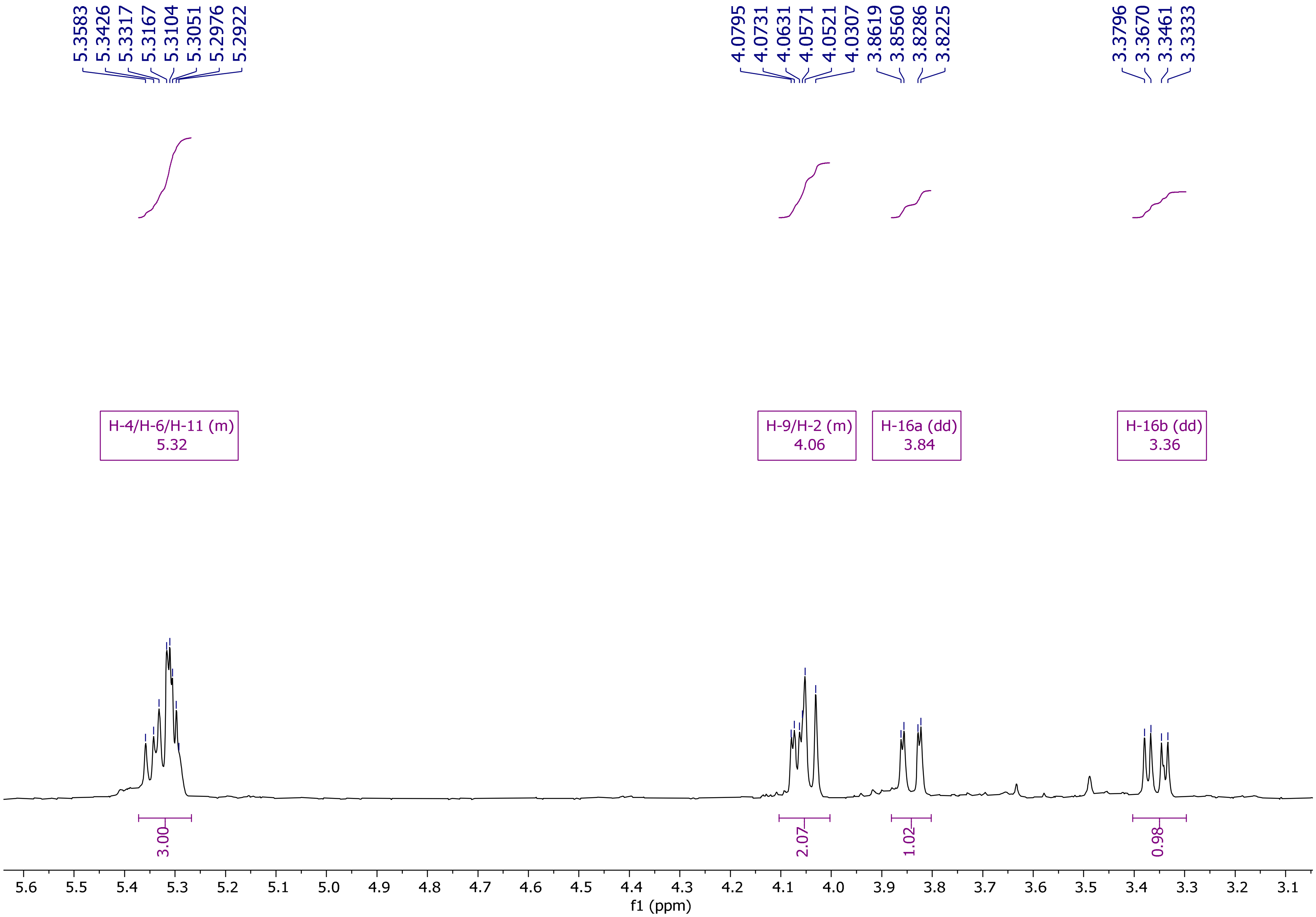

Figure S9. Asbestinin 27, ¹H NMR spectrum (expanded)

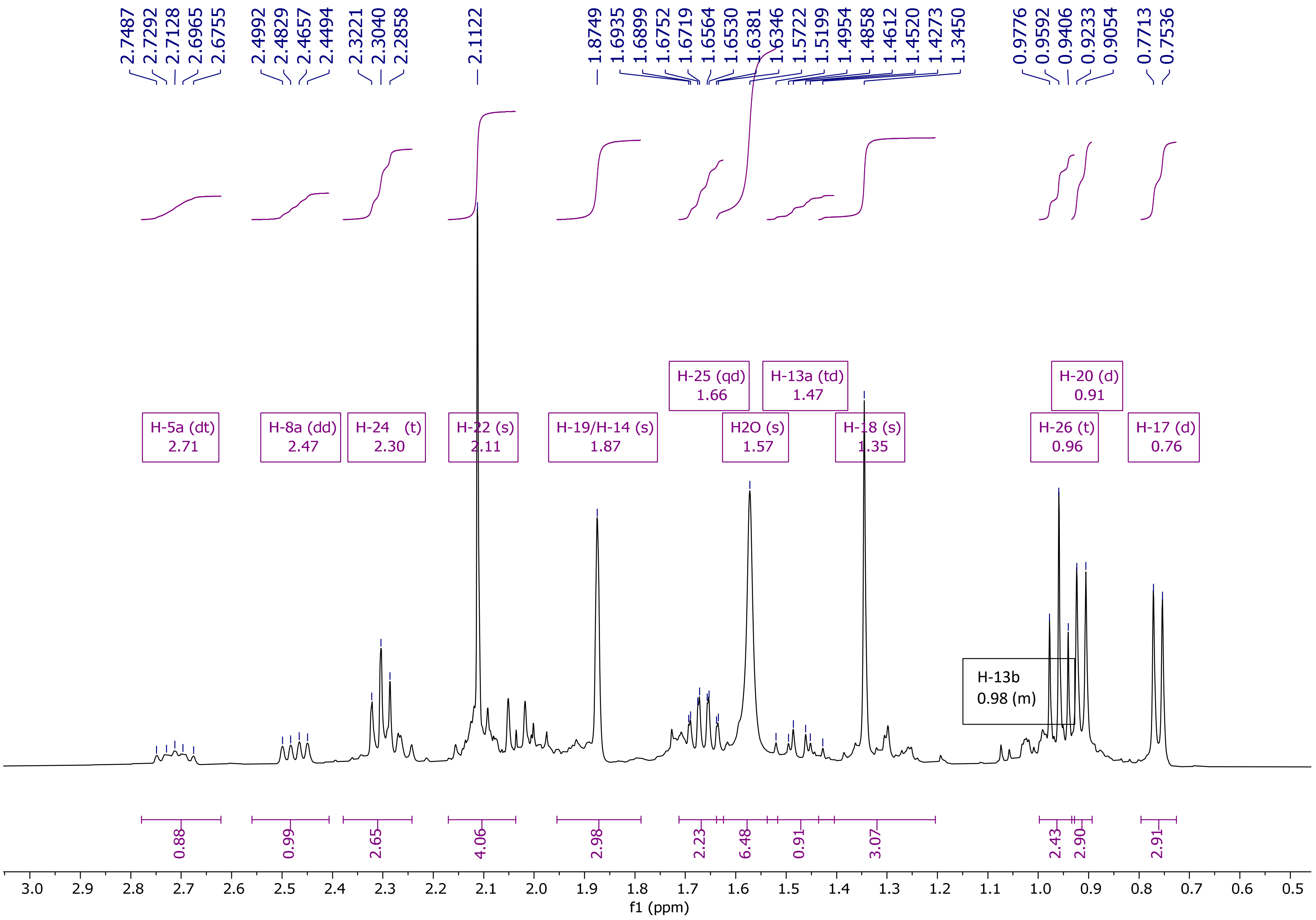

Figure S10. Asbestinin 27, ^{13}C NMR spectrum

Filename	= JF1_65_05_C13-2.jdf
Author	= DELTA
Experiment	= single_pulse_dec
Sample_Id	= I3_64_01
Solvent	= CHLOROFORM-D
Actual_Start_Time	= 5-AUG-2015 16:50:00
Revision_Time	= 8-AUG-2015 03:05:23
Comment	= Single Pulse with Broad
Data_Format	= 1D COMPLEX
Dim_Size	= 32768
X_Domain	= 13C
Dim_Title	= 13C
Dim_Units	= [ppm]
Dimensions	= X
Site	= Eclipse+ 400
Spectrometer	= DELTA_NMR
Field_Strength	= 9.389766[T] (400[MHz])
X_Acq_Duration	= 1.3008896[s]
X_Domain	= 13C
X_Freq	= 100.52530333[MHz]
X_Offset	= 100[ppm]
X_Points	= 32768
X_Prescans	= 4
X_Resolution	= 0.76870474[Hz]
X_Sweep	= 25.18891688[kHz]
Irr_Domain	= 1H
Irr_Freq	= 399.78219838[MHz]
Irr_Offset	= 5[ppm]
Clipped	= FALSE
Scans	= 16384
Total_Scans	= 16384
Relaxation_Delay	= 1[s]
Recvr_Gain	= 27
Temp_Get	= 26.8[dC]
X_90_Width	= 10[us]
X_Acq_Time	= 1.3008896[s]
X_Angle	= 30[deg]
X_Pulse	= 3.33333333[us]
Initial_Wait	= 1[s]
Unblank_Time	= 2[us]

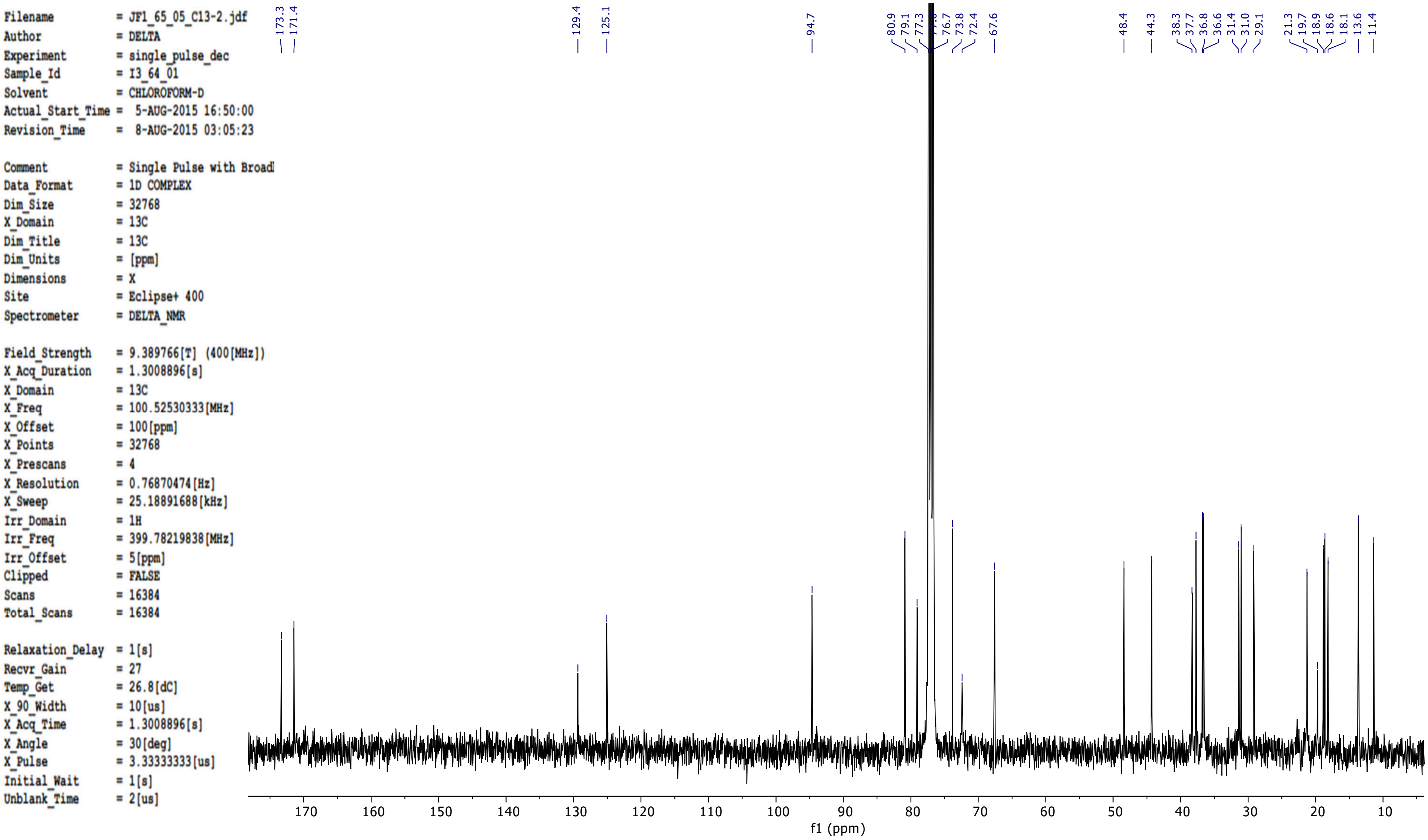

Figure S11. Asbestinin 27, DEPT-135 spectrum

```
Filename           = JF1_65_05_dept135-2.jdf
Author             = DELTA
Experiment         = dept_dec.exp
Sample_Id          = I3_68_02
Solvent            = CHLOROFORM-D
Actual_Start_Time  = 17-AUG-2015 15:56:24
Revision_Time      = 14-JAN-2020 22:39:27

Comment            = DEPT with decoupling
Data_Format        = 1D COMPLEX
Dim_Size           = 32768
X_Domain           = 13C
Dim_Title          = 13C
Dim_Units          = [ppm]
Dimensions         = X
Site               = Eclipse+ 400
Spectrometer       = DELTA_NMR

Field_Strength     = 9.389766[T] (400[MHz])
X_Acq_Duration     = 1.3008896[s]
X_Domain           = 13C
X_Freq             = 100.52530333[MHz]
X_Offset           = 100[ppm]
X_Points           = 32768
X_Prescans         = 4
X_Resolution       = 0.76870474[Hz]
X_Sweep            = 25.18891688[kHz]
Irr_Domain         = 1H
Irr_Freq           = 399.78219838[MHz]
Irr_Offset         = 5[ppm]
Clipped            = FALSE
Scans              = 1024
Total_Scans        = 1024

Relaxation_Delay   = 2[s]
Recvr_Gain         = 27
Temp_Get           = 25.7[dC]
X_Acq_Time         = 1.3008896[s]
X_Pulse            = 10[us]
Irr_Pulse          = 44.5[us]
Initial_Wait       = 1[s]
J_Constant         = 140[Hz]
Selection_Angle    = 135[deg]
Selection_Pulse    = 66.75[us]
Unblank_Time       = 2[us]
```

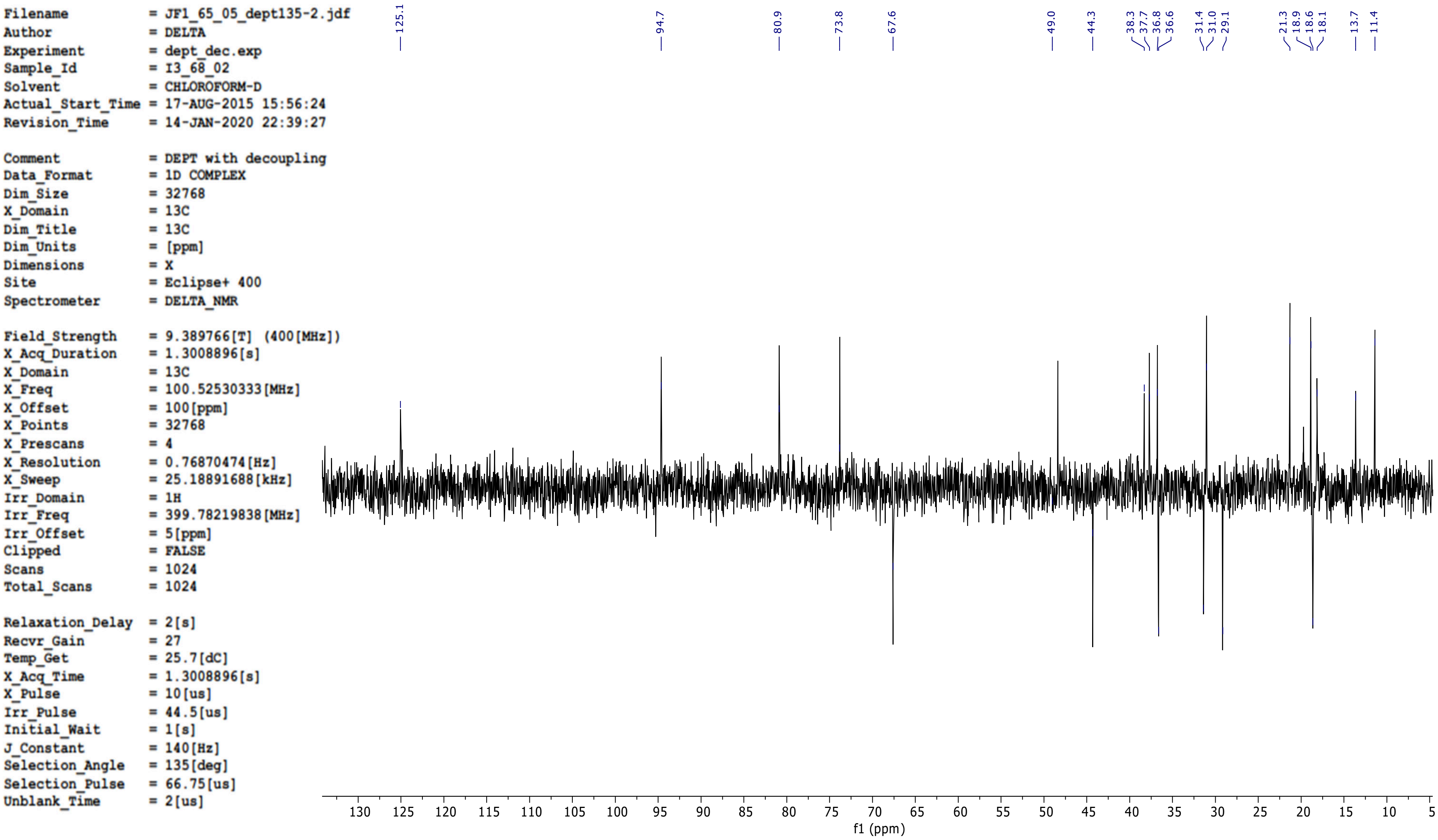

Figure S12. Asbestinin 27, COSY spectrum

```
Filename            = JF1_65_05_cosy-2.jdf
Author              = DELTA
Experiment          = cosy_pfg_s.exp
Sample_Id           = I3_68_02
Solvent             = CHLOROFORM-D
Actual_Start_Time   = 18-AUG-2015 04:45:39
Revision_Time       = 14-JAN-2020 22:44:12

Comment             = gradient absolute value
Data_Format         = 2D REAL REAL
Dim_Size            = 1024, 1024
X_Domain            = 1H
Y_Domain            = 1H
Dim_Title           = 1H 1H
Dim_Units           = [ppm] [ppm]
Dimensions          = X Y
Site                = Eclipse+ 400
Spectrometer        = DELTA_NMR

Field_Strength      = 9.389766[T] (400[MHz])
X_Acq_Duration      = 0.2900992[s]
X_Domain            = 1H
X_Freq              = 399.78219838[MHz]
X_Offset            = 3.62959[ppm]
X_Points            = 1024
X_Prescans          = 4
X_Resolution        = 3.44709672[Hz]
X_Sweep             = 3.52982704[kHz]
Y_Domain            = 1H
Y_Freq              = 399.78219838[MHz]
Y_Offset            = 3.62959[ppm]
Y_Points            = 256
Y_Prescans          = 0
Y_Resolution        = 13.78838687[Hz]
Y_Sweep             = 3.52982704[kHz]
Clipped             = FALSE
Scans               = 8
Total_Scans         = 2048

Relaxation_Delay    = 1[s]
Recvr_Gain          = 18
Temp_Get            = 23.6[dC]
X_90_Width          = 9.81[us]
X_Acq_Time          = 0.2900992[s]
X_Pulse             = 9.81[us]
Y_Acq_Time          = 72.5248[ms]
Grad_1              = 1[ms]
Grad_1_Amp          = 5[pnt]
Grad_1_Value        = 5[pnt*ms]
Grad_2              = 1[ms]
Grad_2_Amp          = 5[pnt]
Grad_2_Value        = 5[pnt*ms]
Grad_Recover        = 1[ms]
Grad_Selection      = 1:1
Grad_Shape          = square
Grad_Type           = 0
Initial_Wait        = 0.1[s]
Pulse_1             = 9.81[us]
Pulse_2             = 9.81[us]
Pulse_Angle_1       = 90[deg]
Pulse_Angle_2       = 90[deg]
T1                  = 1[us]
Unblank_Time        = 2[us]
```

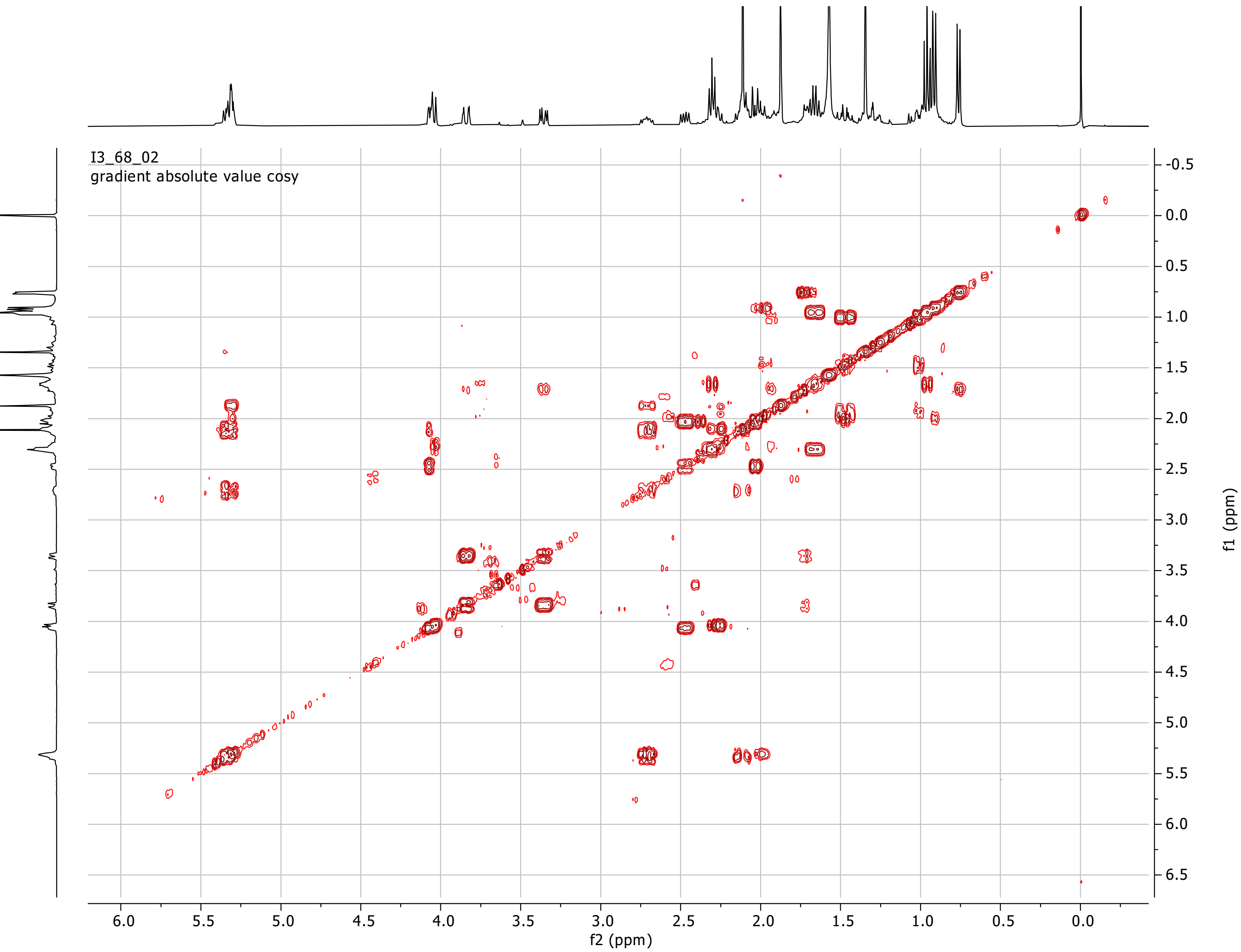

Figure S13. Asbestinin 27, HSQC spectrum

```
Filename            = JF1_65_05_hsqc-2.jdf
Author              = DELTA
Experiment          = multiplicity_hsq
Sample_Id           = I3_68_02
Solvent             = CHLOROFORM-D
Actual_Start_Time   = 17-AUG-2015 17:02:39
Revision_Time       = 14-JAN-2020 22:50:48

Comment             = Multiplicity HSQC
Data_Format         = 2D COMPLEX COMPLEX
Dim_Size            = 2048, 512
X_Domain            = 1H
Y_Domain            = 13C
Dim_Title           = 1H 13C
Dim_Units           = [ppm] [ppm]
Dimensions          = X Y
Site                = Eclipse+ 400
Spectrometer        = DELTA_NMR

Field_Strength      = 9.389766[T] (400[MHz])
X_Acq_Duration      = 0.2900992[s]
X_Domain            = 1H
X_Freq              = 399.78219838[MHz]
X_Offset            = 3.62959[ppm]
X_Points            = 1024
X_Prescans          = 4
X_Resolution        = 3.44709672[Hz]
X_Sweep             = 3.52982704[kHz]
Y_Domain            = 13C
Y_Freq              = 100.52530333[MHz]
Y_Offset            = 100[ppm]
Y_Points            = 256
Y_Prescans          = 0
Y_Resolution        = 98.64267677[Hz]
Y_Sweep             = 25.25252525[kHz]
Clipped             = FALSE
Scans               = 16
Total_Scans         = 4096

Relaxation_Delay    = 2[s]
Recvr_Gain          = 30
Temp_Get            = 24.7[dC]
X_Acq_Time          = 0.2900992[s]
X_Pulse             = 9.81[us]
Y_Acq_Time          = 10.1376[ms]
Y_Pulse             = 12[us]
Enhance_Temp        = 12
Enhancement         = 1/6J
Grad_1              = 1[ms]
Grad_1_Amp          = 4[pnt]
Grad_1_Value        = 4[pnt*ms]
Grad_2              = 1[ms]
Grad_2_Amp          = -1[pnt]
Grad_2_Value        = -1[pnt*ms]
Grad_3              = 1[ms]
Grad_3_Amp          = 1[pnt]
Grad_Recover        = 0.1[ms]
Grad_Selection      = 13C = 4:1
Grad_Shape          = square
Grad_Type           = 0
Initial_Wait        = 1[s]
J_Constant          = 140[Hz]
T1                  = 1[us]
Tau                 = 0.5952381[ms]
```

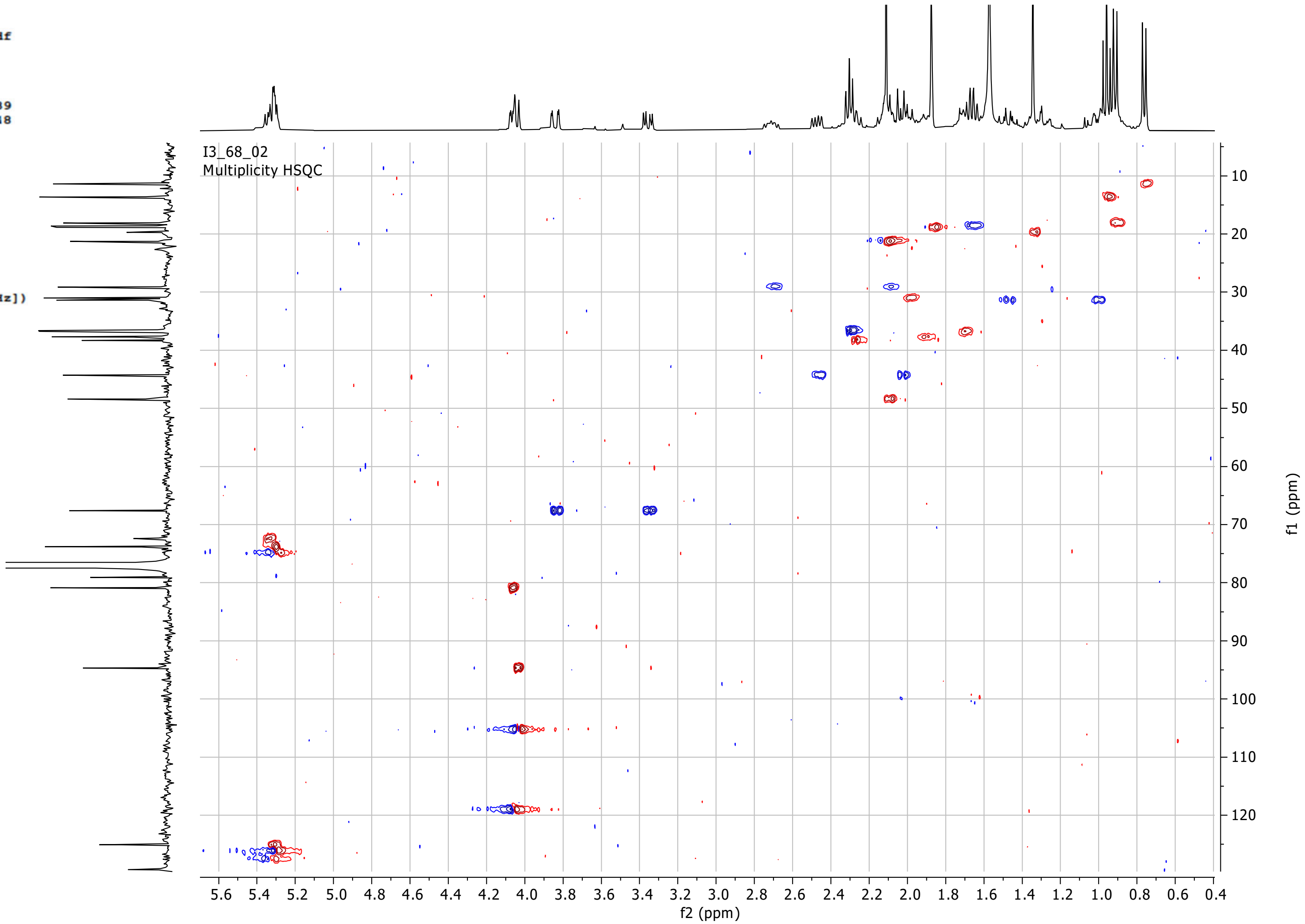

Figure S14. Asbestinin 27, HMBC spectrum

```
Filename            = JF1_65_05_hmbcOKOKOK-2.
Author              = DELTA
Experiment          = hmbc_pfg_s.exp
Sample_Id           = I3_68_02
Solvent             = CHLOROFORM-D
Actual_Start_Time   = 17-AUG-2015 22:19:20
Revision_Time       =  5-JUL-2019 17:15:31

Comment             = gradient enhanced HMBC
Data_Format         = 2D REAL REAL
Dim_Size            = 1024, 512
X_Domain            = 1H
Y_Domain            = 13C
Dim_Title           = 1H 13C
Dim_Units           = [ppm] [ppm]
Dimensions          = X Y
Site                = Eclipse+ 400
Spectrometer        = DELTA_NMR

Field_Strength      = 9.389766[T] (400[MHz])
X_Acq_Duration      = 0.2900992[s]
X_Domain            = 1H
X_Freq              = 399.78219838[MHz]
X_Offset            = 3.62959[ppm]
X_Points            = 1024
X_Prescans          = 4
X_Resolution        = 3.44709672[Hz]
X_Sweep             = 3.52982704[kHz]
Y_Domain            = 13C
Y_Freq              = 100.52530333[MHz]
Y_Offset            = 100[ppm]
Y_Points            = 256
Y_Prescans          = 0
Y_Resolution        = 98.64267677[Hz]
Y_Sweep             = 25.25252525[kHz]
Clipped             = FALSE
Scans               = 32
Total_Scans         = 8192

Relaxation_Delay    = 2[s]
Recvr_Gain          = 30
Temp_Get            = 23.9[dC]
X_Acq_Time          = 0.2900992[s]
X_Pulse             = 9.81[us]
Y_Acq_Time          = 10.1376[ms]
Y_Pulse             = 12[us]
Grad_1              = 1[ms]
Grad_1_Amp          = 10[pnt]
Grad_1_Value        = 10[pnt*ms]
Grad_2              = 1[ms]
Grad_2_Amp          = 10[pnt]
Grad_2_Value        = 10[pnt*ms]
Grad_3              = 1[ms]
Grad_3_Amp          = 5[pnt]
Grad_3_Value        = 5[pnt*ms]
Grad_Recover        = 0.2[ms]
Grad_Selection      = 13C = 2:2:1
Grad_Shape          = square
Grad_Type           = 0
Initial_Wait        = 1[s]
J_Constant          = 140[Hz]
Long_Range_J        = 8[Hz]
T1                  = 1[us]
Unblank_Time        = 2[us]
```

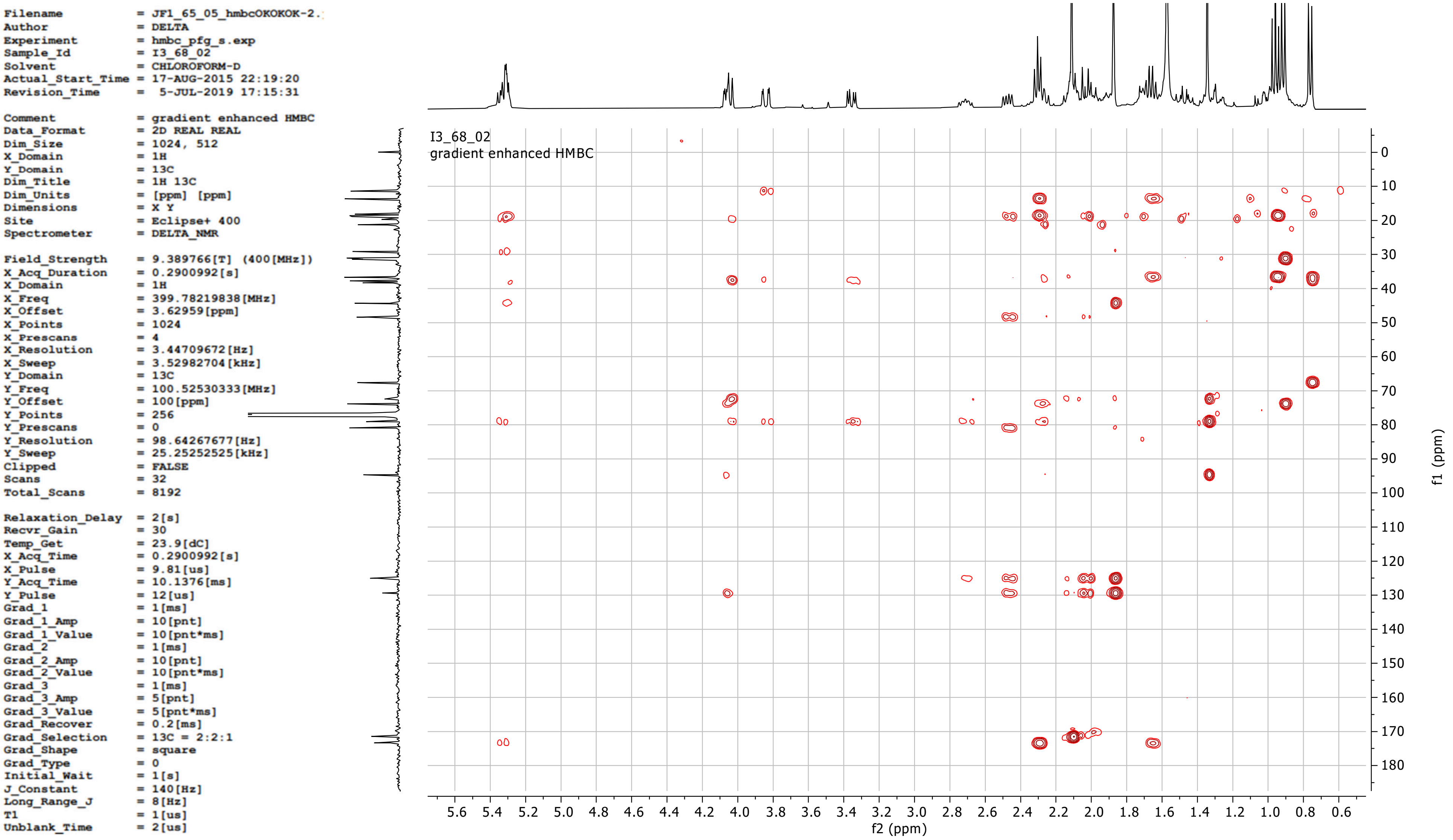

Figure S15. Asbestinin 27, NOE spectra

Irradiation of H-17

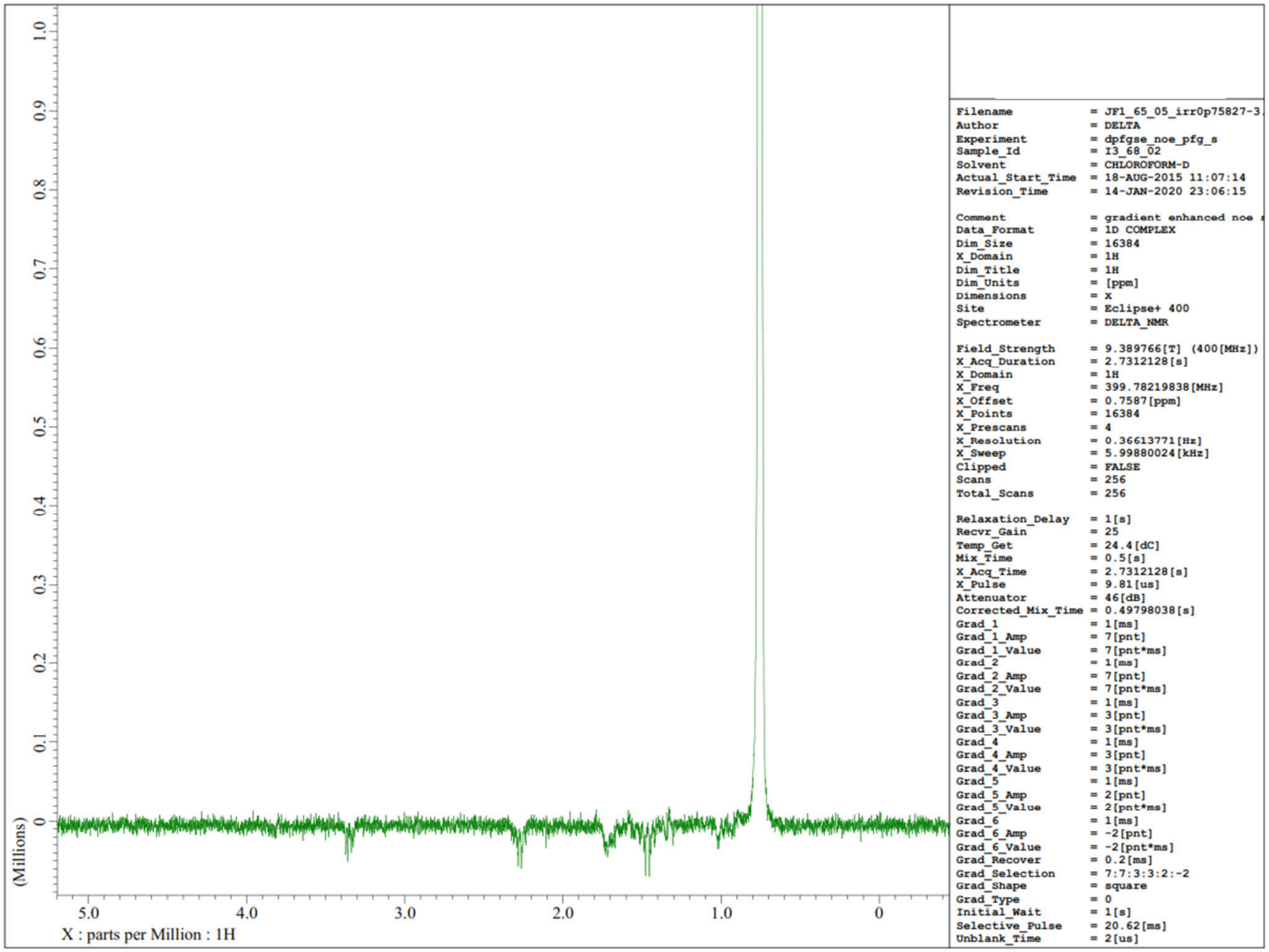

Figure S15. Asbestinin 27, NOE spectra

Irradiation of H-2

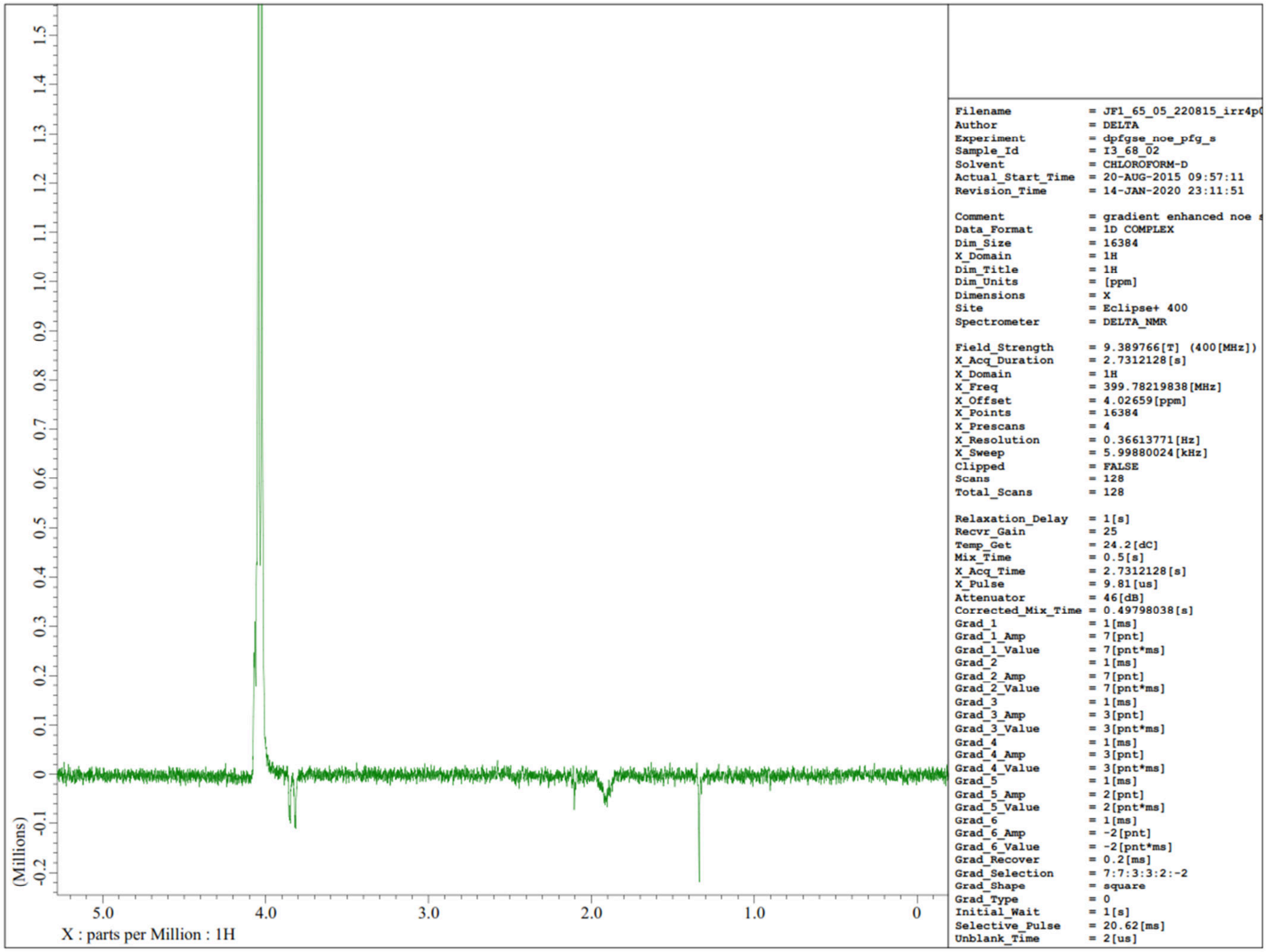

Figure S15. Asbestinin 27, NOE spectra

Irradiation of H-18

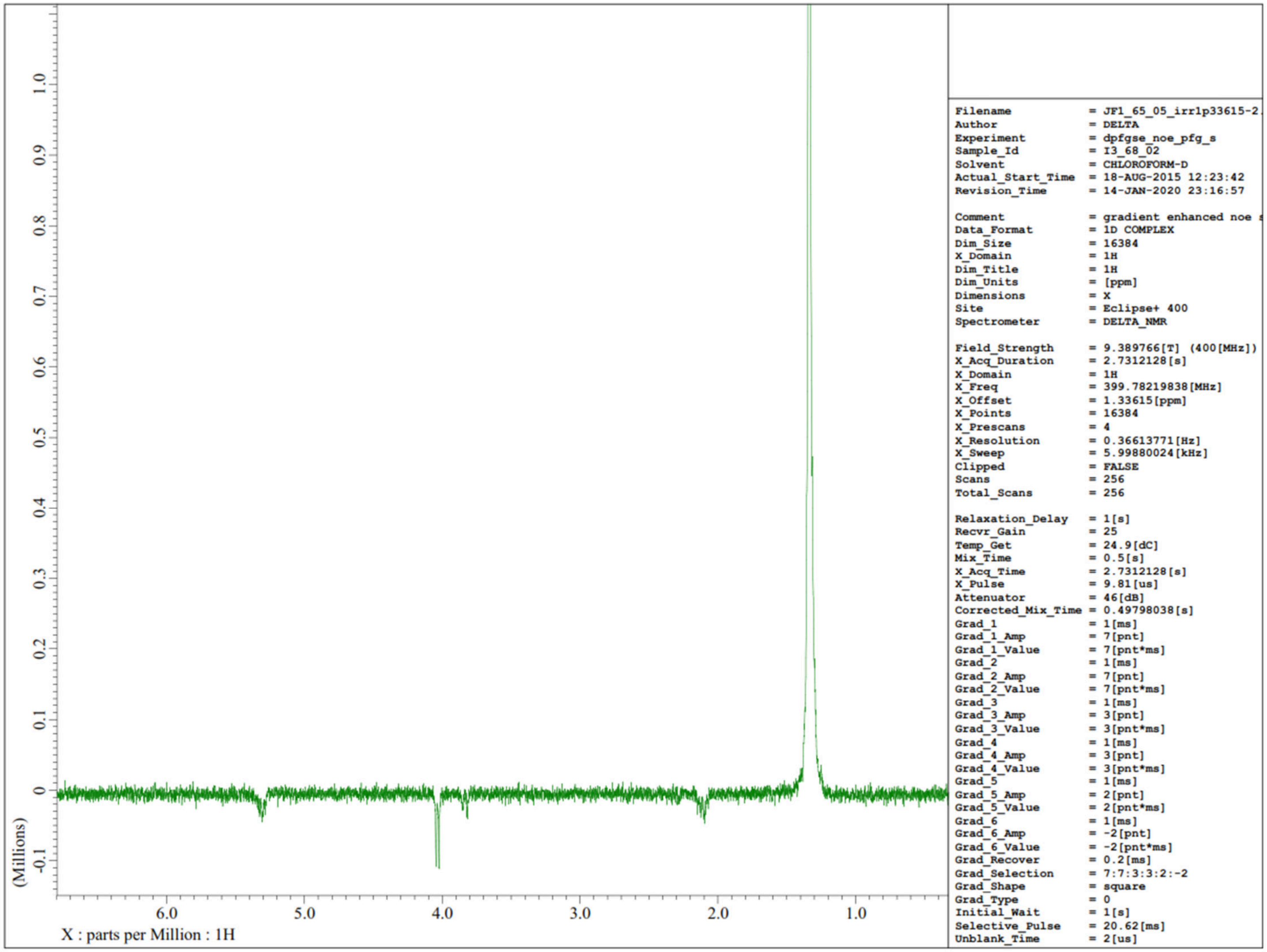

Figure S15. Asbestinin 27, NOE spectra

Irradiation of H-10

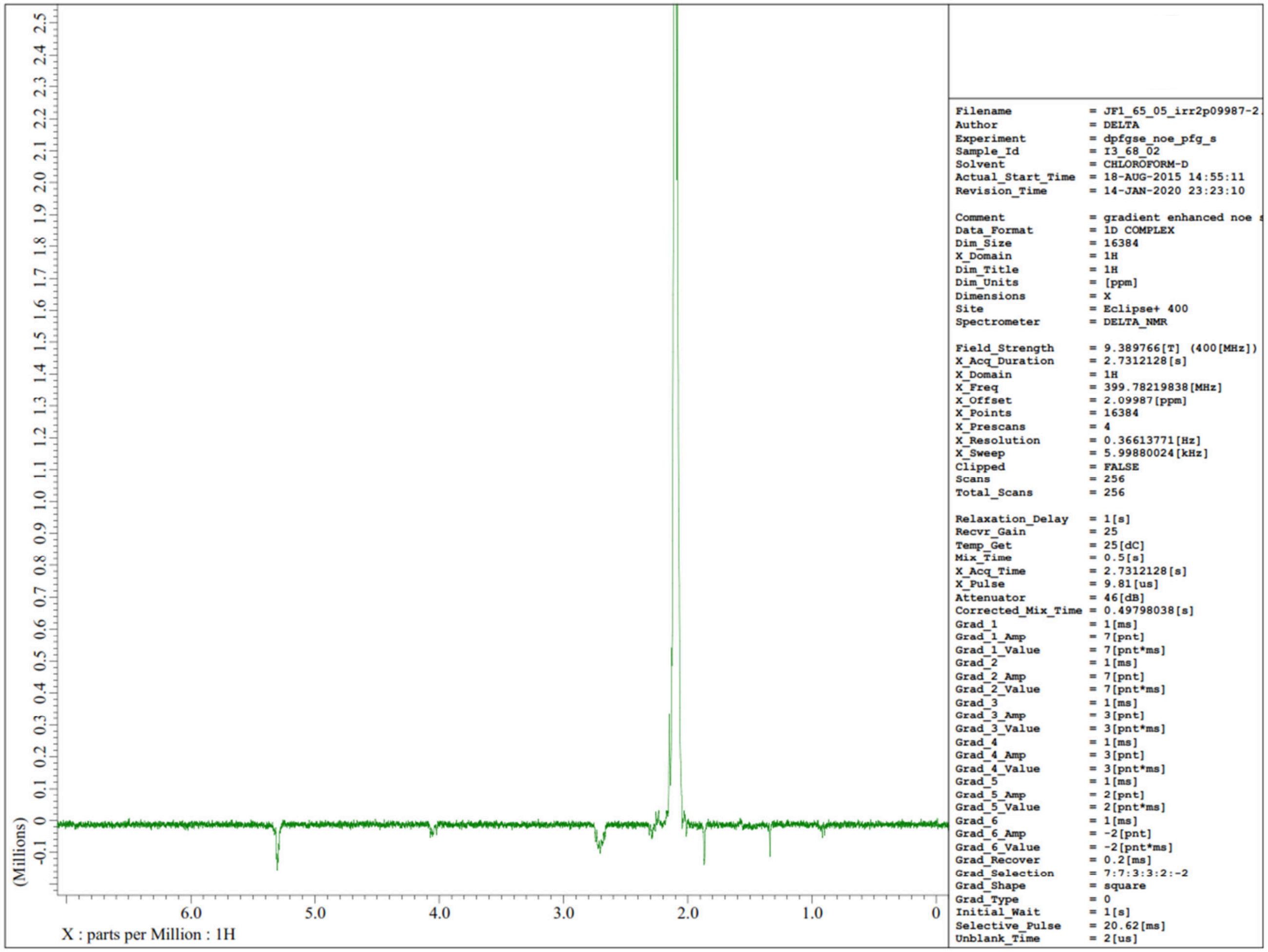

Figure S15. Asbestinin 27, NOE spectra

Irradiation of H-1

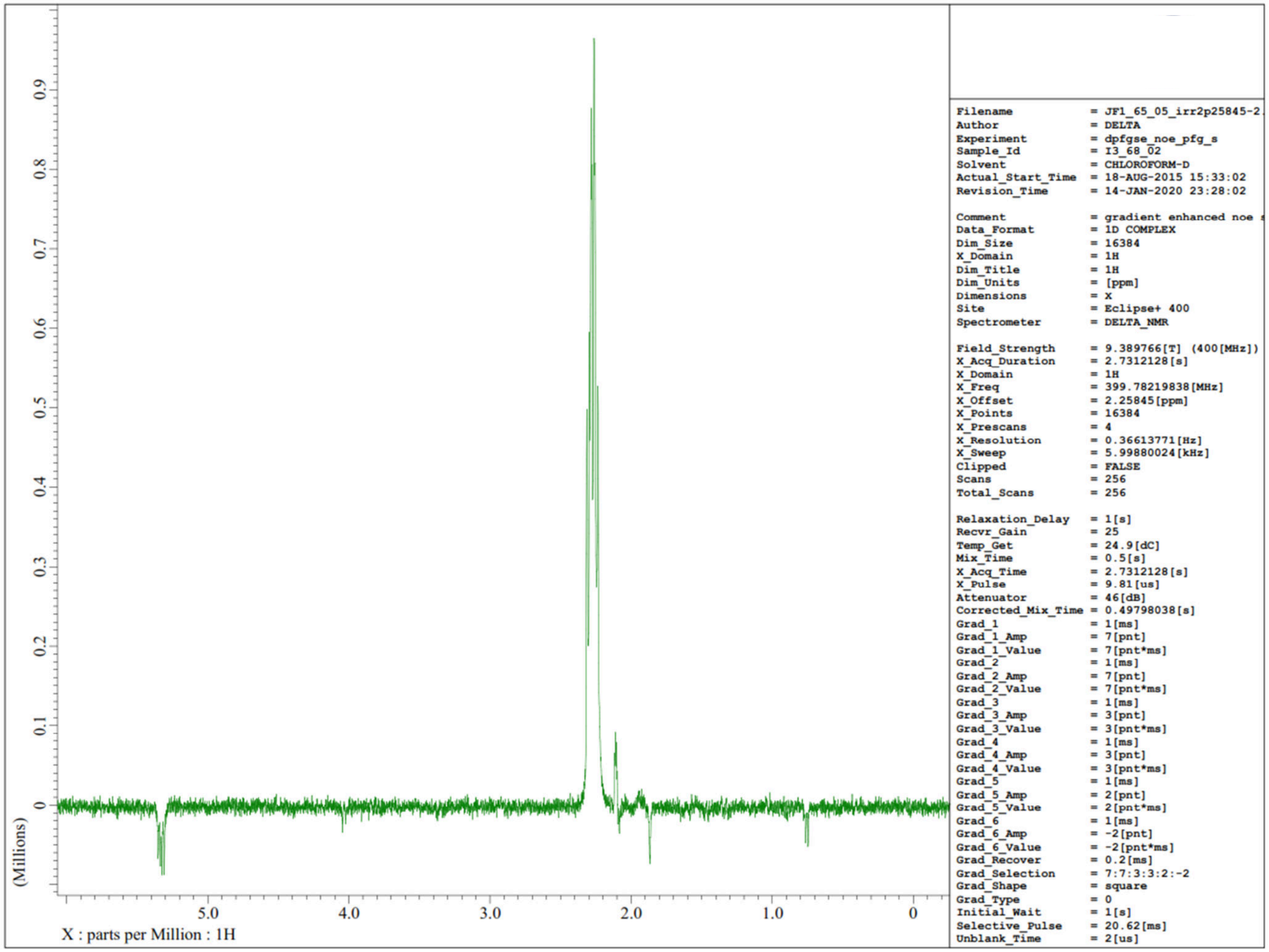

Figure S15. Asbestinin 27, NOE spectra

Irradiation of H-12

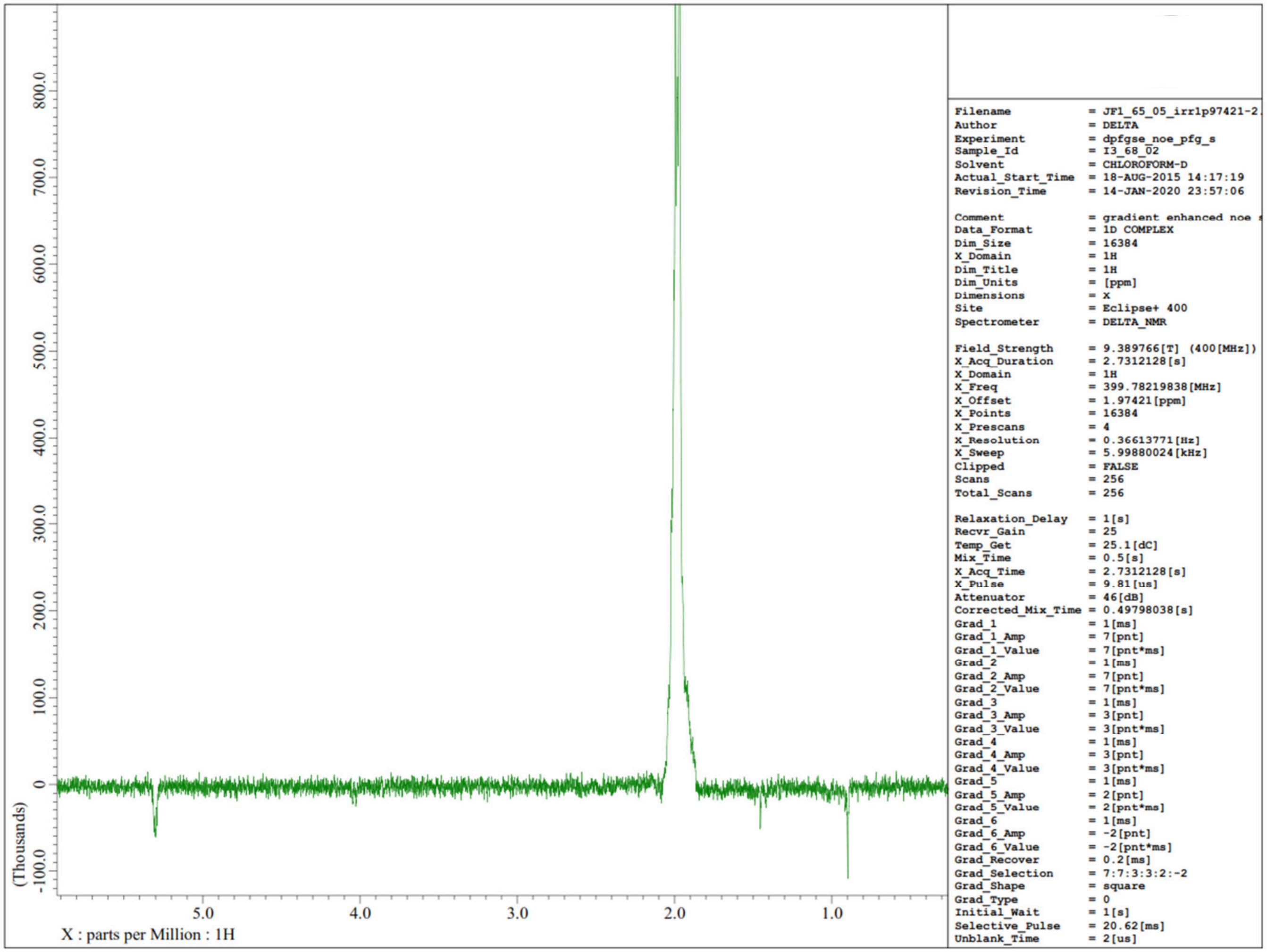

Figure S16. Asbestinin 27, HR-ESITOFMS spectra

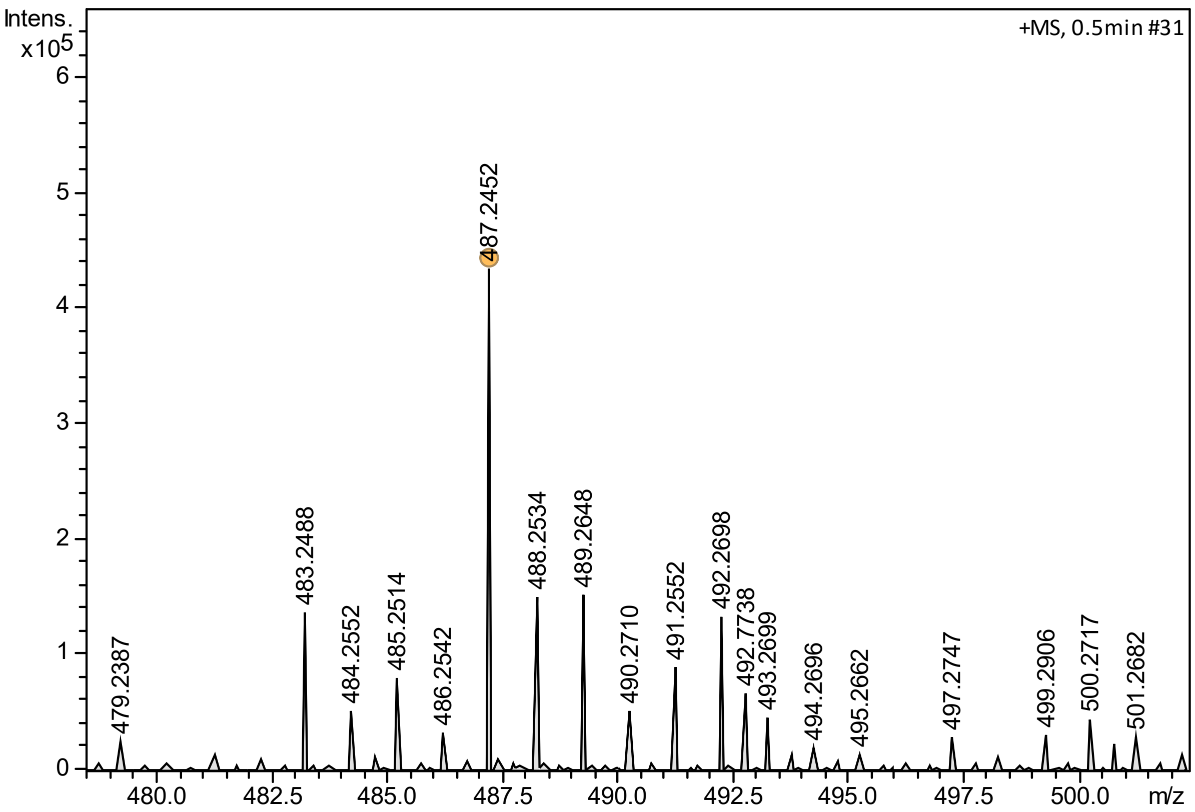

Figure S17. Asbestinin 28, ¹H NMR spectrum

```
Filename          = JF1_65_09-4.jdf
Author            = DELTA
Experiment        = single_pulse.exp
Sample_Id         = I3_62_05
Solvent           = CHLOROFORM-D
Actual_Start_Time =  3-AUG-2015 18:05:47
Revision_Time     = 22-AUG-2015 07:42:29

Comment           = Single Pulse Experiment,
Data_Format       = 1D COMPLEX
Dim_Size          = 16384
X_Domain          = 1H
Dim_Title         = 1H
Dim_Units         = [ppm]
Dimensions        = X
Site              = Eclipse+ 400
Spectrometer      = DELTA_NMR

Field_Strength    = 9.389766[T] (400[MHz])
X_Acq_Duration    = 2.048[s]
X_Domain          = 1H
X_Freq            = 399.78219838[MHz]
X_Offset          = 7[ppm]
X_Points          = 16384
X_Prescans        = 0
X_Resolution      = 0.48828125[Hz]
X_Sweep           = 8[kHz]
Clipped           = FALSE
Scans             = 8
Total_Scans       = 8

Relaxation_Delay  = 4[s]
Recvr_Gain        = 13
Temp_Get          = 24.7[dC]
X_90_Width        = 9.81[us]
X_Acq_Time        = 2.048[s]
X_Angle           = 45[deg]
X_Pulse           = 4.905[us]
Initial_Wait      = 1[s]
Unblank_Time      = 2[us]
```

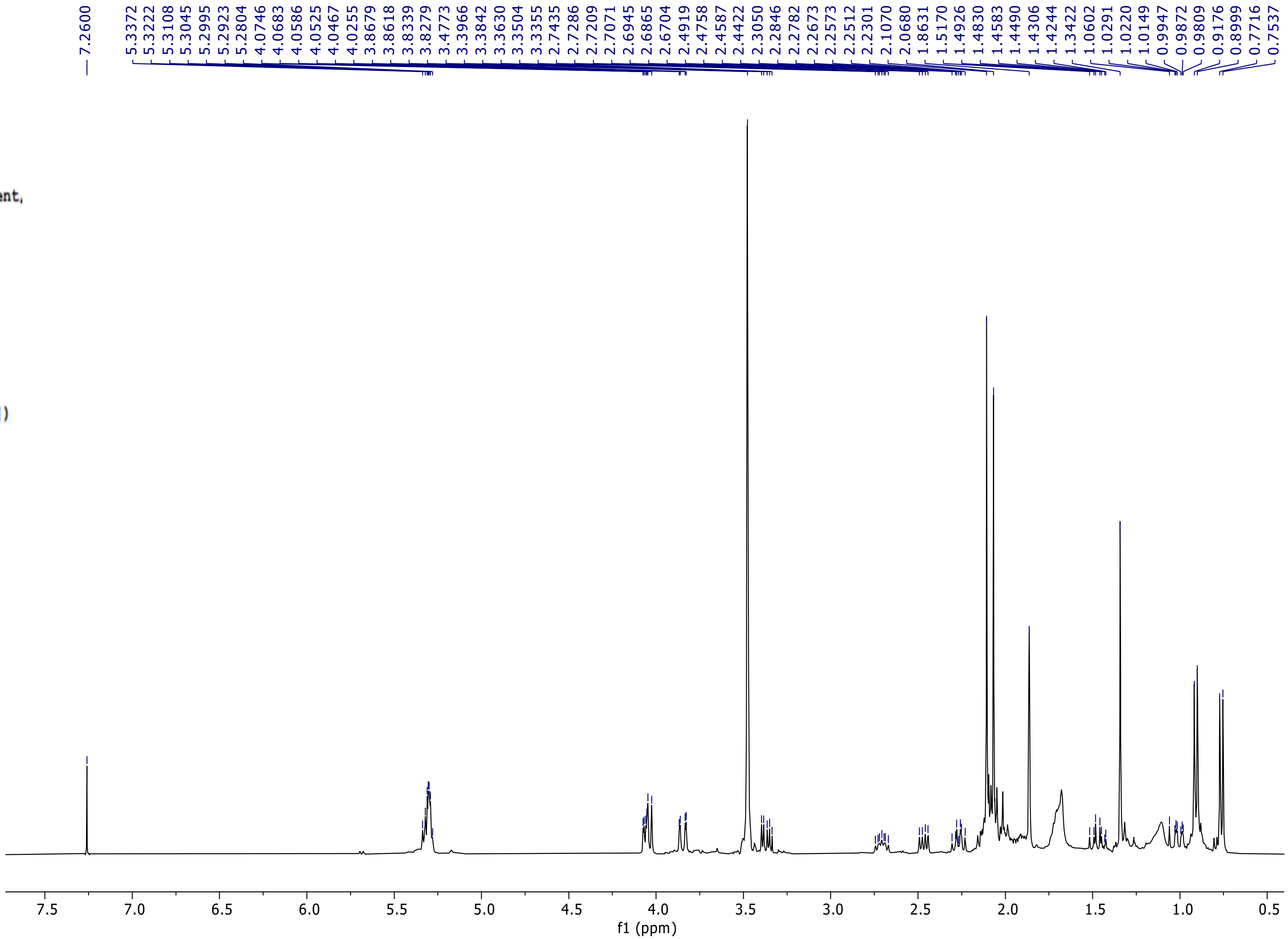

Figure S17. Asbestinin 28, ^{1}H NMR spectrum (expanded)

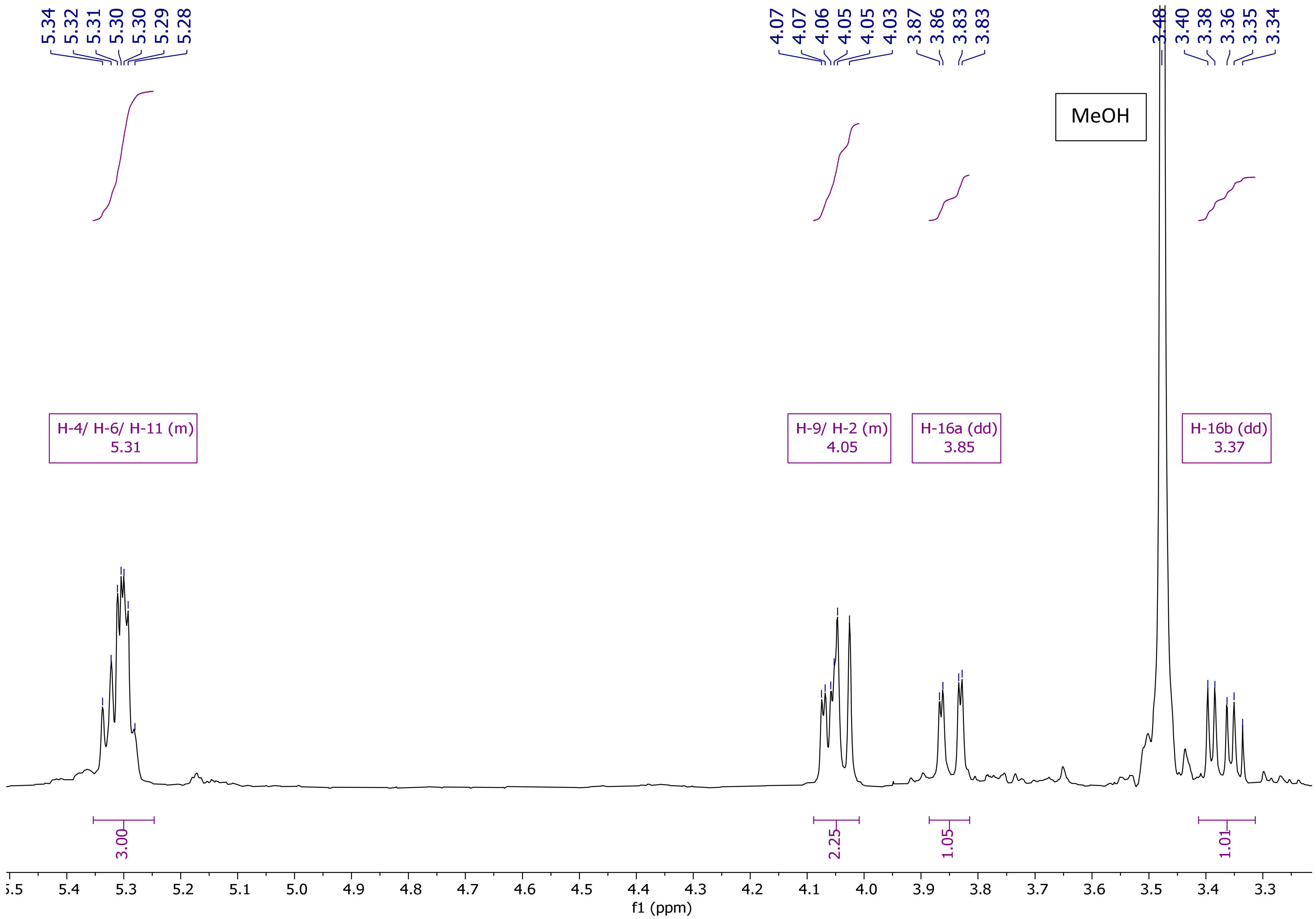

Figure S17. Asbestinin 28, ^{1}H NMR spectrum (expanded)

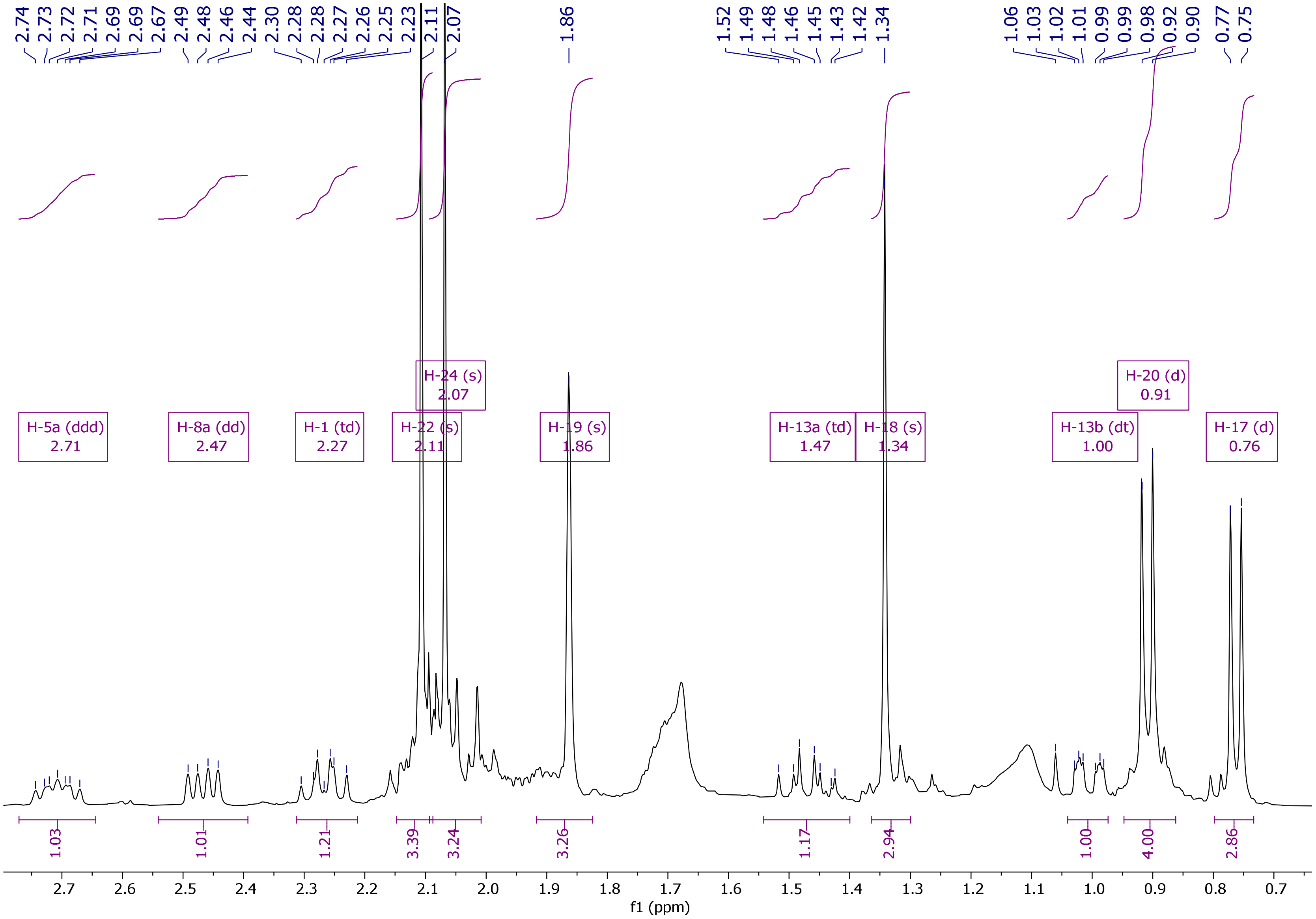

Figure S18. Asbestinin 28, ^{13}C NMR spectrum

```
Filename           = JF1_65_09 C13-3.jdf
Author             = DELTA
Experiment         = single_pulse_dec
Sample_Id          = I3_62_05
Solvent            = CHLOROFORM-D
Actual_Start_Time  =  3-AUG-2015 18:13:56
Revision_Time      =  5-AUG-2015 22:35:58

Comment            = Single Pulse with Broad
Data_Format        = 1D COMPLEX
Dim_Size           = 32768
X_Domain           = 13C
Dim_Title          = 13C
Dim_Units          = [ppm]
Dimensions         = X
Site               = Eclipse+ 400
Spectrometer       = DELTA_NMR

Field_Strength     = 9.389766[T] (400[MHz])
X_Acq_Duration     = 1.3008896[s]
X_Domain           = 13C
X_Freq             = 100.52530333[MHz]
X_Offset           = 100[ppm]
X_Points           = 32768
X_Prescans         = 4
X_Resolution       = 0.76870474[Hz]
X_Sweep            = 25.18891688[kHz]
Irr_Domain         = 1H
Irr_Freq           = 399.78219838[MHz]
Irr_Offset         = 5[ppm]
Clipped            = FALSE
Scans              = 7168
Total_Scans        = 7168

Relaxation_Delay   = 1[s]
Recvr_Gain         = 26
Temp_Get           = 25.9[dC]
X_90_Width         = 10[us]
X_Acq_Time         = 1.3008896[s]
X_Angle            = 30[deg]
X_Pulse            = 3.33333333[us]
Initial_Wait       = 1[s]
Unblank_Time       = 2[us]
```

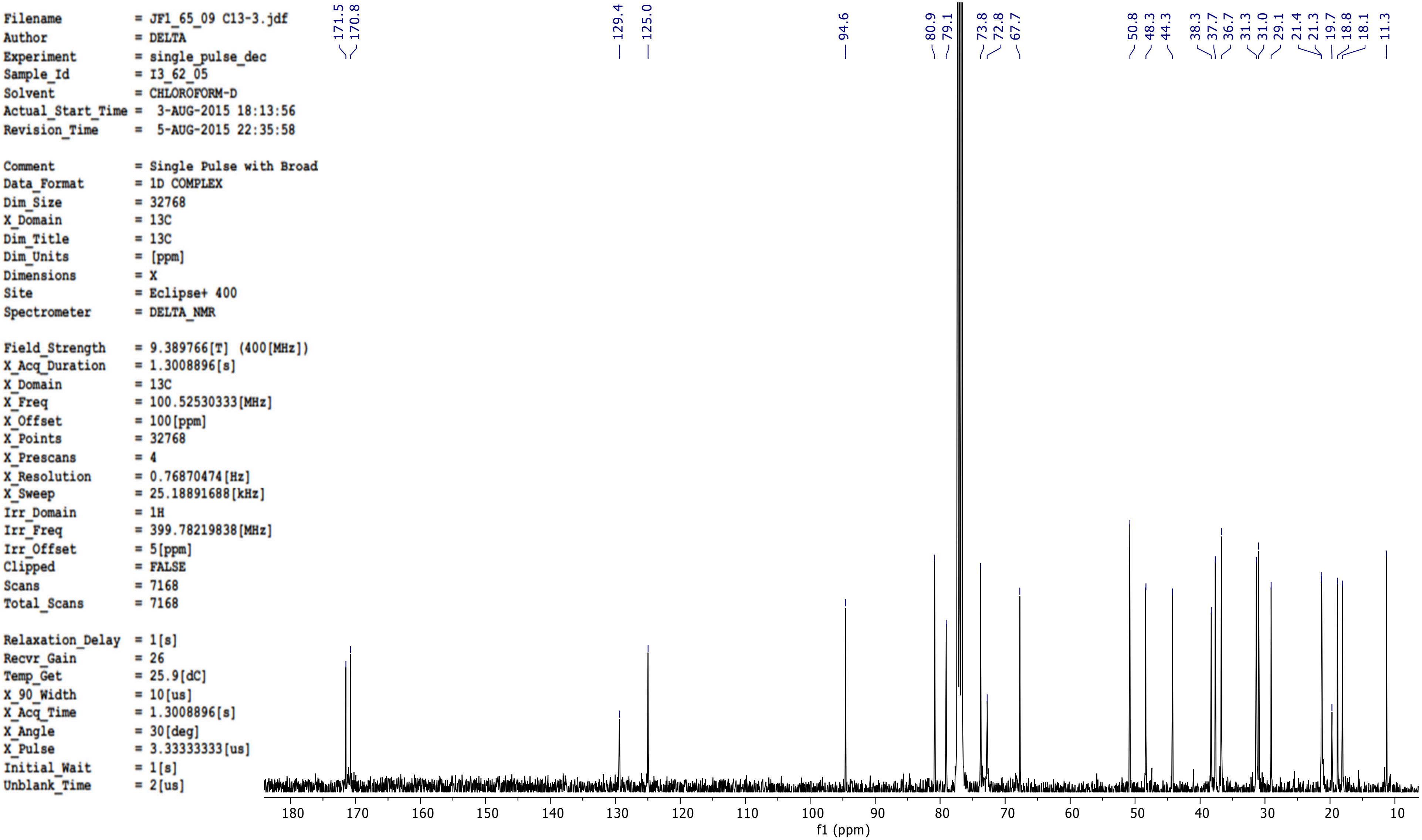

Figure S19. Asbestinin 28, DEPT-135 spectrum

Filename	= JF1_65_09 dept135-2.jdf
Author	= DELTA
Experiment	= dept_dec.exp
Sample_Id	= I3_62_05
Solvent	= CHLOROFORM-D
Actual_Start_Time	= 3-AUG-2015 22:49:50
Revision_Time	= 6-AUG-2015 01:26:36
Comment	= DEPT with decoupling
Data_Format	= 1D COMPLEX
Dim_Size	= 32768
X_Domain	= 13C
Dim_Title	= 13C
Dim_Units	= [ppm]
Dimensions	= X
Site	= Eclipse+ 400
Spectrometer	= DELTA_NMR
Field_Strength	= 9.389766[T] (400[MHz])
X_Acq_Duration	= 1.3008896[s]
X_Domain	= 13C
X_Freq	= 100.52530333[MHz]
X_Offset	= 100[ppm]
X_Points	= 32768
X_Prescans	= 4
X_Resolution	= 0.76870474[Hz]
X_Sweep	= 25.18891688[kHz]
Irr_Domain	= 1H
Irr_Freq	= 399.78219838[MHz]
Irr_Offset	= 5[ppm]
Clipped	= FALSE
Scans	= 3072
Total_Scans	= 3072
Relaxation_Delay	= 2[s]
Recvr_Gain	= 26
Temp_Get	= 24.9[dC]
X_Acq_Time	= 1.3008896[s]
X_Pulse	= 10[us]
Irr_Pulse	= 44.5[us]
Initial_Wait	= 1[s]
J_Constant	= 140[Hz]
Selection_Angle	= 135[deg]
Selection_Pulse	= 66.75[us]
Unblank_Time	= 2[us]

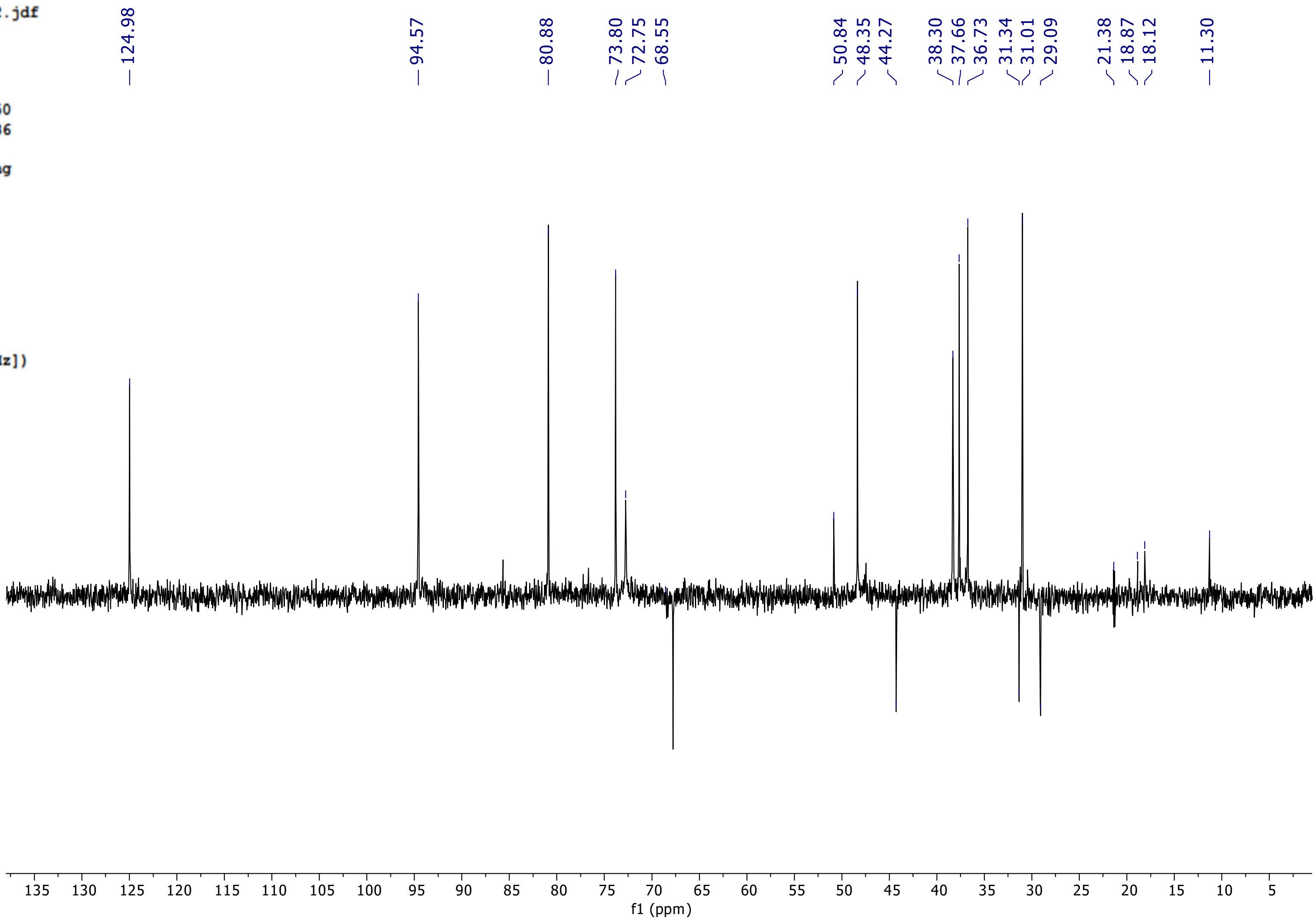

Figure S20. Asbestinin 28, COSY spectrum

```
Filename            = JF1_65_09 pfgcosy-2.jdf
Author              = DELTA
Experiment          = cosy_pfg_s.exp
Sample_Id           = I3_62_05
Solvent             = CHLOROFORM-D
Actual_Start_Time   =  4-AUG-2015 14:37:02
Revision_Time       =  6-AUG-2015 15:54:25

Comment             = gradient absolute value
Data_Format         = 2D REAL REAL
Dim_Size            = 1024, 1024
X_Domain            = 1H
Y_Domain            = 1H
Dim_Title           = 1H 1H
Dim_Units           = [ppm] [ppm]
Dimensions          = X Y
Site                = Eclipse+ 400
Spectrometer        = DELTA_NMR

Field_Strength      = 9.389766[T] (400[MHz])
X_Acq_Duration      = 0.2832384[s]
X_Domain            = 1H
X_Freq              = 399.78219838[MHz]
X_Offset            = 3.76611[ppm]
X_Points            = 1024
X_Prescans          = 4
X_Resolution        = 3.53059472[Hz]
X_Sweep             = 3.61532899[kHz]
Y_Domain            = 1H
Y_Freq              = 399.78219838[MHz]
Y_Offset            = 3.76611[ppm]
Y_Points            = 256
Y_Prescans          = 0
Y_Resolution        = 14.12237889[Hz]
Y_Sweep             = 3.61532899[kHz]
Clipped             = TRUE
Scans               = 16
Total_Scans         = 4096

Relaxation_Delay    = 1[s]
Recvr_Gain          = 22
Temp_Get            = 25.8[dC]
X_90_Width          = 9.81[us]
X_Acq_Time          = 0.2832384[s]
X_Pulse             = 9.81[us]
Y_Acq_Time          = 70.8096[ms]
Grad_1              = 1[ms]
Grad_1_Amp          = 5[pnt]
Grad_1_Value        = 5[pnt*ms]
Grad_2              = 1[ms]
Grad_2_Amp          = 5[pnt]
Grad_2_Value        = 5[pnt*ms]
Grad_Recover        = 1[ms]
Grad_Selection      = 1:1
Grad_Shape          = square
Grad_Type           = 0
Initial_Wait        = 0.1[s]
Pulse_1             = 9.81[us]
Pulse_2             = 9.81[us]
Pulse_Angle_1       = 90[deg]
Pulse_Angle_2       = 90[deg]
T1                  = 1[us]
Unblank_Time        = 2[us]
```

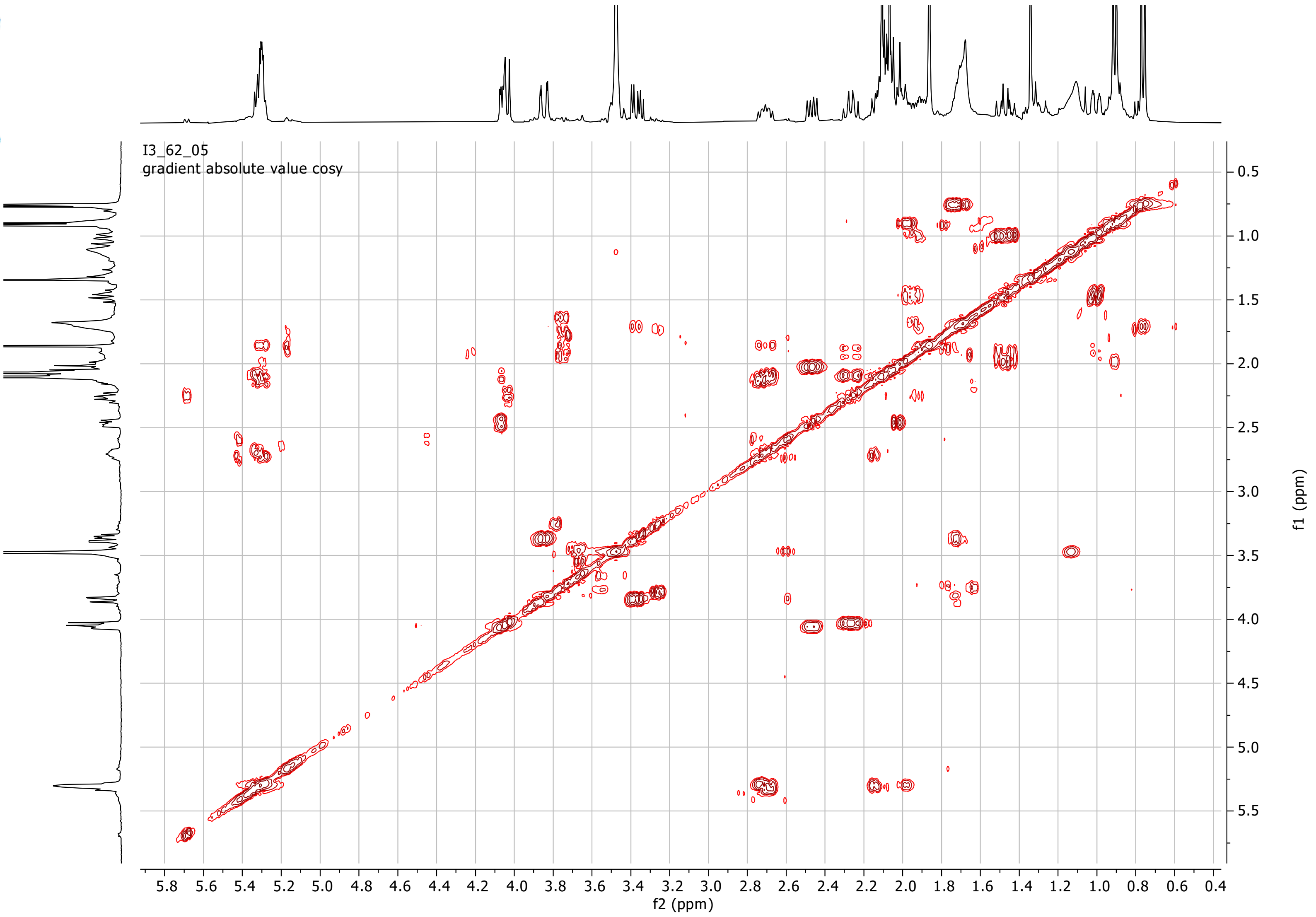

Figure S21. Asbestinin 28, HSQC spectrum

```
Filename            = JF1_65_09 hsqc-4.jdf
Author              = DELTA
Experiment          = multiplicity_hsq
Sample_Id           = I3_62_05
Solvent             = CHLOROFORM-D
Actual_Start_Time   =  4-AUG-2015 04:31:29
Revision_Time       = 21-AUG-2015 06:18:42

Comment             = Multiplicity HSQC
Data_Format         = 2D COMPLEX COMPLEX
Dim_Size            = 1024, 512
X_Domain            = 1H
Y_Domain            = 13C
Dim_Title           = 1H 13C
Dim_Units           = [ppm] [ppm]
Dimensions          = X Y
Site                = Eclipse+ 400
Spectrometer        = DELTA_NMR

Field_Strength      = 9.389766[T] (400[MHz])
X_Acq_Duration      = 0.2832384[s]
X_Domain            = 1H
X_Freq              = 399.78219838[MHz]
X_Offset            = 3.76611[ppm]
X_Points            = 1024
X_Prescans          = 4
X_Resolution        = 3.53059472[Hz]
X_Sweep             = 3.61532899[kHz]
Y_Domain            = 13C
Y_Freq              = 100.52530333[MHz]
Y_Offset            = 100[ppm]
Y_Points            = 256
Y_Prescans          = 0
Y_Resolution        = 98.64267677[Hz]
Y_Sweep             = 25.25252525[kHz]
Clipped             = FALSE
Scans               = 8
Total_Scans         = 2048

Relaxation_Delay    = 2[s]
Recvr_Gain          = 30
Temp_Get            = 24.1[dC]
X_Acq_Time          = 0.2832384[s]
X_Pulse             = 9.81[us]
Y_Acq_Time          = 10.1376[ms]
Y_Pulse             = 12[us]
Enhance_Temp        = 12
Enhancement         = 1/6J
Grad_1              = 1[ms]
Grad_1_Amp          = 4[pnt]
Grad_1_Value        = 4[pnt*ms]
Grad_2              = 1[ms]
Grad_2_Amp          = -1[pnt]
Grad_2_Value        = -1[pnt*ms]
Grad_3              = 1[ms]
Grad_3_Amp          = 1[pnt]
Grad_Recover        = 0.1[ms]
Grad_Selection      = 13C = 4:1
Grad_Shape          = square
Grad_Type           = 0
Initial_Wait        = 1[s]
J_Constant          = 140[Hz]
T1                  = 1[us]
Tau                 = 0.5952381[ms]
```

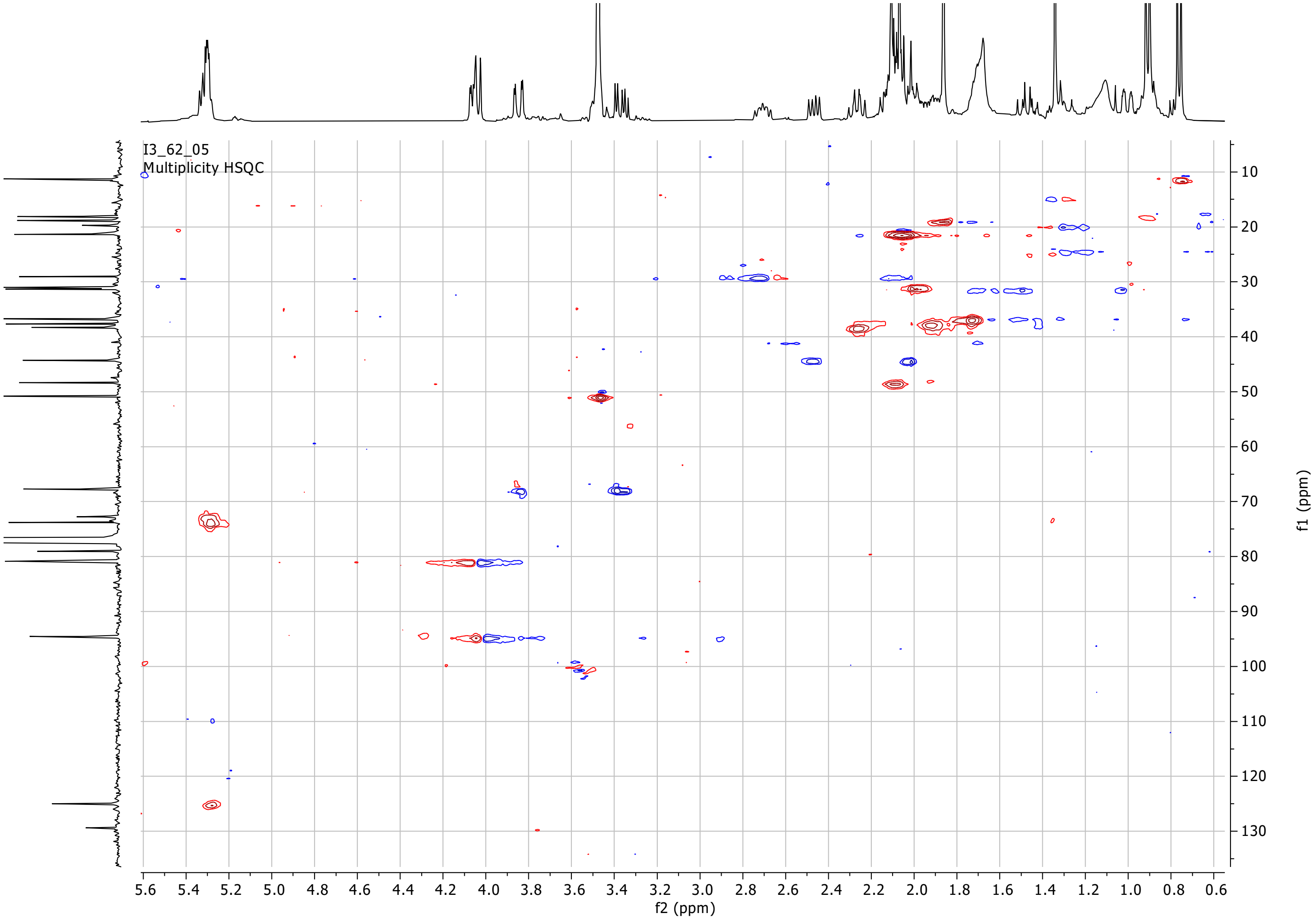

Figure S22. Asbestinin 28, HMBC spectrum

```
Filename          = JF1_65_09_210815_hmbc-3
Author            = DELTA
Experiment        = hmbc_pfg_s.exp
Sample_Id         = I3_68_06
Solvent           = CHLOROFORM-D
Actual_Start_Time = 19-AUG-2015 15:04:21
Revision_Time     = 22-AUG-2015 01:38:21

Comment           = gradient enhanced HMBC
Data_Format       = 2D REAL REAL
Dim_Size          = 1024, 512
X_Domain          = 1H
Y_Domain          = 13C
Dim_Title         = 1H 13C
Dim_Units         = [ppm] [ppm]
Dimensions        = X Y
Site              = Eclipse+ 400
Spectrometer      = DELTA_NMR

Field_Strength    = 9.389766[T] (400[MHz])
X_Acq_Duration    = 0.2961408[s]
X_Domain          = 1H
X_Freq            = 399.78219838[MHz]
X_Offset          = 3.59946[ppm]
X_Points          = 1024
X_Prescans        = 4
X_Resolution      = 3.37677213[Hz]
X_Sweep           = 3.45781466[kHz]
Y_Domain          = 13C
Y_Freq            = 100.52530333[MHz]
Y_Offset          = 100[ppm]
Y_Points          = 256
Y_Prescans        = 0
Y_Resolution      = 98.64267677[Hz]
Y_Sweep           = 25.25252525[kHz]
Clipped           = FALSE
Scans             = 64
Total_Scans       = 16384

Relaxation_Delay  = 2[s]
Recvr_Gain        = 30
Temp_Get          = 23.5[dC]
X_Acq_Time        = 0.2961408[s]
X_Pulse           = 9.81[us]
Y_Acq_Time        = 10.1376[ms]
Y_Pulse           = 12[us]
Grad_1            = 1[ms]
Grad_1_Amp        = 10[pnt]
Grad_1_Value      = 10[pnt*ms]
Grad_2            = 1[ms]
Grad_2_Amp        = 10[pnt]
Grad_2_Value      = 10[pnt*ms]
Grad_3            = 1[ms]
Grad_3_Amp        = 5[pnt]
Grad_3_Value      = 5[pnt*ms]
Grad_Recover      = 0.2[ms]
Grad_Selection    = 13C = 2:2:1
Grad_Shape        = square
Grad_Type         = 0
Initial_Wait      = 1[s]
J_Constant        = 140[Hz]
Long_Range_J      = 8[Hz]
T1                = 1[us]
Unblank_Time      = 2[us]
```

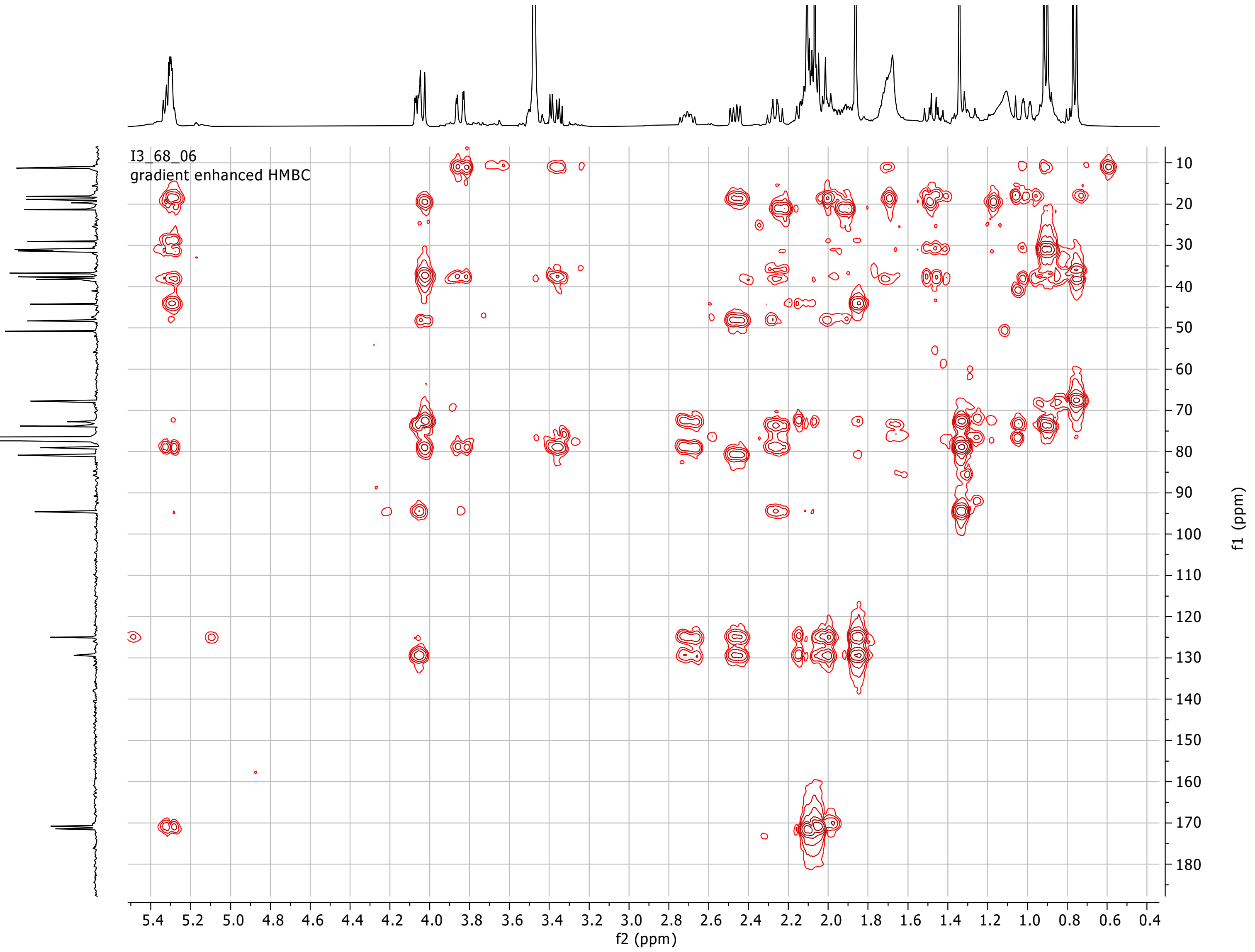

Figure S23. Asbestinin 28, NOE spectra

Irradiation of Proton H-17

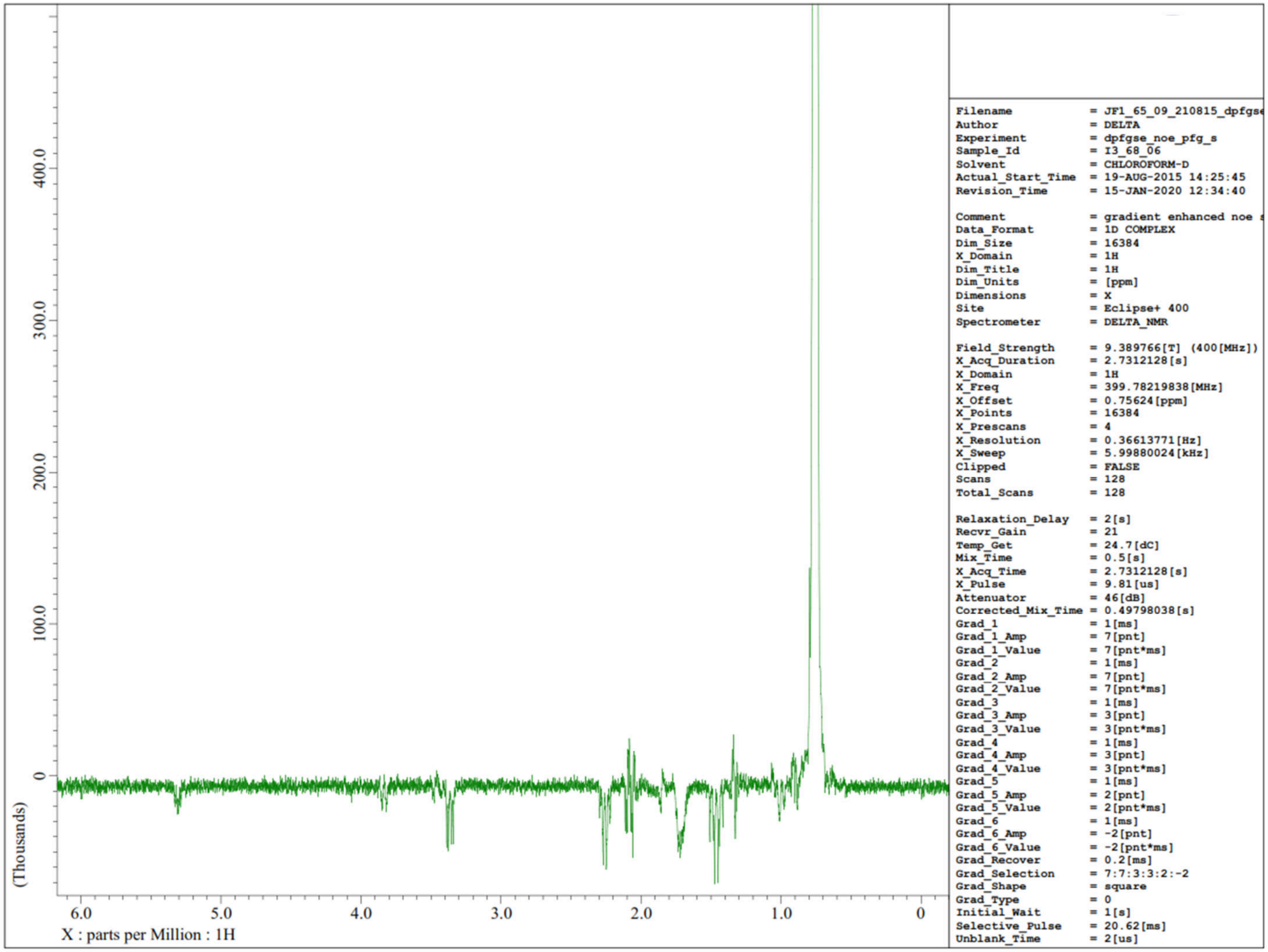

Figure S23. Asbestinin 28, NOE spectra

Irradiation of Proton H-18

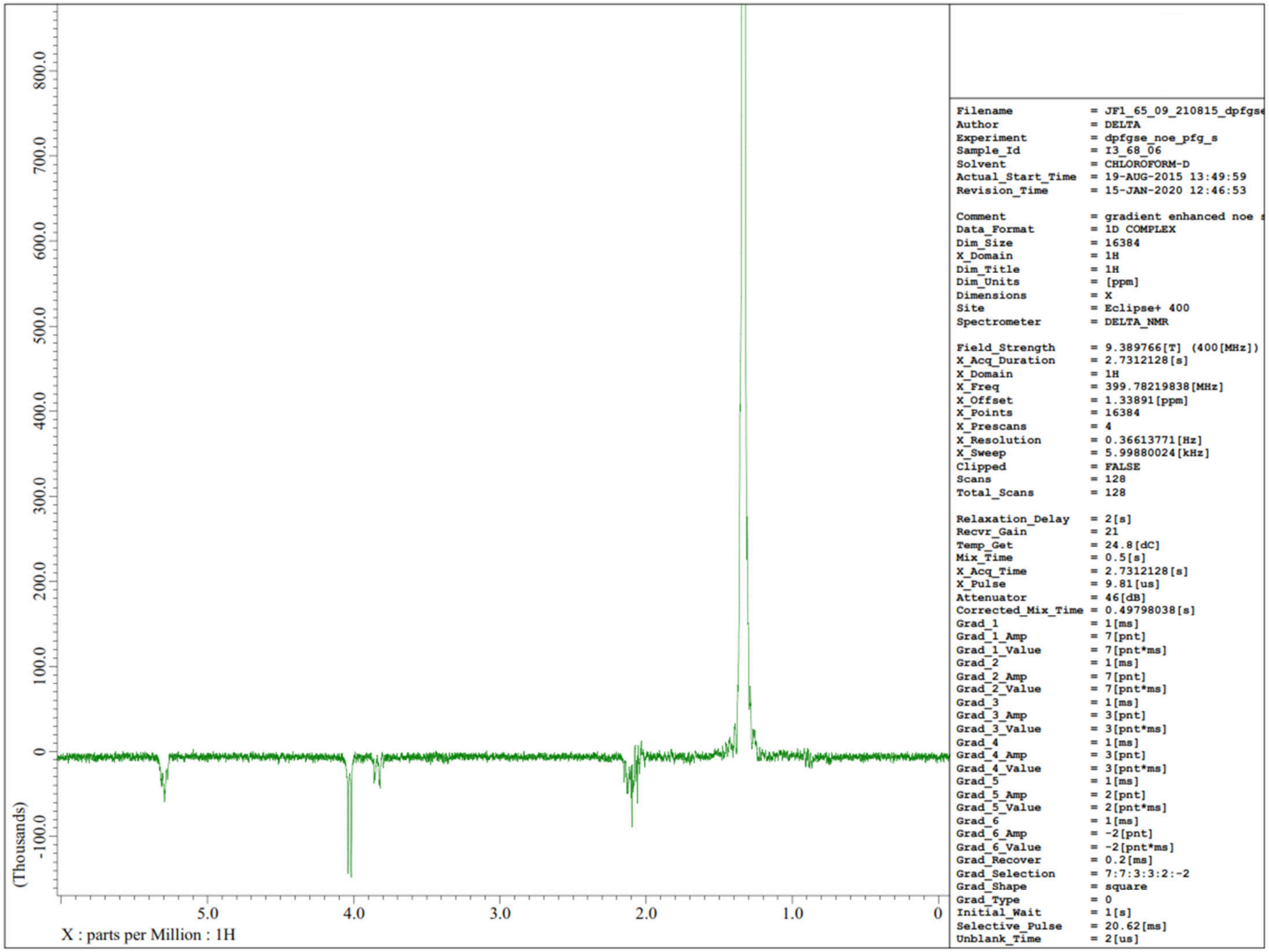

Figure S23. Asbestinin 28, NOE spectra

Irradiation of Proton H-19

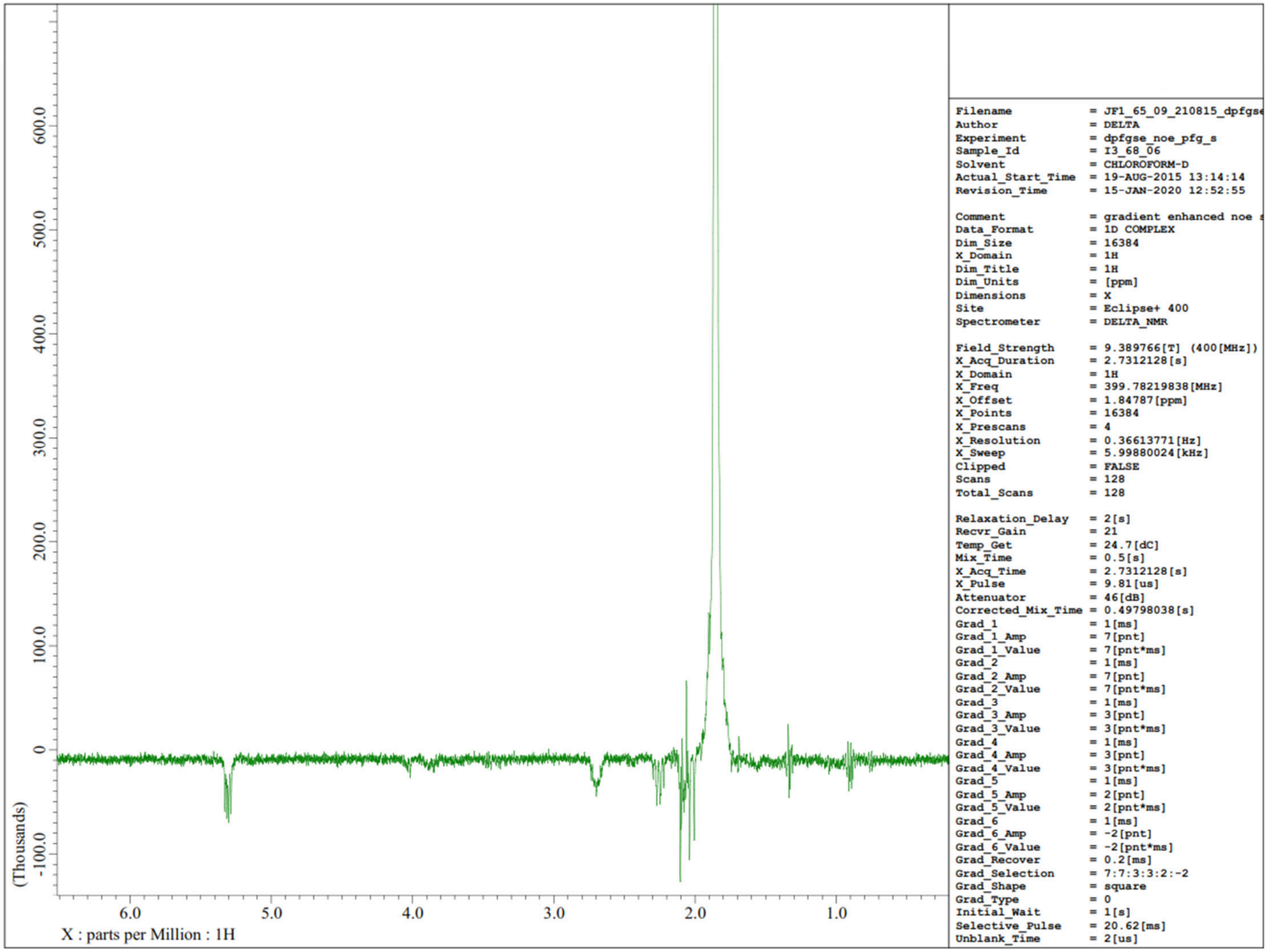

Figure S23. Asbestinin 28, NOE spectra

Irradiation of Proton H-14

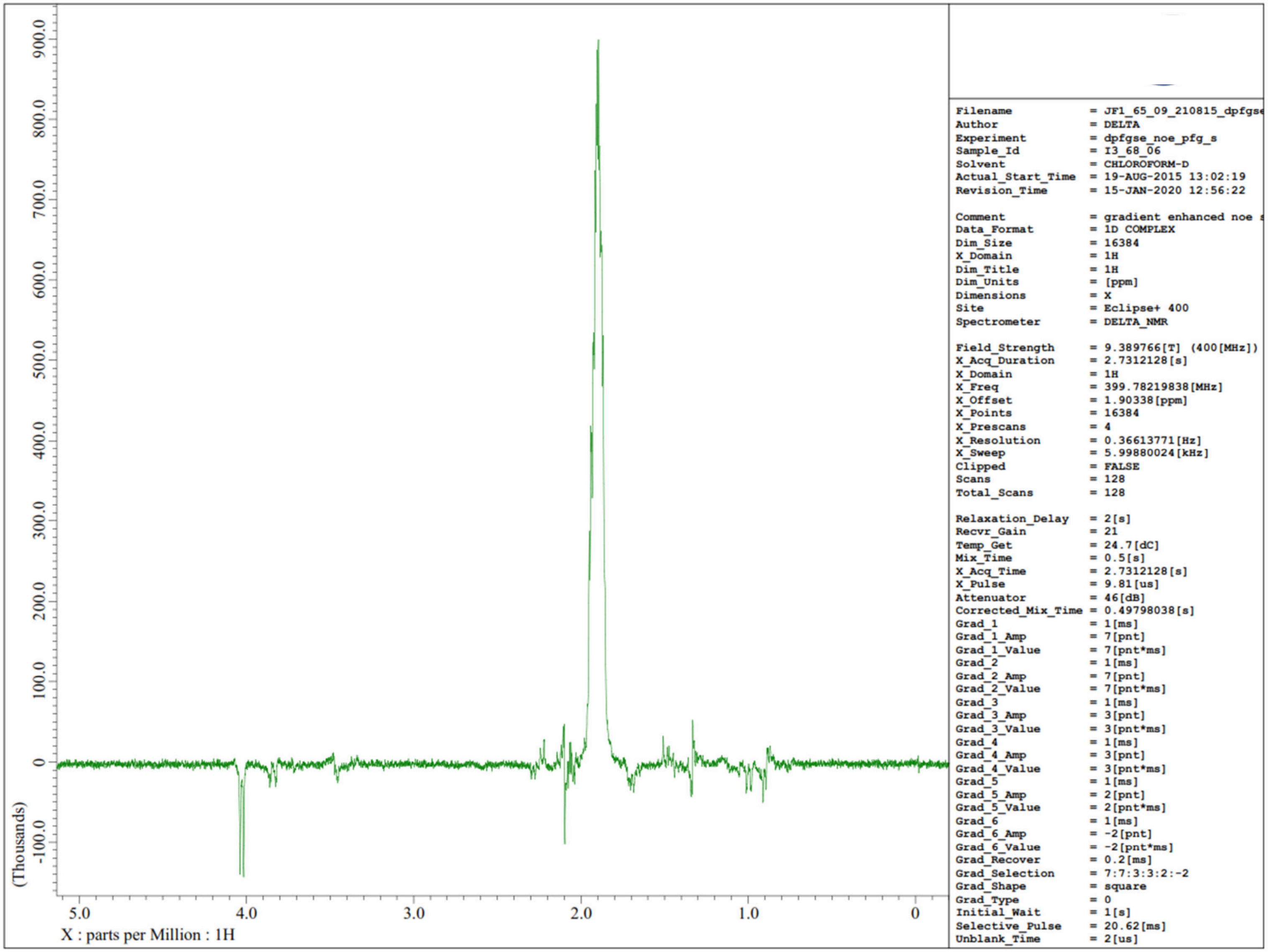

Figure S23. Asbestinin 28, NOE spectra

Irradiation of Proton H-1

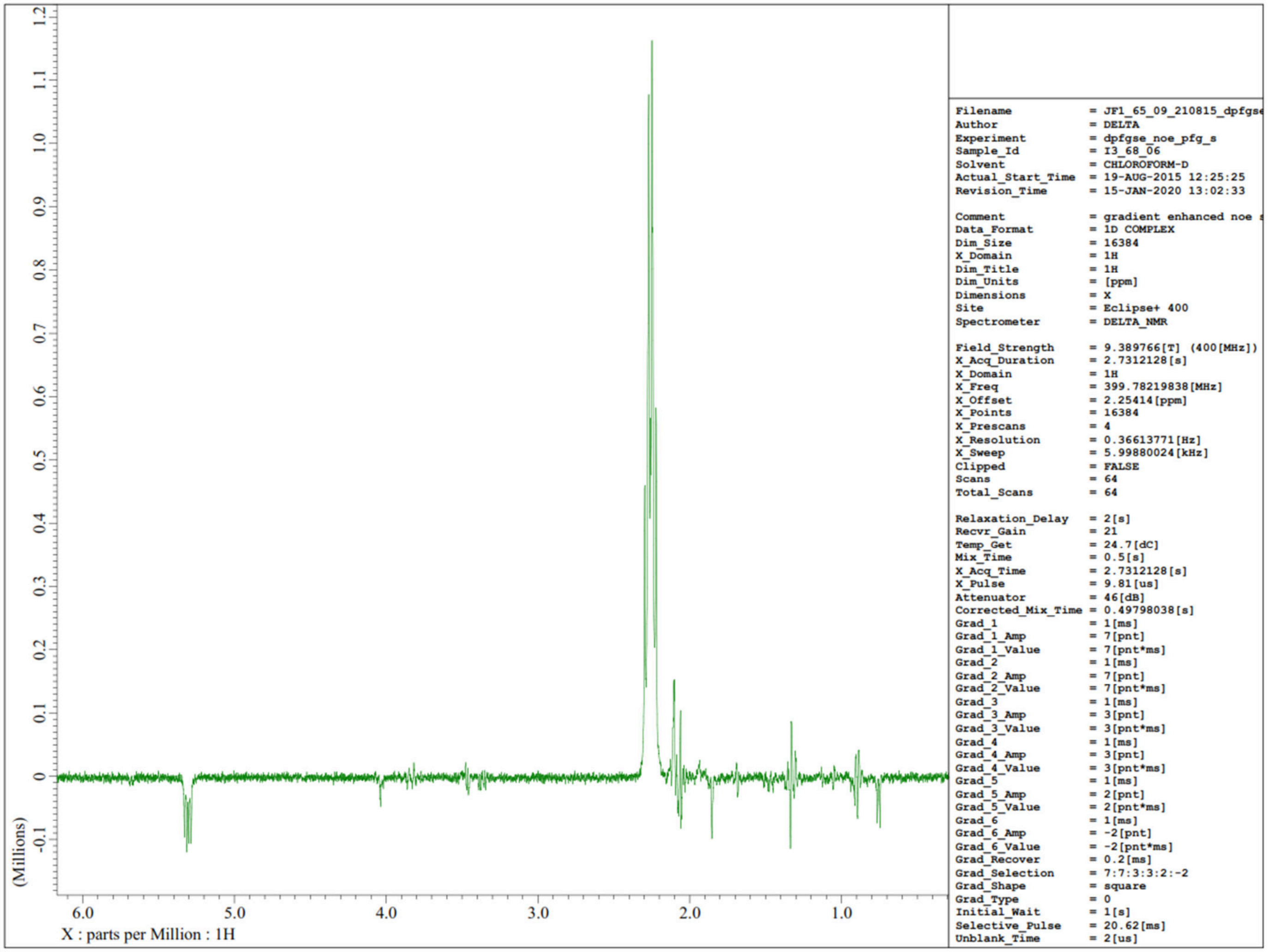

Figure S23. Asbestinin 28, NOE spectra

Irradiation of Proton H-2

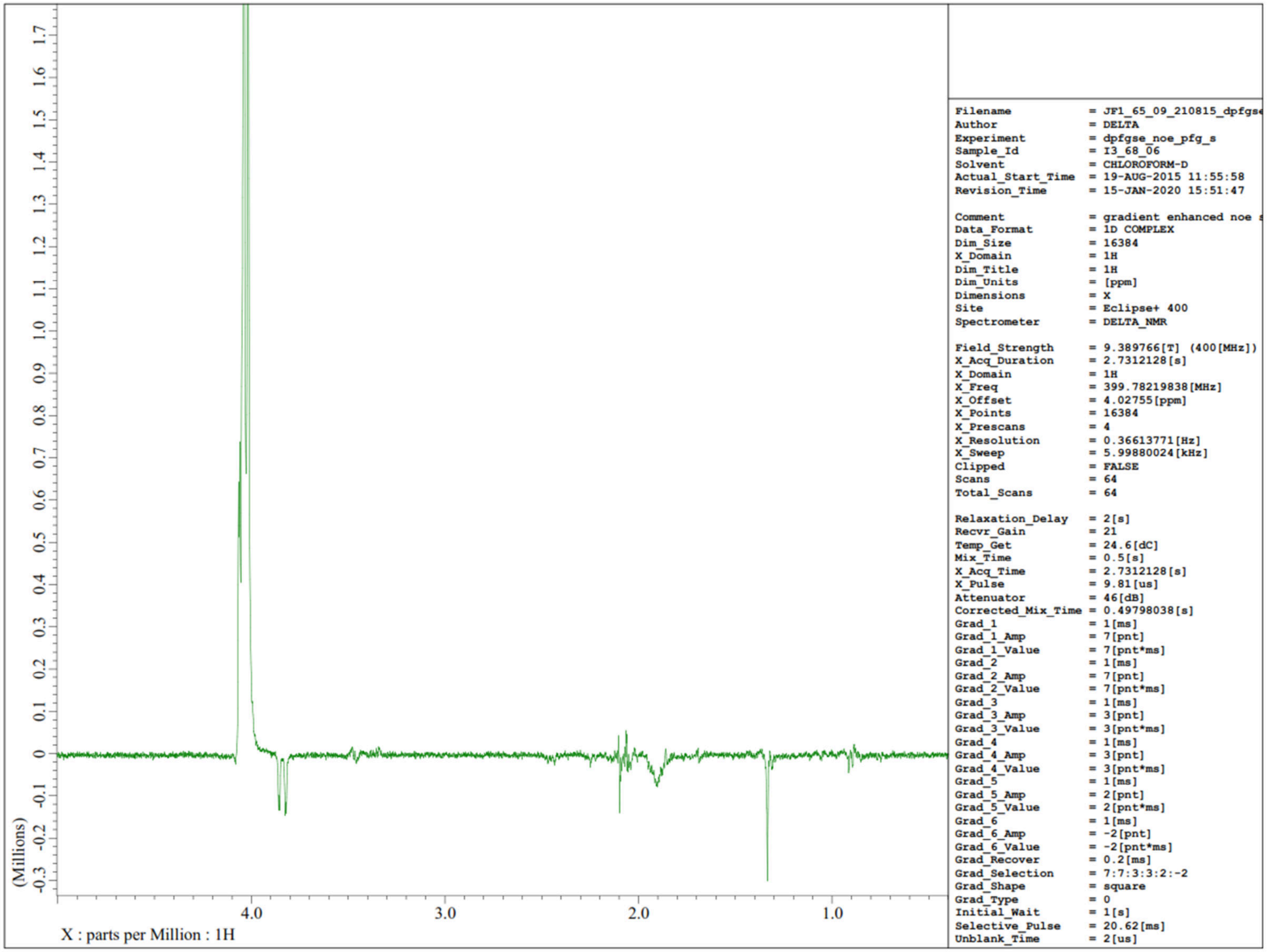

Figure S23. Asbestinin 28, NOE spectra

Irradiation of Proton H-9

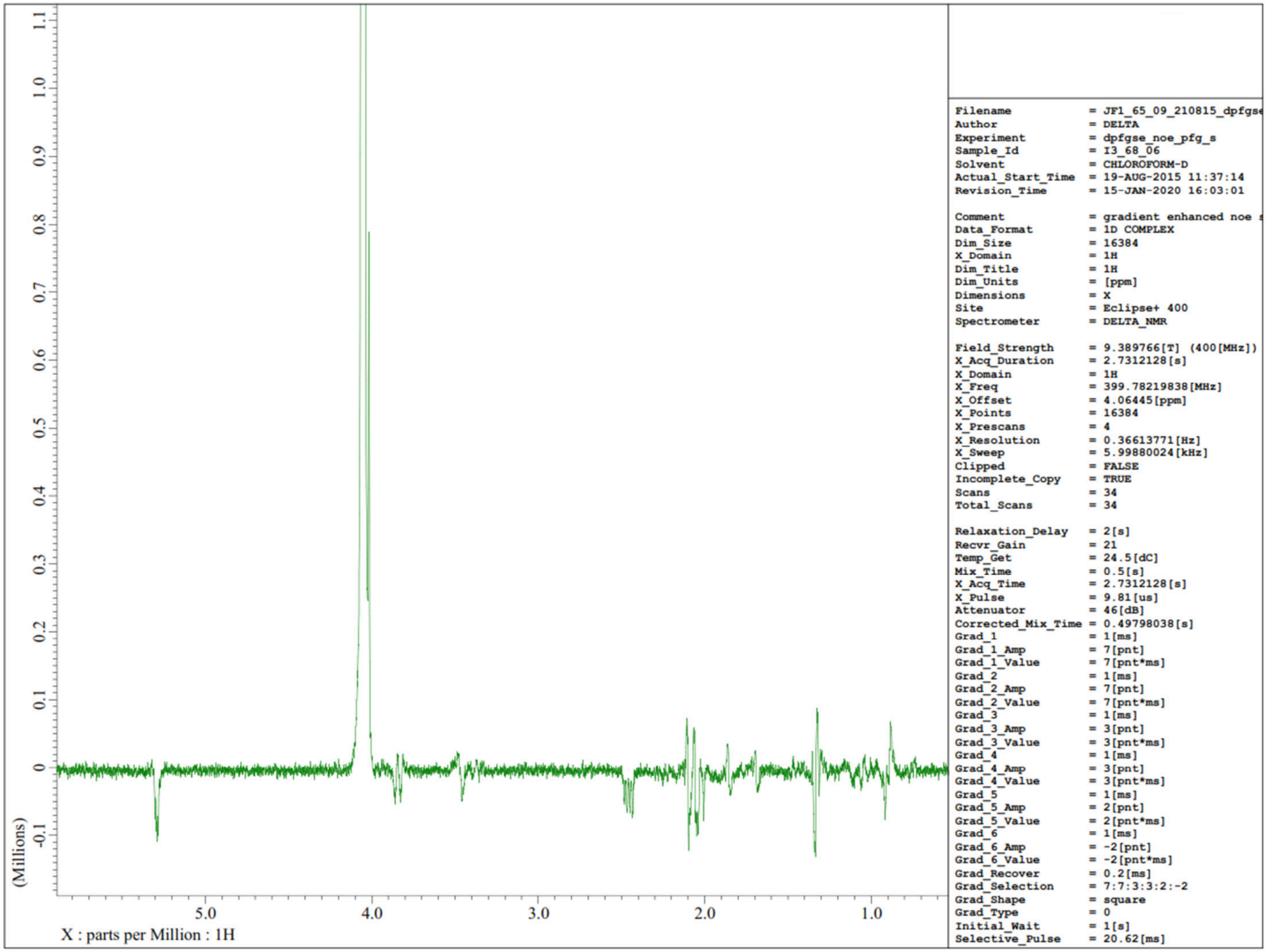

Figure S24. Asbestinin 28, HR-ESITOFMS spectrum

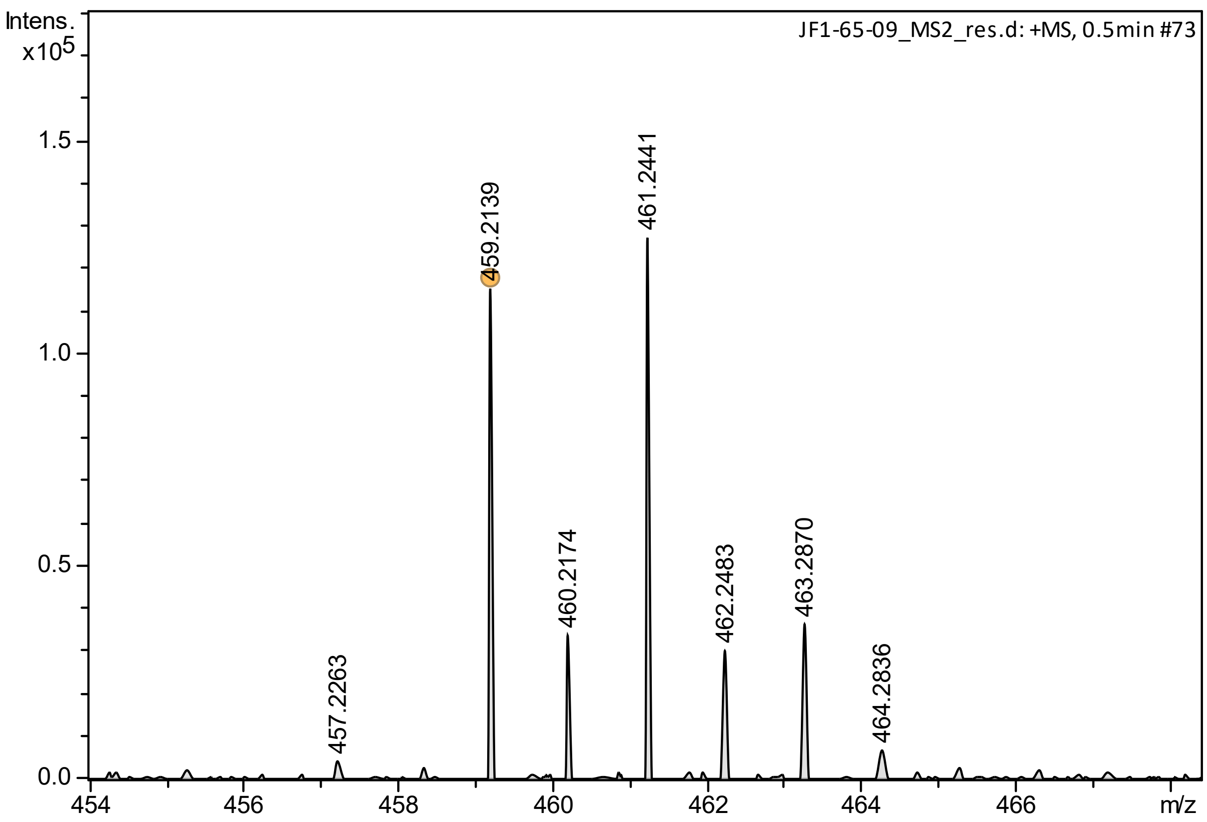

Table S1. Viability of THP-1 human macrophages treated with different concentrations of diterpenes. Values are mean ± SEM (%) of three independent experiments in duplicate (n = 3).

	% Viability THP-1 macrophages (24 h)				
(μM)	10	20	50	100	IC_{50}
Briarellin T **(1)**	99.5 ± 2.0	98.5 ± 1.2	101.4 ± 0.5	98.5 ± 1.0	> 100
Asbestinin 27 **(2)**	100.5 ± 1.3	99.5 ± 0.9	98.6 ± 2.1	97.4 ± 1.1	> 100
Asbestinin 28 **(3)**	98.5 ± 3.1	99.1 ± 1.4	97.0 ± 1.0	99.5 ± 2.0	> 100
Asbestinin 17 **(4)**	102.2 ± 1.5	100.5 ± 0.8	98.0 ± 2.0	99.5 ± 1.2	> 100
